# Global origins of tuna and billfish fisheries reveal the transition from coastal access to oceanic exploitation

**DOI:** 10.1073/pnas.2512066123

**Published:** 2026-10-16

**Authors:** Torben C. Rick, Adam J. Andrews, Philippe Béarez, Marcella Festa, Thiago Fossile, Mark Hudson, Melanie Janßen-Kim, Maria José Juan-Jordá, Ariana B. J. Lambrides, Michelle J. LeFebvre, Alisa Luthra, Bonnie Newsom, Sue O’Connor, Eréndira Quintana Morales, Arthur Spiess, Claudia Zancan, Caitlin Zimmer, Emma A. Elliott Smith

**Affiliations:** ^a^ Department of Anthropology, National Museum of Natural History, Smithsonian Institution, Washington, DC 20013-7012; ^b^ Section for Marine Biology, Norwegian Institute for Water Research, Oslo 0579, Norway; ^c^ UMR 7209–BioArch, Muséum National d’Histoire Naturelle/CNRS, Paris 75005, France; ^d^School of Cultural Heritage, Northwest University, Xi’an 710127, People’s Republic of China; ^e^ Department of Ecology and Zoology, Universidade Federal de Santa Catarina, Florianópolis- SC 88035-972, Brazil; ^f^ Language and the Anthropocene Research Group, Max Planck Institute of Geoanthropology, Jena 07745, Germany; ^g^ Institut für Koreastudien, Freie Universität Berlin, Berlin 14195, Germany; ^h^ Instituto Español de Oceanografía–Consejo Superior de Investigaciones Científicas, Madrid 28002, Spain; ^i^ Australian Research Council Centre of Excellence for Indigenous and Environmental Histories and Futures, College of Arts, Society and Education, James Cook University, Cairns, QLD 4870, Australia; ^j^ Florida Museum of Natural History and Department of Anthropology, University of Florida, Gainesville, FL 32611; ^k^ Department of Anthropology and Climate Change Institute, University of Maine, Orono, ME 04469; ^l^School of Culture, History and Language, College of Asia and the Pacific, The Australian National University, Canberra, ACT 2601, Australia; ^m^ Anthropology Department, University of California, Santa Cruz, CA 95064; ^n^Maine Historic Preservation Commission, Augusta, ME 04333; ^o^ Department of Asian and North African Studies, Ca’ Foscari University of Venice, Venice 30123, Italy; ^p^Sainsbury Institute for the Study of Japanese Arts and Cultures, Norwich NR1 4DH, United Kingdom; ^q^ Department of Environmental Sciences, University of Virginia, Charlottesville, VA 22904; ^r^ Department of Biology and Center for Stable Isotopes, University of New Mexico, Albuquerque, NM 87131

**Keywords:** archaeology, historical ecology, Thunnini, Istiophoridae, Xiphiidae

## Abstract

Ocean ecosystems have played a crucial role in people’s lives around the world for millennia. However, questions persist about when, why, and how people first began removing biomass from offshore habitats and established fisheries in distant, oceanic environments. We explore the antiquity and evolution of tuna (Thunnini) and billfish (Istiophoridae/Xiphiidae) fisheries, two groups of fishes that occur around the world, are economically significant, and are ecologically important open ocean apex predators. The earliest archaeological evidence of fishing for tuna occurs ~44,000 years ago and billfish at ~10,000 years ago, with early fisheries for some tuna species appearing around the world by 3,000 to 1,000 years ago. Most early fisheries are in areas where tunas and billfishes could be accessed relatively close to shore (e.g., submarine canyons) and/or where these fishes may come inshore seasonally or during warm ocean events. Early tuna and billfish fisheries were often linked to ceremony/ritual, alongside advances in maritime technologies. With few exceptions, sustained and intensive commercial exploitation of pelagic predators in offshore and oceanic habitats is a late development in the human past, occurring primarily during the last 200 y. Our findings document a long-term continuum of human interactions with global marine ecosystems spanning thousands of years, including significant differences between the timing and pathways by which people became dominant ecological drivers in nearshore compared to offshore/oceanic ecosystems.

Human exploration and exploitation of the open ocean during the past few centuries ushered in a wave of globalization, commerce, and connectivity that dramatically increased human influence on offshore ecosystems through overfishing, pollution, climate change, and other processes (1–4). Although seen as a final frontier for commercial exploitation (5), people’s use and exploration of distant oceanic waters long predates modern industrialization, often by thousands of years (6). While human dispersal and capacities to interact with the open ocean have a deep history, it remains unclear when such early engagements shifted from movement across oceans and episodic offshore fishing to sustained, targeted extraction of biomass from oceanic ecosystems, and what ecological consequences may have followed. This stands in contrast to the well-documented ways people influenced nearshore coastal ecosystems for millennia (1, 7–12). Here, we integrate archaeological, historical, and fisheries data to investigate long-term interactions between people and offshore/oceanic ecosystems, specifically when, where, and how people began targeting pelagic fishes. We focus on tunas (Thunnini) and billfishes (Istiophoridae, Xiphiidae) because they are highly migratory oceanic predators at the top of pelagic foodwebs, support major fisheries today, and are sentinels of oceanic ecosystem health [*SI Appendix*, Table S1; (13)]. We use these taxa to investigate when people crossed a key threshold—from intermittent encounters with oceanic predators to sustained exploitation that made people a dominant ecological force in offshore/oceanic ecosystems. Archaeological research provides insight into the antiquity, evolution, technologies, and characteristics of past tuna, billfish, and other fisheries (14–16). These findings reveal a diverse history of engagement with large pelagic fishes, raising questions about where people were capturing pelagics, what technologies they used, and how extensive and sustained these fisheries were, especially compared to contemporaneous coastal fisheries (17).

Consistent terminology of the capture zones of tuna and billfish and accurate taxonomic attribution of archaeological and historical records of pelagic fishes [tunas (Thunnini) from other scombrids] are essential (17). We primarily rely on three terms: *coastal* for nearshore areas and coastal waters toward the continental shelf, *offshore* for deep (~200 m) water regions around the continental shelf, and *oceanic* for the open ocean well beyond the continental shelf (Fig. 1). These definitions address human access rather than fish biology, as tunas and billfishes are pelagic and mobile predators that venture into multiple habitats (18) yet represent biomass from oceanic habitats regardless of capture location. This framing distinguishes between i) coastal access to oceanic predators—potentially enabled by steep bathymetry, submarine canyons, deep channels, or episodic oceanographic events—and ii) sustained exploitation requiring technologically enabled and consistent access to offshore/oceanic habitats. We use this distinction to evaluate whether archaeological pelagic fish remains reflect access-mediated coastal encounters or routine offshore fishing.

**Fig. 1.**
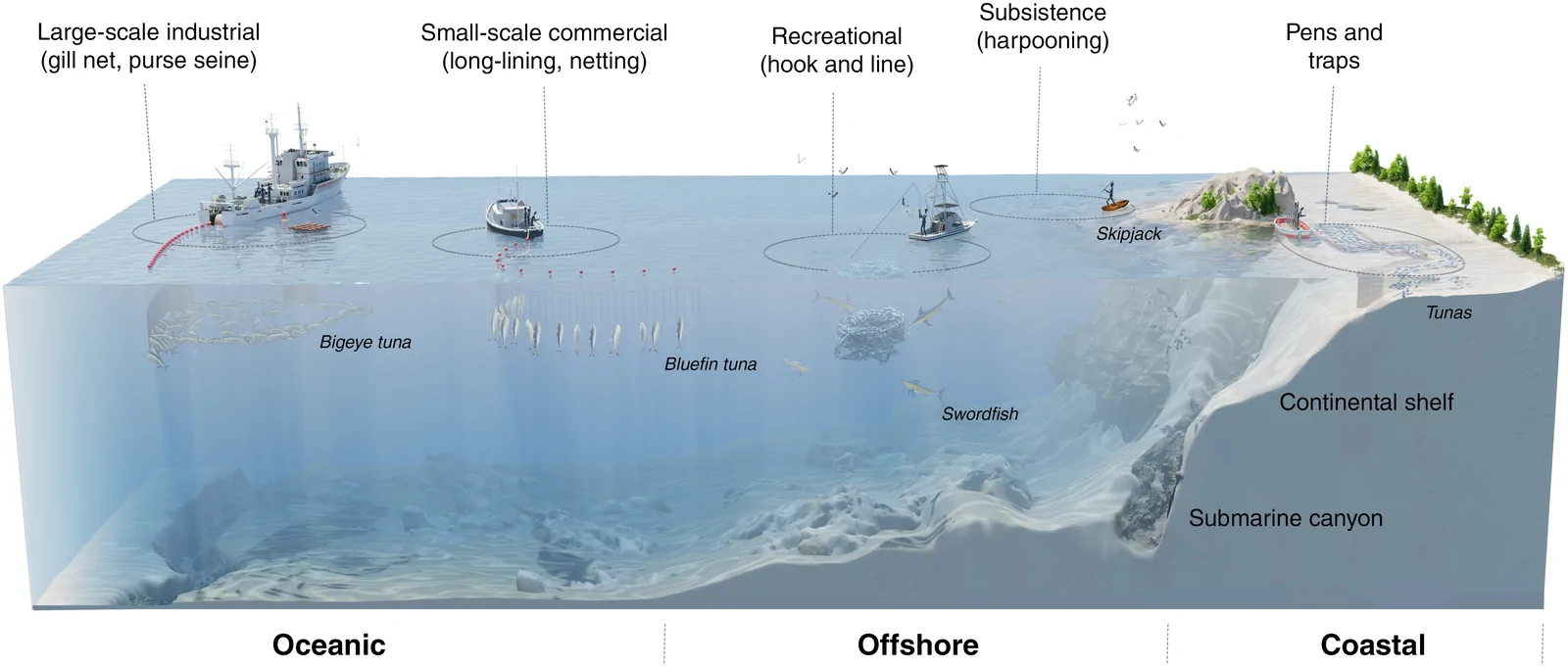
Habitats, technologies, and fisheries discussed in the text, including coastal, offshore, and oceanic zones and features where people captured tunas and billfishes through time (illustrated by Matt Harris). Early pelagic exploitation often occurred via coastal access pathways (e.g., steep bathymetry/submarine canyons), beginning as early as ~44,000 years ago in Island Southeast Asia and during the Holocene elsewhere; routine offshore and oceanic exploitation expanded mainly in the past ~200 y with industrial technologies.

Understanding when people began to access and extract biomass from offshore/oceanic ecosystems is particularly relevant today since tunas and billfishes support major commercial fisheries worldwide, including some of the world’s largest industrial fisheries. These species are managed by five Regional Fisheries Management Organizations (RFMOs) aligned with major ocean basins. Around 6,000,000 tons of harvested billfishes, tunas, and sharks are reported to RFMOs each year, >80 percent of which are oceanic tunas [*Thunnus* spp., *Katsuwonus pelamis,* etc., *SI Appendix*, Table S1; (19)]. These fishes are captured today in coastal regions, but more often in international, oceanic waters, using pole and line, trolling, long lining, purse-seines, gill nets, and traps (19). Interpretations of the status of oceanic tunas and billfishes have varied, with some arguing for severe global depletion of large predatory fishes relative to preindustrial baselines (20) and subsequent assessments showing a ~50% global average reduction in adult tuna and billfish biomass (21). More recent global syntheses demonstrate that some previously overfished tunas and billfishes have measurably been rebuilt, although outcomes remain uneven across species and regions and many populations remain below historical abundance (13).

Against this backdrop, we provide the first millennial-scale global synthesis of tuna and billfish exploitation, guided by three questions: 1) When did billfish and tuna fisheries begin and how did they change through time? 2) What were the cultural context and drivers of these fisheries? 3) When, where, and why did people start to pursue tunas and billfishes from oceanic environments? Our core hypothesis is that early encounters with oceanic predators were constrained by access and technology—often enabled by coastal “shortcuts” to deep water—whereas sustained exploitation expanded once technological innovations made offshore fishing reliable and scalable. We synthesize archaeological evidence from 11 regions across four ocean basins, providing global context for evaluating contemporary offshore fisheries and ecosystems and charting a course for future research (Fig. 2 and *SI Appendix*).

**Fig. 2.**
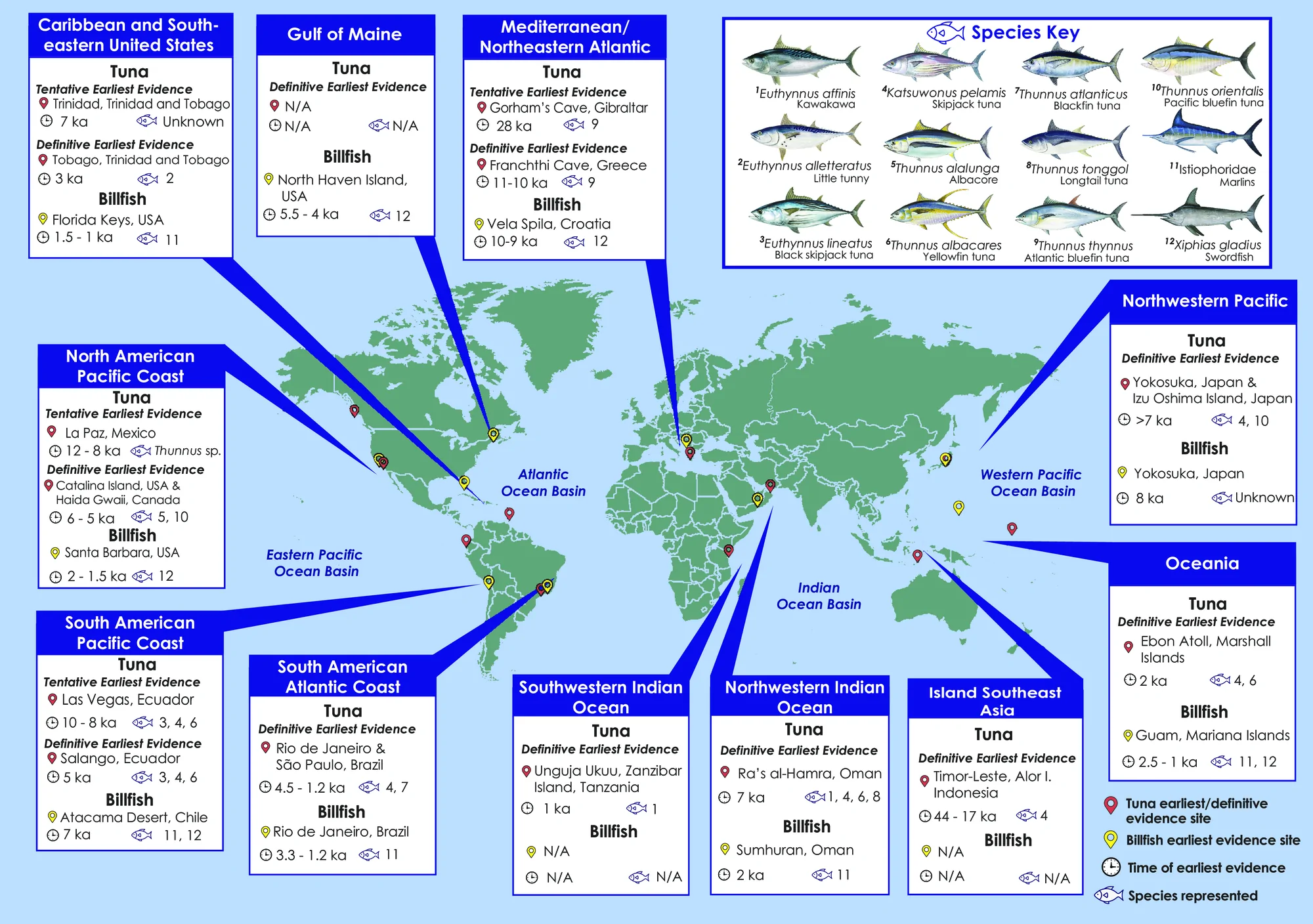
Earliest evidence for tuna/billfish harvest across 11 regions and four ocean basins, confirmed and tentative (*SI Appendix*, Table S2). Fishes listed are those at early localities, not necessarily all species identified. If none are listed for a particular category, they have not been documented archaeologically.

## Origins of Tuna and Billfish Fisheries

### Indian Ocean.

The Indian Ocean Basin is home to major tuna and billfish fisheries today and has archaeological evidence of millennia of coastal fishing, especially on the Arabian Sea and southeastern Africa. Numerous human settlements existed along the southeastern Arabian Coast beginning about 8,000-years ago (ya) (22–24). Sites from ~6,500 to 4,400-ya contain the remains of tunas alongside other species, including Ra’s al-Hamra 5 and Ra’s al-Hadd 6 where 51.3% and 61.9% of the fish assemblages (by NISP), respectively are from Thunnini (*Auxis thazard*, *Euthynnus affinis*, *Katsuwonus pelamis*, *Thunnus tonggol*, and *T. albacares*), though coastal *E. affinis* accounts for 91.7% and 75% of the total catch (25, 26). Archaeological evidence for billfish in this region generally occurs later in time around 2,000 to 1,000-ya (22, 27). Distinctive oceanographic conditions, including adjacent deep waters and strong seasonal monsoon-driven upwelling enriches regional surface waters with nutrients, supporting a diverse marine ecosystem that includes many schooling pelagic fishes, like *Sardinella* spp. and their predators. This likely facilitated coastal encounters between tuna and people without requiring sustained oceanic reach.

In southeastern Africa, archaeological evidence from ~50 sites in Kenya, Tanzania, Mozambique, Comoros Islands, and Madagascar indicate people were fishing for at least the past 2,000 y, targeting over 40 different fish families from a range of habitats and using diverse technologies (28). There is no evidence of regional billfish fishing, but by ~1,500-ya people began to capture small quantities of *E. affinis*. Despite significant knowledge of the ocean and sustainable practices for centuries, tuna fishing was limited, including no evidence for fishing for the larger, more oceanic species (28).

### Western Pacific Ocean.

Three primary areas of the western Pacific Ocean Basin contain archaeological evidence for tuna and/or billfish fisheries, including Island Southeast Asia (interface of Indian/Pacific oceans), Oceania, and Japan, Korea, and China. The earliest evidence for tuna fishing in the world dates to ~44,000-ya from Asitau Kuru rockshelter on eastern Timor-Leste in the Coral Triangle (14, 29, 30). Despite difficulty in taxonomic identifications, tunas (*Thunnus* spp.) and *K. pelamis* were recovered in the Pleistocene assemblage (31). Scombridae dominate in recently excavated Pleistocene/Early Holocene (NISP = 889) layers, but Carangidae dominate in the 3,800-ya and more recent phases (NISP = 102) (14, 31). The steep offshore profile and marine canyons along this section of Timor places deepwater (>100 m depth) habitats relatively close to shore, facilitating access to pelagic fishes (29, 31). Hook and line fishing and netting, including trolling from small boats could have been used to capture tunas, with archaeological fish specimens estimated to be similar sizes as those caught today [length = 40 to 100 cm; (31)]. Other regional archaeological fish assemblages are generally dominated by coastal species, potentially because they were not located near submarine canyons (32).

Oceania (Australia, Polynesia, Micronesia, etc.) is a vast area of some 50,000,000 km^2^, with rich traditions of fishing dating back to the Late Pleistocene and greatly expanding during the Late Holocene (33). Across this area, there is geographic variability in the fishes that were captured, but coastal species are most common (7, 33–35). Tunas and billfishes were captured in parts of Oceania but are patchily distributed in archaeological assemblages. Like Island Southeast Asia, tunas are sometimes identified only as Scombridae, including a few bones in the Solomon Islands and New Ireland dated in layers ranging from 42,000 to 5,000-ya, with most from younger deposits (33, 36). In Micronesia, small amounts of *K. pelamis* and *T. albacares* appear ~2,000-ya, while other scombrid remains, including *T. alalunga*, increase during the past 1,000 y in Polynesia and Micronesia (35, 37–41). Tunas may have been captured nearshore with hook and line and in deeper waters through trolling from canoes (38, 40, 42, 43). Evidence for billfish fishing is limited, largely confined to the Mariana Islands during the Late Holocene (44).

Japan has a rich tradition of fishing for tunas and billfishes. The earliest evidence for tuna (*Thunnus* spp. and *K. pelamis*) in Japan dates to 12,500 to 7,000-ya, though salmon (*Oncorhynchus* spp.) and other coastal fishes were far more common (45–47). Billfish remains occur at a number of sites as early as 8,000-ya (48). The arrival of cereal agriculture ~3,000 to 2,250-ya caused a decline in the overall reliance on fishing, but tuna continued to be an important food (49, 50). At two sites occupied ~800 to 600-ya, *X. gladius*, sharks, and dolphins occur and were likely harpooned (50). There is evidence of salting and drying tunas for trade by the 16th-century, with some records indicating capture of 10,000 “tuna” per day (51). Tuna continued to increase in importance during the 18th-century, underpinned by the adoption of tuna traps, a trend that continues today (52, 53). Archaeological *Thunnus* spp. and *X. gladius* have been identified in Korea possibly as early as 10,000 to 7,000-ya (54, 55). In China, a few tuna remains have been reported at 7,000 to 5,300-ya, but quantified data are limited (56). The dearth of tuna in Chinese archaeological sites is supported by the lack of descriptions of tuna in regional texts (57). Although much of the Chinese catch today is exported (58), there are some signs that domestic consumption is increasing, especially in large urban centers.

### Eastern Pacific Ocean.

From Alaska to Baja California, Indigenous peoples captured fish for >10,000 y using a wide variety of technologies, including hook and line, spears, harpoons, nets, traps, and watercraft (8, 59). Holocene fisheries generally concentrated on coastal species (8, 59, 60). Tunas and billfishes were captured by Indigenous peoples in the region, but in relatively circumscribed areas and after ~5,000-ya (61–63). On Vancouver Island and Haida Gwaii there was a specialized and intermittent fishery for *T. orientalis* beginning ~5,000-ya where people periodically fished for tunas during warm summer periods (61). In southern California, people captured small amounts of *T. alalunga*, *T. albacares*, *T. orientalis*, and *K. pelamis* primarily after 5,000 to 2,000-ya (63). Santa Catalina Island is a notable exception where tuna comprise ≥20% of some assemblages (62). Evidence for tuna fishing is scarce in Baja California, with clear evidence at only one archaeological site (63). There was a well-established fishery for swordfish and occasionally marlin beginning in southern California ~2,000-ya (64). Tuna and billfish fisheries in southern California were closely linked to submarine canyons and steep bathymetry that bring deepwater fishes close to shore, and through island-mainland trade/interaction, which put people in deepwater habitats (63).

Along the south Pacific from Ecuador to Chile, trans-Holocene fisheries are generally dominated by coastal fishes (65, 66). Billfish and tuna also occur, especially at sites Salango and Zapatero (15, 67, 68). Near the equator, Salango has a rich fish assemblage, including *T. albacares*, *K. pelamis*, *T. lineatus*, and *A. thazard*, which comprise 40% of the assemblage at 5,600 to 3,500-ya and 80% at 1,200 to 400-ya (67). South of Ecuador, archaeological tunas are rare, but Zapatero, Chile contains abundant billfish remains, and trace amounts of *K. pelamis* and *Thunnus* sp. (15). People were harpooning billfish (*X. gladius*, *Kajikia audax*) by ~7,000 to 6,000-ya at Zapatero, making it among the earliest billfish fisheries in the Americas (15). Chilean sites with billfish were located near relatively steep offshore bathymetry indicating these fishes were likely obtained coastally. Although more limited than the Ecuadorian and Chilean fisheries, people also captured *E. lineatus* and *K. pelamis* ~2,000 to 500-ya on the Panamanian Pacific Coast, further demonstrating the long-term regional interactions between people and tunas (69).

### Atlantic Ocean.

The Atlantic Ocean Basin contains several regions where tuna and billfish were caught in the deep past, with considerable variation in scale and intensity. In the western Atlantic, the Brazilian Coast contains some 10,000 y of human occupation and well-studied archaeological fish assemblages (12, 70–72). Coastal fishes dominate most assemblages, with tuna and billfish present at low frequency (12, 70, 71). A few southeastern Brazil shell mounds contain small amounts of *Istiophorus albicans*, *K. pelamis*, and likely *Thunnus atlanticus* at ~4,500 and ~1,500-ya (70, 71). Other parts of Brazil have no known tuna or billfish remains, suggesting these fish were an incidental or minor catch, perhaps in areas with warmer waters (12). The dominance of coastal taxa and scarcity of large pelagics are consistent with broad continental shelf settings and limited nearshore bathymetric “shortcuts” to deep water.

The Caribbean Islands have a ~6,000-y history of human settlement and robust archaeological evidence for fishing from ~2,500 to 500-ya (73). Nearshore fishes were captured across Caribbean Islands, especially from coral reefs, mangroves, and estuaries (9, 74–76). *Thunnus* sp., *K. pelamis*, *E. alletteratus*, *Scomberomorus* sp., and *Auxis* sp. have been identified in at least 31 sites from 20 islands. Tunas were likely captured nearshore using hook and line or spearing from canoes (75, 77, 78). Despite their presence in ~1,300 to 300-ya sites, tunas were not a significant focus of regional fisheries, present in small amounts compared to coastal species. Tunas occur most frequently in the southeastern Caribbean (Grenadines, Trinidad, and Tobago) (77, 78), perhaps due to proximity to seasonal tuna spawning grounds (79).

The Gulf of Mexico and southeastern Atlantic Coast of the United States have a 14,500-y history of human occupation and evidence for fishing by 6,000-ya following the stabilization of post-glacial sea level rise (80). Despite this region boasting important tuna and billfish sport and commercial fisheries today, there is no evidence for sustained Indigenous harvest of tunas and billfishes with only trace amounts documented in the archaeological record (81, 82). This may be due to the presence of rich estuarine and other coastal habitats that were a focus of people’s subsistence (83–85). Limited offshore fishing in the southeastern USA began after the arrival of European colonists and African slaves during the 16th–17th-centuries but did not develop further until the 19th-century (86).

In the Gulf of Maine, no tuna remains have been identified, but there was a culturally significant *X. gladius* fishery between ~5,500 to 3,700-ya (87, 88). During this time, the region likely had a microtidal environment with warm surface water that attracted swordfish during summer, abruptly ending ~3,500-ya due to increased tidal amplitude and marine cooling (89). Between ~4,200 to 3,750-ya, eight central Maine archaeological sites, two along the southwestern Gulf of Maine, and another in New Hampshire contain swordfish remains (88). People in the region were harvesting a range of coastal fish using hook and line and other technologies, while the specialized swordfish fishery used ground slate and moose bone dagger harpoon technology, likely deployed from canoes in coastal areas (88).

The northeastern Atlantic and Mediterranean were among the most important places in the world for early tuna fishing and the commercialization of offshore fisheries (16). *Thunnus thynnus* bones occur at Gorham’s Cave, Gibraltar at 28,000 to 24,000-ya, likely from scavenging due to the small amount of tuna bone and absence at other early sites, but after ~13,000 to 7,800-ya systematic fishing increases significantly (16, 90). Some 5,000 *T. thynnus* vertebrae have been identified at Franchthi in Greece ~11,000 to 9,600-ya, with small amounts of *X. gladius* in 10,000-9,000-ya deposits at Vela Spila, Croatia (91, 92). Significant amounts of *E. alletteratus* also occur in Greek sites by 6,300-ya or earlier and are initially more numerous than *T. thynnus* remains at some sites (93, 94). The number of sites reporting tuna remains expands rapidly in the Mediterranean and to Scandinavia ~8,800 to 5,500-ya (16, 95, 96). Tuna fishing relied on hook and line, harpoons, traps/nets, and other techniques in varying degrees (94, 97–99). These early fishers probably made use of the coastal topography common near these sites, including shallow embayments, narrow underwater canyons, or other areas close to shore where fish could have migrated or been driven closer to shore (16, 94, 96). By ~3,000-ya, *T. thynnus* fishing increased tremendously, including large-scale trade, early commercialization, and new specialist sites and nets/traps (16). By the 16th-century, Spanish fish traps may have caught ~14,000 tons of *T. thynnus*, rivaling fishing effort in the modern era. During this time, the fishery also expanded further from shore in Belgium, Scandinavia, and East Greenland (16). A distinctive combination of predictable migrations, concentrated coastal chokepoints, and fixed-gear technologies (e.g., traps) created a pathway to sustained regional exploitation without requiring routine offshore reach.

Archaeological and historical remains of northeastern Atlantic and Mediterranean *T. thynnus* have been analyzed via isotopic and genetic methods, enhancing our understanding of tuna historical ecology (100–102). Measurable impacts of the fishery, including reductions in *T. thynnus* stocks, occurred primarily during the industrial era (101, 102). However, isotopic data paint a more complicated picture, indicating people shifted away from coastal fishing by ~500-ya, perhaps due to degradation of coastal fisheries and habitats (100).

## Discussion

### Antiquity and Change Through Time.

People fished for tunas and billfish for millennia, usually alongside more intensive fishing of coastal species. Tunas (Thunnini) were identified in archaeological sites from all ocean basins and subregions in our analysis, except the Gulf of Maine. The oldest evidence for tuna fishing dates to ~44,000-ya at East Timor (14, 30, 31). These remains are difficult to identify to species, but likely include *K. pelamis* harvested from deepwater coastal channels (31). Other Late Pleistocene evidence comes from Mediterranean *T. thynnus* bones at ~26,000-ya, but these were likely scavenged by Neanderthals (16, 17). The Mediterranean contains a few other sites dated to the Pleistocene–Holocene transition, but after 10,000 to 8,000-ya sites with tunas begin to increase in the Mediterranean and Atlantic Europe (16, 94). By ~6,000 to 5,000-ya, tunas occur in coastal archaeological sites in British Columbia, California, and Chile (15, 63), and in the Late Holocene, tuna remains occur in at least trace amounts around the world, including several species in Caribbean Island sites (9, 78, 103), and in all ocean basins described here. In several areas, including Australia, Aotearoa, Brazil, southwestern Indian Ocean, and the southeastern United States, the archaeological record suggests there was minimal to no tuna harvest until the industrial period.

Billfish remains are more patchily distributed than tunas in the archaeological record and to our knowledge there are no archaeological billfish remains prior to the onset of the Holocene. Some of the earliest evidence is from a small number of *X. gladius* remains identified in Croatia at ~10,000 to 9,000-ya (92). Billfish were identified in Japan ~8,000-ya (48) and by ~6,000-ya abundant *X. gladius* and *K. audax* remains appear in Chile (15). At roughly this same time, a specialized fishery for swordfish formed in the Gulf of Maine, which abruptly ceased ~3,700-ya (87, 104). People were fishing for Istiophoridae in Taiwan by 4,000-ya and for *X. gladius* and *K. audax* in southern coastal California by 2,000-ya (64, 105). Billfish remains also occur in the Late Holocene Marianas Islands and Philippines, but these are limited, despite robust fisheries for other species (106, 107).

Where were people deep in the past fishing and obtaining these pelagic species? Oceanographic, climatic, and other conditions (e.g., postglacial sea level rise) vary significantly through time and pose challenges to answering this question (17). Changing ocean conditions influenced human access to tunas, billfishes, and other marine foods. However, for much of the time period (Holocene) discussed here global sea levels were similar to today (especially since 6,000 to 5,000-ya), and despite climatic events such as ENSO and the Little Ice Age (7, 38, 64), oceanic conditions were also largely comparable to today.

The question of where people were obtaining tunas and billfishes has been heavily debated, with some arguing that fishing for Thunnini, billfish, and other oceanic predators is generally a Holocene phenomenon, but these propositions had never been tested at a global scale (14, 17, 31). Our findings indicate that billfish and tuna fishing was rare but not absent until the Middle/Late Holocene. Furthermore, oceanic predators were generally fished in considerably less abundance than coastal fishes, a pattern influenced by the comparatively easy access to coastal and terrestrial resources. However, this portrayal masks incredible variability in the archaeological record and minimizes the capabilities of Indigenous fishers. Regions with strong evidence for early tuna and billfish fisheries share bathymetric and oceanographic features that bring deep pelagic habitats and large ocean predators into proximity with the coast, including submarine canyons, steep continental margins, or deep-water channels (Island Southeast Asia, southern California, Chile, etc.), and/or regional environmental conditions that episodically enhanced coastal access to some species (Gulf of Maine and Pacific Northwest). In contrast, coastlines with broad, shallow continental shelves, and limited nearshore bathymetric relief (e.g., Brazil and southeastern United States) show a paucity of archaeological tuna and billfish remains. Hence, the utilization of pelagic resources was shaped by predictable coastal access mechanisms and opportunities rather than the capability of Indigenous fishers.

### Culture, Technology, and Economy.

The development or refinement of specialized technologies (seaworthy boats, harpoons, strong line, and other tackle) was essential for harvesting tunas and billfishes, and other large marine animals. Harpooning and the use of boats are common across regions, and tunas could have been caught using nets, spears, and/or fishhooks in the deeper past. Ethnographic accounts help expand on these assertions. In the Pacific Northwest, *T. orientalis* fishing occurred during summer warm water events in deepwater fjords at night, with spotters on sea cliffs alerting boats to the presence of tuna at the surface (61). Fires in the boats were used to attract tunas when bioluminescence events would help visibility, and tunas were subsequently harpooned. In Island southeast Asia today, fast moving, coastal deepwater channels are targeted by people in boats to obtain skipjack and other scombrids using hook and line, harpoons, and nets—strategies likely used in the past (31). In the Mediterranean, paintings, written records, and archaeological evidence illustrate a wide range of techniques used to capture *T. thynnus* prior to industrialization. These include fish traps, pens, nets, harpoons, and hook and line from watercraft and shore (16, 94). The wide range of global technologies used to capture large ocean predators were outgrowths of those used for catching coastal fishes by Indigenous people for centuries or millennia. In many regions, these technologies scaled pelagic capture without necessitating sustained travel into oceanic habitats.

Indigenous and local fisheries for large ocean fishes were often deeply linked to ceremony and ritual, including the billfish and tuna fisheries of the Americas and Caribbean (15, 64, 104, 108). In Chile and California, billfish are depicted in rock art, and in California and Maine swordfish are found in mortuary contexts (15, 64, 87). Oral tradition in Chumash territory in California indicates that people practiced a swordfish (‘elye’wun) dance, and swordfish were viewed as people of the sea that drove whales ashore (64). Tunas were part of ritualized fisheries in Oceania where *K. pelamis* in the Tuamotus ~1,400-ya were used as offerings at sacred sites, and *K. pelamis* trolling is sacred with songs about the practice documented elsewhere in Oceania (40, 42). In the Mediterranean, tunas were depicted in frescoes, on vessels, and other artforms that demonstrate their significance to society (16, 94). Tunas were among the few fish ritually killed to honor the gods in ancient Greece, and in some places the first tuna of the season was killed as an offering to Poseidon (94). In Japan, skipjack/bonito was a common offering in 8th-century courtly rituals (109). These examples demonstrate that billfish and tunas held significant religious functions cross-culturally around the world, perhaps related to the challenges in capturing them, their large size, and function as ocean predators. Fishing for tunas and billfishes was motivated by both ecological (access and habitat), social (ritual, ceremony, prestige, etc.), and economic factors that varied across space and through time (110).

### Emergence of Oceanic Exploitation.

Today, tuna and billfishes are important apex predators in the world’s oceanic ecosystems, with significant commercial and sport fisheries. Modern fisheries research, largely based on statistics and records compiled since the 1950s, shows that tuna and billfish are subject to high fishing mortality and intensive harvest globally (13, 19). IUCN Red List Index trajectories and fishery stock assessments document major declines in biomass of oceanic tunas, billfishes, and large sharks that intensified through the mid to late-20th-century, though many assessed populations later stabilized or improved where harvest controls and rebuilding measures were adopted (13). Modern records include **~**6,000,000 tons of billfishes, tunas, and sharks reported to RFMOs annually (19), and total reported tuna catch has increased ~15-fold from the 1950s to 2024, underscoring how rapidly extraction from oceanic ecosystems expanded once industrial technologies made access reliable and scalable (3).

**Fig. 3.**
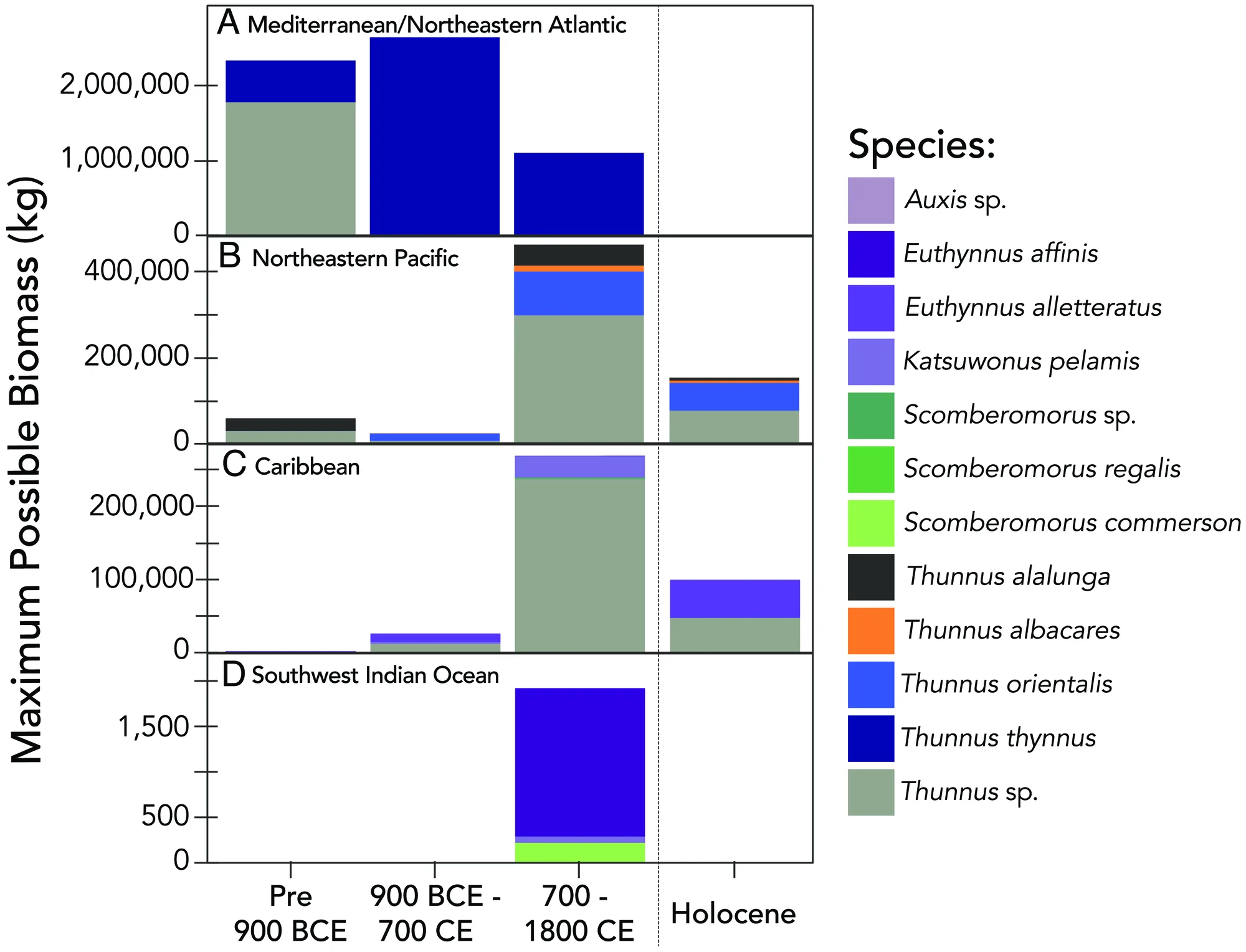
Upper bound tuna biomass represented in the archaeological record of four regions (*SI Appendix*, Tables S5 and S6 and SI-2). Data binned by time period plus a “Holocene” classification for sites with low chronological resolution (16, 63). Data represent (*A*) 115 sites, (*B*) 72 sites, (*C*) 31 sites, and (*D*) three sites. These biomass estimates represent a ceiling on what available archaeological data can reveal by assuming each identified tuna specimen represents the largest known individual of its taxon (*SI Appendix*, SI-2).

Despite being harvested globally since at least the Late Holocene, and in some regions since the terminal Pleistocene or earlier, preindustrial harvest of tunas and billfishes was generally light and/or rare, with these pelagic fishes taken primarily from coastal waters. To contextualize the magnitude of the early fisheries relative to modern exploitation, we estimated the upper bound tuna biomass represented in available archaeological faunal assemblages across four key regions (Fig. 3 and *SI Appendix*, SI-2). These data demonstrate that modern fisheries differ dramatically from the relatively modest Holocene archaeological record of oceanic tuna and billfish exploitation, factors that cannot be explained by the incomplete nature of the archaeological record alone. The northeastern Atlantic and Mediterranean are a potential exception, as commercial tuna fisheries began at ~2,500-ya in this region (16). However, exploitation initially occurred fairly close to shore, and archaeological, genetic, and isotopic evidence indicates that measurable impacts on the population likely did not occur until after widespread fishing developed offshore during the industrial period or slightly earlier (16, 100–102, 111). Based on our assumptions (e.g., each specimen represents an individual tuna of the maximum recorded size), our estimated upper bound tuna biomass removal for the Mediterranean/Northeastern Atlantic is ~6,000 tons across millennia. While the coarse nature of these estimates and the patchiness of the archaeological record preclude a direct comparison, modern global tuna harvests have exceeded 5,000,000 tons *annually* in recent decades (112).

Even where tuna and billfish fisheries developed early, sustained pressure on oceanic components of populations is a late development—consistent with the premise that routine offshore/oceanic reach, rather than occasional coastal access, is required for persistent, large-scale extraction. These results indicate that sustained offshore/oceanic exploitation of tunas and, likely, billfishes is largely a twentieth-century phenomenon. This pattern contrasts with the well-documented trans-Holocene (and earlier) record of human interactions with coastal marine ecosystems, from overharvest to sustainable stewardship (1, 9–11). This raises a fundamental question about human influence on offshore ecosystems: What does it mean to be impacted by people so dramatically in such a short time?

People have had long histories of harvesting and interacting with tunas and billfishes, spanning millennia. Yet, the open ocean remained largely free of major and, in several cases, any harvest until the past 200 y (4). Our synthesis demonstrates that this longer time period needs to be a focal point of future research into resilience and sustainability to better contextualize the mid-20th century “Great Acceleration” of fisheries (3). For example, how do human impacts in coastal habitats with records of intensive harvest spanning thousands of years compare to those in oceanic ecosystems with much later and more punctuated fisheries? This discontinuity also reframes how we interpret modern baselines: The period of sustained extraction from offshore ecosystems is comparatively brief, and the greatest expansion in capacity occurred within living memory. At the same time, recent syntheses suggest that where effective governance, monitoring, and enforceable harvest controls and rebuilding plans are in place, multiple tuna and billfish populations have stabilized—highlighting the potential to alter population trajectories of marine megafauna in the industrial era (13). These improvements, however, do not imply uniform sustainability across all fisheries or a return to preindustrial abundance; substantial stock-specific and regional challenges remain, and impacts on bycatch taxa (notably some billfishes and many oceanic sharks and rays) remain a major concern (113).

As we seek to improve our understanding of human impacts on ocean ecosystems and promote sustainable ocean health, evaluating the effects of people on marine ecosystems across long-time scales has a central role to play. Our analysis highlights key opportunities for interdisciplinary research by the global fisheries, ecological, historical, and archaeological communities. Such opportunities are exemplified by genetic and isotopic studies of *T. thynnus* in the Mediterranean and European Atlantic that illustrate the relatively recent impacts of people on these fishes and resultant changes to populations and ecosystems from intensive offshore fishing in the 20th-century (100–102, 114). A complementary research frontier is to reconstruct the structure of pelagic food webs before and after industrialization to evaluate how large removals of oceanic apex predators (including oceanic sharks) reorganized trophic pathways. Another research area is improving the resolution of taxonomic identifications of archaeological tuna/billfish remains and estimating past fish body size. Zooarchaeology by mass spectrometry (ZooMS) is proving important for species identification (39, 41) and allometric formulae help estimate past fish size (115). These multidisciplinary approaches focused on archaeological/historical tuna and billfish provide a significant opportunity to build on our synthesis and use the past to evaluate the present and future of oceanic ecosystems and the health of pelagic fisheries. Bridging archaeology, history, and fisheries science improves how we define management-relevant baselines for highly migratory predators thereby sharpening historical inference and contemporary management context and helping align conservation targets with realistic, time-explicit baselines.

## Supplementary Material

Appendix 01 (PDF)

## Data Availability

Study data are included in the article and/or *SI Appendix*.
