# Supplementary material for: Global origins of tuna and billfish fisheries reveal the transition from coastal access to oceanic exploitation
Source: Proc Natl Acad Sci U S A. 2026 Oct 16;123(43):e2512066123. doi: 10.1073/pnas.2512066123 (PMC13715807; doi:10.1073/pnas.2512066123)
Supplement: Supplementary file 1 [file pnas.2512066123.sapp.pdf]

Supplementary Appendix:

Global Origins of Tuna and Billfish Fisheries, T. C. Rick et al.

List of Supplementary Tables and Text

Table S1. Primary fish species included in this study.

Table S2. References for the data presented in Figure 2.

Table S3. Species considered from Japan.

Table S4. Early (ca. 5000-3500 BCE) and Middle (3500-2500 BCE) Jōmon tuna and skipjack remains from the Miyano shell midden (Iwate prefecture).

Table S5. Tuna NISP and associated upper bound biomass represented in the archaeological record of four key geographic regions.

Table S6. Sites with archaeological tuna remains across four key geographic regions (n = 221 sites total).

SI-1. Supplementary background text for regional sections.

SI-2. Estimating upper bound archaeological tuna biomass from excavated sites.

Table S1. Primary fish species included in this study. Listed taxa comprise the major commercially important tunas (tribe Thunnini, family Scombridae) and billfishes (family Istiophoridae and Xiphiidae) occurring globally. IUCN status is reported as Red List category. Taxonomy and common names follow Collette and Graves (1).

**Group**	**Family**	**Species**	**Common Name**	**IUCN Status (year of last assessment)**
Billfishes	ISTIOPHORIDAE	*Istiompax indica*	Black marlin	Data deficient (2021)
Billfishes	ISTIOPHORIDAE	*Istiophorus platypterus*	Sailfish	Vulnerable (2021)
Billfishes	ISTIOPHORIDAE	*Kajikia albida*	White marlin	Least concern (2021)
Billfishes	ISTIOPHORIDAE	*Kajikia audax*	Striped marlin	Least concern (2021)
Billfishes	ISTIOPHORIDAE	*Makaira nigricans*	Blue marlin	Vulnerable (2021)
Billfishes	ISTIOPHORIDAE	*Tetrapturus angustirostris*	Shortbill spearfish	Data deficient (2021)
Billfishes	ISTIOPHORIDAE	*Tetrapturus belone*	Mediterranean spearfish	Least concern (2021)
Billfishes	ISTIOPHORIDAE	*Tetrapturus georgii*	Roundscale spearfish	Data deficient (2021)
Billfishes	ISTIOPHORIDAE	*Tetrapturus pfluegeri*	Longbill spearfish	Least concern (2021)
Billfishes	XIPHIIDAE	*Xiphias gladius*	Swordfish	Near threatened (2021)
Tunas	SCOMBRIDAE	*Allothunnus fallai*	Slender tuna	Least concern (2022)
Tunas	SCOMBRIDAE	*Auxis rochei*	Bullet tuna	Least concern (2022)
Tunas	SCOMBRIDAE	*Auxis thazard*	Frigate tuna	Least concern (2022)
Tunas	SCOMBRIDAE	*Euthynnus affinis*	Kawakawa	Least concern (2022)
Tunas	SCOMBRIDAE	*Euthynnus alletteratus*	Little tunny	Least concern (2022)
Tunas	SCOMBRIDAE	*Euthynnus lineatus*	Black skipjack	Least concern (2022)
Tunas	SCOMBRIDAE	*Katsuwonus pelamis*	Skipjack tuna	Least concern (2021)
Tunas	SCOMBRIDAE	*Thunnus alalunga*	Albacore	Least concern (2021)
Tunas	SCOMBRIDAE	*Thunnus albacares*	Yellowfin tuna	Least concern (2021)
Tunas	SCOMBRIDAE	*Thunnus atlanticus*	Blackfin tuna	Least concern (2021)
Tunas	SCOMBRIDAE	*Thunnus maccoyii*	Southern bluefin tuna	Endangered (2021)
Tunas	SCOMBRIDAE	*Thunnus obesus*	Bigeye tuna	Vulnerable (2021)
Tunas	SCOMBRIDAE	*Thunnus orientalis*	Pacific bluefin tuna	Near threatened (2021)
Tunas	SCOMBRIDAE	*Thunnus thynnus*	Atlantic bluefin tuna	Least concern (2021)
Tunas	SCOMBRIDAE	*Thunnus tonggol*	Longtail tuna	Data deficient (2021)

Table S2. References for the data presented in Figure 2. Parentheticals denote bibliography numbers.

Region	Sources for Tuna	Sources for Billfish
Caribbean and Southeastern United States	(2, 3) Ali 2012; Steadman and Jones 2006	(4) Oliveira 2024
Gulf of Maine	(5) Spiess and Lewis 2001	n/a
Mediterranean/Northeastern Atlantic	(6) Andrews et al. 2022	(7) Burić and Težak Gregl 2015
Northwestern Pacific	(8) Imamura 1996	(9) Shibata et al. 2011
Oceania	(10) Lambrides and Weisler 2018	(11) Amesbury and Hunter-Anderson 2008
Island Southeast Asia	(12, 13) Boulanger et al. 2025; O'Connor et al. 2011	n/a
Northwestern Indian Ocean	(14, 15) Beech 2004; Uerpmann and Uerpmann 2005	(16) Carenti and Wilkens 2008
Southwestern Indian Ocean	(17) Prendergast et al. 2017	n/a
South American Atlantic Coast	(18, 19) Lopes et al. 2016; Mendes et al. 2018	(18, 19) Lopes et al. 2016; Mendes et al. 2018
South American Pacific Coast	(20, 21) Béarez et al. 2012; Béarez and Stahl 2020	(22) Béarez et al. 2016
North American Pacific Coast	(23) Rick et al. 2025	(24, 25) Bernard 2005; Davenport et al. 1993

SI-1. Supplementary background text and methods for regional sections

The following sections provide additional context and background on the nature of past fishing practices in each subregion, emphasizing tuna and billfish fishing through time. For some sections there are supplemental archaeological details and for others there is additional historical context. This information is intended to supply interested readers with additional information that can augment the syntheses provided in the main text, other references that can be further explored by researchers, and the ability to investigate a specific subregion more deeply. Different subregions rely on unique local/regional cultural chronologies. In the main paper and supplement, we maintain those chronologies and rely on BCE/CE conventions or years ago when referring to years before present. Our chronological assessments are based on available absolute and relative date associations for each subregion.

For our synthesis, we rely largely on occurrences of fish bones, teeth, otoliths, and other remains identified in the archaeological record by regional zooarchaeological specialists. In a few cases, we also present genetic and/or Zooarchaeology by Mass Spectrometry (ZooMS) results which are specifically noted in the text. With few exceptions, size estimates for archaeological tunas and billfishes are limited and often anecdotal (e.g., comparisons of an archaeological specimen next to a similarly sized modern specimen of known size), precluding quantitative comparisons of size changes through time and across regions (see (22, 26-28)). Each of the sections below also provides the background data and references used to create the information in Figure 2, with specific references for earliest occurrences called out in Table S2.

SI-1.1. *Southwestern Indian Ocean*. Fishing has long been an important economic and social activity in the Southwestern Indian Ocean, an area rich with marine life located south of the

equator between the African coastline to the west and multiple oceanic islands to the east. Historical and archaeological evidence document an active exchange of objects, people, and ideas across this region from at least the beginning of the first millennium CE. The data presented here include a review of approximately fifty archaeological sites located in Kenya, Tanzania, Mozambique, the Comoros, and Madagascar, that provide information about the quantities and types of fish that people caught, ate, and discarded (29). Although people were actively fishing in this region throughout the first and second millennium CE, there is no archaeological evidence of the exploitation of billfish. The earliest record of tuna (*Euthynnus affinis*) fishing occurs at the site of Unguja Ukuu, Zanzibar, and dates to the second half of the first millennium CE (17). Throughout the first half of the second millennium CE, people continued to target tuna species, particularly *E. affinis*, but there are only three additional sites where tuna/scombrids are present and were less than two percent of all the fish identified, including Kizimkazi Dimbani (30) with *E. affinis* remains and Mduuni (31) with only Scombridae identified. Similar results are present at Songo Mnara, including *E. affinis*, *K. pelamis*, and likely *Scomberomorus commerson* remains (Table S6). The small quantities of tuna bones (152 NISP) recorded at archaeological sites in the region indicate that tuna fishing existed on a minor scale and occurred primarily in the second millennium.

Archaeological evidence demonstrates that fishers in the Southwestern Indian Ocean had the tools and knowledge to manage productive nearshore fisheries for centuries, targeting over 40 different fish families primarily from coral reefs and estuaries (29). Early records at Kuumbi Cave on Zanzibar Island (32) and Juani Primary School on Mafia Island (33) indicate that from the Early Iron Age, around 400 CE, people regularly exploited a variety of nearshore fish such as emperors (Lethrinidae), groupers (Serranidae), parrotfish (Scaridae), and triggerfish (Balistidae).

While nearshore fisheries continued for hundreds of years, the second millennium saw an increasing exploitation of larger, predatory fish, including tunas and sharks. These shifting fishing strategies occurred alongside other changes in the 10th-13th centuries, such as the growth of coastal cities, an emerging elite, and the development of feasting practices (34). Overall, archaeological data from this region show that tuna fishing played a minor role in a fishery focused on nearshore coral and estuary habitats; tuna became increasingly important during the second millennium as elite residents gained control of key resources associated with maritime activities. Despite reviewing all available archaeological fish assemblages reported for the region, and evidence for greater tuna fishing in the Northern Indian Ocean (see below), occurrences of tunas were extremely limited as noted above. In south Africa, a few bones from another scombrid (*Scomber japonicus*) were identified in Middle and Late Stone Age sites (35). The dearth of tuna in southern and western Africa is a real pattern, but we caution that there are limited samples to draw from and more research on the archaeology of fishing in these areas is an important topic for future investigation.

Today, tuna species (Thunnini), including those known archaeologically: *Euthynnus affinis* and *Katsuwonus pelamis,* as well as closely related *Scomberomorus commerson*, are among the top ten pelagic species caught in the Indian Ocean, with an average catch of just under 1.2 million metric tons per year (36). Catch records document the modern history of the tuna fishery, marked by the 1950s arrival of foreign longline fleets in the Indian Ocean that spurred industrial scale fishing. Research on fishing practices in the Southwestern Indian Ocean during the colonial period would help bridge the gap between the archaeological and modern data sets.

SI-1.2. ***Northern Indian Ocean*.** Since at least the time of Alexander the Great's military campaigns in Asia, it has been known that the inhabitants of the Arabian Sea coast lived primarily from fishing and were referred to as the *ichthyophagians* (37). These "fish-eaters" were culturally distinct due to the central role of fishing in their subsistence and their exceptionally high consumption of marine resources.

Archaeological evidence indicates that numerous human settlements existed along the southeastern Arabian coast beginning in the 6th millennium BCE (14, 38, 39). The easternmost corner of the Arabian Peninsula—today part of the Sultanate of Oman—has yielded the strongest evidence for specialized pelagic fishing. From Muscat to Ra's al-Jinz, several Neolithic sites have produced substantial quantities of large pelagic fishes. At Ra's al-Hamra 5, Thunnini (*Auxis thazard*, *Euthynnus affinis*, *Katsuwonus pelamis*, *Thunnus tonggol*, and *Thunnus albacares*) account for 51.3% of a total NISP of 12,891 (15). However, the assemblage is heavily dominated by the mostly coastal species, kawakawa (*E. affinis*), which represents 91.7% of the catch. At the Ra's al-Hadd 6 site, dated to the 3rd millennium BCE, Thunnini constitute an even larger proportion—61.9% of a total NISP of 16,228 (40). Kawakawa again dominates at 75%, followed by yellowfin tuna at 13.5%. At Ra's al-Jinz 2, an Early Bronze Age settlement regarded as a specialized fishing site, the primary focus was once more on large pelagic species such as yellowfin tuna (41). Records for billfish in archaeological sites generally occur later in time around 2000-1000 years ago in this area (14, 16).

This region is characterized by distinctive oceanographic conditions, including adjacent deep waters and strong upwelling activity. Seasonal monsoon-driven upwelling enriches surface waters with nutrients, supporting a diverse marine ecosystem that includes many schooling pelagic fishes, such as sardinellas (*Sardinella* spp.), and their predators. True tunas remain

among the most important fisheries resources in Oman today, second only to small pelagic species (42).

SI-1.3. *Island Southeast Asia.* The earliest archaeological evidence for tuna fishing globally dates back to ~44,000 years ago in the rockshelter known as Asitau Kuru (previously Jerimalai), at the easternmost tip of Timor-Leste, Island Southeast Asia. This region is within the Coral Triangle marine biodiversity hotspot—a total of 432 fish species were recorded during a brief biological survey in 2008 with an estimated fish diversity in the order of 840 species (43). Asitau Kuru was first excavated in 2005 and again in 2017 and produced a cultural assemblage containing stone, bone and shell artifacts as well as abundant remains of marine fauna including turtle, fish, shellfish, crab, and urchin (44, 45). Today the shelter is less than 1 km from the coastline and ~70 m above mean sea level and is an area of steep offshore bathymetry such that Asitau Kuru would never have been much further from the coast than it is today. The steep offshore profile along this section of the north coast of Timor, including marine gorges puts deepwater (> 100 m depth) habitats relatively close to shore. Pelagic species are reportedly found in abundance today (43, 46). Local fishermen interviewed over a three-day period in June 2019 report catching *Thunnus albacares* (yellowfin tuna) during the dry season, *Katsuwonus pelamis* (skipjack tuna) most abundantly right after the rainy season, while *Auxis thazard* (frigate tuna) and *Gymnosarda unicolor* (dogtooth tuna) can be caught at any time in the year (46).

Fish bones at Asitau Kuru were difficult to identify due to poor preservation, but tuna (*Thunnus* spp.) and skipjack (*Katsuwonus pelamis)* were identified in the Pleistocene assemblage from the 2017 excavation (46). Scombridae (mackerels, tunas, bonitos) account for approximately 16% of total fish MNI from the 2005 excavations. In the 2017 assemblage

scombrids are the dominant taxon in both the Pleistocene and Early Holocene (NISP = 889), but Carangidae (jacks, pompanos, jack mackerels, runners, scads) are dominant in the Neolithic phase (NISP = 102) (13, 46). It is uncertain how people captured the tuna and other fish, but shore fishing and the use of boats is likely and probably became more prominent in the Terminal Pleistocene (47). The first fish hooks, shell single-piece circular rotating and jabbing hooks, appear ~17,000 years ago (47). Both fiber line fishing and netting could also have been used in the capture of Scombridae. The size of individual tunas (~40 to 100 cm) in Asitau Kuru is similar to what the local fishermen catch today trolling from small boats close to shore in the island's deep and fast flowing channel (46).

The steep bathymetry and offshore marine environment near Asitau Kuru provided its occupants with unprecedented access to pelagic species. In sharp contrast the other early archaeological sites in Timor and islands in Wallacea more broadly, contain mostly small inshore/reef species (48). Here Sorot Entapa on Kisar island is a coastally located shelter site where occupation dates from ~16,000. The large assemblage is comprised almost exclusively of inshore/reef fish of small size (48). Makpan on Alor Island dating from~ 40,000 years ago has little fish in the initial occupation phase, but fish bone increases significantly in the terminal Pleistocene/Early Holocene coinciding with the appearance of fish hooks. The Makpan assemblage was predominantly comprised of reef/inshore taxa (49). Another sizeable fish assemblage (27,441) was recovered from Tron Bon Lei, a shelter on the southwest coast of Alor where occupation begins ~21,000 years ago (50, 51). The Tron Bon Lei assemblage is mostly made up of reef/inshore families, but open water pelagic fish, including Scombridae, increase in number during the terminal Pleistocene (50, 51). Here fish hooks placed as grave goods on a

female burial dating to ~11,500 years ago indicate the importance of fishing in both the everyday and spiritual realm, and a role for women in this activity (52).

SI-1.4. *Oceania.* Oceania – encompassing Australia, Melanesia, Polynesia and Micronesia – is a vast region of some 50 million km^2, and while today it is still characterized by significant cultural and environmental variability, the region is unified by the prominent role the ocean and coasts have played in its history. The earliest evidence for engagement with the coastal and marine landscapes of Oceania dates to 48,000 years ago in northwestern Australia at Boodie Cave [Barrow Island; (53)], and in the Pacific, 42,000 years ago at Buang Merabak [New Ireland, Papua New Guinea; (54, 55)]. At these two sites, the dates are associated with the presence of low abundances of marine 'dietary' fauna, primarily mollusk remains and unidentified fish bones.

The Australian Late Pleistocene—Early Holocene (~12,000 years ago) coastal record provides some more robust evidence of Indigenous fisheries [e.g. northwestern Australia; (56, 57)] but the richest fish bone assemblages are associated with the frequently called emergence of maritime specialist economies from ~3000 years ago (58-61). In the Pacific, there are earlier indications of focused fishing efforts at Kilu Cave (Solomon Islands) from 29,000 years ago (62). The most comprehensive records of marine fisheries are associated with the later settlement of Remote Oceania – Micronesia, Polynesia, and the island groups of Vanuatu, New Caledonia, and Fiji – from ~3500 years ago (63, 64). A diverse range of fish capture technologies were utilized across Oceania in the past, such as stone- and coral-walled traps and weirs, basket traps, dip nets, staked nets, spears, poisons, hook/gorge and lines, bow and arrows, harpoons, and oceangoing watercraft, including outrigger and bark canoes (65-69). Across Oceania, fishing

efforts primarily targeted inshore habitats, such as seagrass beds, intertidal reef flats, rocky reef cavities/ledges, sand bottomed bays or lagoons, and estuaries (56, 66, 67, 70-72). There is significant regional variation in the range of inshore species targeted during the Late Holocene, but commonly captured taxa from the tropical zone of Australia and the Pacific include parrotfish and wrasse (Labridae), emperors (Lethrinidae), surgeonfishes (Acanthuridae), snappers (Lutjanidae), goatfishes (Mullidae), and groupers (Epinephelidae), from sub-tropical central Australia, whiting (Sillaginidae), yellowfin bream (*Acanthopagrus australis*), mullets (Mugilidae), and catfish (Ariidae), and for temperate New Zealand (Aotearoa), we see silver sea bream (Sparidae), blue cod (*Parapercis colias*), and barracouta (Gempylidae) (66, 67, 72-74).

There are archaeological capture records of tunas, billfishes, and other large oceanic/offshore fishes, but these are geographically patchy and temporally variable across Oceania. For example, in Australia only one scombrid bone dated to ~1500-1000 years ago has been recovered archaeologically from eastern Torres Strait (75), but it is unclear whether this is evidence for tuna fishing, and then from Aotearoa there is no evidence for tuna capture, with all recovered scombrid remains believed to be blue mackerel (66, 76). Yet, today, in both Australia and Aotearoa tunas and billfishes are available and widely fished commercially and recreationally. The earliest potential evidence for tuna fishing comes from Melanesia, where a 'Thunnidae/Katsuwonid' vertebra was recovered from a layer dated to 42,000-29,000 years ago at the Buang Merabak site [New Ireland; (54)]. Also, from Kilu Cave (Solomon Islands), Scombridae remains (NISP = 101) were recovered from Layer II (~29,000 -20,000 years ago) and Layer I (~10,000 -5000 years ago), but these accounted for only 6% of the total identified fish bone assemblage, which was largely dominated by inshore coral reef species (62).

Taxonomic resolution, specifically the dominance of family-level identifications, have hindered our understanding of the antiquity, development, and species-composition of these potential oceanic/offshore tuna fisheries (10). Across Oceania, scombrid remains contribute approximately 10-25% (MNI values) of the catch at only eight sites, and they are spread across Melanesia (Motupore, Papua New Guinea), Micronesia (Fais Island [Caroline Islands], Ebon Atoll [Marshall Islands]), and Polynesia (Atafu Atoll [Tokelau], Hane, Ua Huka, Te Anapua, Ua Pou and Hanamiai, Tahuata [Marquesas Islands], Vaitoʻotia/Faʻahia complex, and Huahine [Society Islands]) (10, 74, 77-83). In the Pacific, scombrids generally represent less than 5%, and frequently less than 1% of the identified fish catch (10, 67, 79, 84).

There are confirmed tuna capture records, especially skipjack and yellowfin, from Micronesia dating from 2000 years ago, but the most detailed evidence for skipjack, yellowfin, mackerel, and albacore fishing are from Polynesia and Micronesia and date to the past 1000 years (10, 12, 74, 85-89). It is believed that tunas were captured both inshore and offshore. This includes directly from the coast using hook and line where there is a rapid drop-off to deep water, from the beach using coconut frond sweeps and seine nets when migratory schools pass islands, and through trolling from outrigger canoes (10, 65, 90, 91). Skipjack trolling is viewed as sacred in many parts of the Pacific, for example, on Ifaluk Atoll (Caroline Islands), this type of fishing is associated with five of the 16 known fishing songs (91). Archaeologically, Weisler et al. [2024 (89)] reported the religious use of skipjack in the Tuamotus from 1400 years ago through their offering to sacred sites known as marae. A broad decline in tunas relative to inshore coral reef species have been reported from these Pacific sites, where it has been argued that island colonization phase assemblages are more strongly associated with tuna fisheries, particularly in eastern Polynesia [e.g. (67, 74)], and then overtime there is an adoption of locally specific

fishing practices [e.g. (92)]. It has also been argued that archaeological trends in skipjack relative abundance may relate to the displacement of the equatorial Pacific warm pool, due to ENSO events, which could differentially influence tuna recruitment and range in the past (10, 82, 85). Overall, the shifts in the relative abundance of tunas across Pacific archaeological sites have been attributed to changes in cultural preference and/or the availability of the resource due to climatic variability, not anthropogenic pressures (10).

Billfish fishing was more geographically restricted, with the only confirmed records from the Mariana Islands (Micronesia), and the earliest of these from the Mangilao Golf Course Site 25 (Guam) beginning ~2500 years ago (93). There is also evidence from the islands of Rota, Tinian, and Saipan, where collectively across the eight archaeological sites with billfish remains, the percent total site MNI varies between 1% and 9.2% (11, 94). These highly specialized pelagic fishing skills are not seen elsewhere in Oceania but have been compared to similar records from places like Taiwan and Philippines, with recent genetic work indicating that migration of people from the Philippines into the Marianas being a strong possibility (95).

The capture of tuna and billfish were disrupted during the recent historic period across much of Oceania. For example, in the Mariana Islands, pelagic fishing continued with the addition of iron for hooks, but following Spanish colonization in 1668 and subsequent Spanish-Chamorro Wars, the Chamorro people were not able to participate in pelagic fishing for almost 200 years (94). In Oceania, the industrialization of tuna fishing began during the early 20th century, but re-emerged post-WWII and continued to expand through to the 1980s. In Australia, the southern bluefin tuna fishery started in the 1920s off the east coast and significantly expanded in the 1950s with a pole-and-live-bait fishery off New South Wales, South Australia and Western Australia, which produced a peak catch of 21,500 tons in 1982 (96). In the Pacific,

Japanese pole-and-line fisheries were the most active in the region during the mid-20th century and targeted fish for the international cannery market, and by the 1970s, these fleets also started longlining for sashimi tunas (97). By the 1980s, US purse seiners began operating across the Western Pacific, and over the next two decades the Philippines, China, Europe, and Pacific Island countries would also become active participants in the fishing of skipjack, yellowfin, bigeye, and albacore (98). It is estimated that fisheries took ~50,000 million tons of tuna from the Pacific Ocean between 1950 and the early 21st century (97), with considerably less, but not insignificant numbers, caught by Australia's tuna and billfish fisheries of the 1980s onwards (99). Collectively, a mere century of industrialized fishing in Oceania surpassed and depleted the stocks that Indigenous fishers were able to engage with for millennia.

SI-1.5. ***Japan, Korea, and China.*** The first evidence for tuna fishing from Japan dates to the early part of the Initial Jōmon phase (ca. 10,500-5000 BCE) at sites including Natsushima and Shimotakabora (8). The latter site, on Ōshima Island in the Izu chain, also produced abundant skipjack tuna remains from Late-Final Jōmon layers (ca. 2500-500 BCE) (100). Overall, the most important Jōmon fisheries were inshore, freshwater and anadromous (salmon); the contribution of tuna and other deepwater fish was less significant although poorly quantified. Tuna are especially common in Jōmon shell middens on the Pacific coast around Sendai where the fish were caught during seasonal migrations (101, 102). On the Sea of Japan, the Torihama site produced bonito and *Thunnus* sp. from Early Jōmon layers plus one tuna/bonito bone from an Incipient Jōmon (ca. 14,500-9500 BCE) context (103).

In Japanese the generic term *katsuo* is often used to refer to both *Katsuwonus pelamis* (skipjack tuna, J. *katsuo*) and *Sarda orientalis* (striped bonito, J. *ha-katsuo*). In early Japanese

texts from the eighth century CE, the name is written with Chinese characters meaning 'hard fish' (presumably referring to a processing technique), further complicating the identification. Writing in English, Japanese archaeologists invariably translate *katsuo* as 'bonito' even when *K. pelamis* is more likely.

Billfish are known from the Kayama shell mound in an Initial Jōmon context dating to ca. 6000 BCE (9) and have been identified at a number of later sites, including the Early Jōmon Haneo shell mound (ca 4500-3500 BCE). In later periods, swordfish fishing became especially common in Hokkaido as evidenced at the Tenneru 1 site in Kushiro, dating to the early part of the Late Jōmon (ca. 2000-1500 BCE). This continued into ethnographic times with swordfish hunting by the Ainu. The 17th-century Shinotai A site produced swordfish bills inserted into the sand of a beach, likely in a type of sending-back ceremony akin to the Ainu *iyomante* (bear ceremony) (104).

After cereal agriculture reached Japan in the Bronze Age Yayoi period (1000 BCE – 250 CE), an overall decline in fishing included deepwater species. However, the following Kofun (250-710 CE) and Nara (710-794 CE) periods saw specialist targeting of skipjack tuna, particularly in the Kanto region (105, 106). Abundant skipjack tuna remains have been reported from the Hamamoroiso (Kanagawa, 7-10th centuries) and Sawabe (Chiba, 6-7th centuries) sites. A distinctive type of antler lure found at these sites is thought to have been used in this fishing, an interpretation supported by a story in the *Takahashi ujibumi*, a text compiled before 789 (107). Written sources support the importance of skipjack tuna fishing at this time. Some 190 inscribed wooden tablets (*mokkan*) used as labels for tax shipments mention *katsuo* in the eighth century but there are no examples of tuna. As recorded in the *Engishiki* (927), *katsuo*—together with abalone, various seaweeds, and dried salmon—was a common offering during a range of courtly

rituals (108). Most of this fish is thought to have been dried into hard blocks used for seasoning perhaps something like modern *katsuo-bushi*. This became one of the main uses of skipjack tuna/bonito in premodern Japan.

The importance of skipjack tuna and bonito in the Kofun (250-710 CE) and Nara (710-794 CE) periods raises several questions about the role of fishing in early state economies. For many states, the appropriation of cereals was the most secure pathway to power. Scott [2017; (109)] insists that from the viewpoint of the state tax collector, hunting, gathering, fishing, horticulture, pastoralism, and other 'nonappropriable subsistence practices' were 'fiscally sterile', meaning that they brought minimal revenue against the costs of controlling dispersed populations on the margins of 'grain cores.' However, this approach underplays the diversity of subsistence systems engaged in by peasant populations within early states (110-112). Furthermore, prestige goods obtained through controlled ('attached') specialized production or by access to exotic exchange networks could be another pathway to power (113).

From written sources we know that a range of products, including marine resources, were offered as tax or tribute to the early Japanese state by at least the sixth century (114). The Japanese literature has tended to frame its discussion around the assumption that the state controlled, or aimed to control, the production of marine products through attached specialist groups (115, 116). This connects to a broader debate in Kofun archaeology over whether regional tomb mounds were constructed by central elites or by regional chiefs (117). Comparative perspectives from the Roman empire suggest the need to consider a broader range of economic systems for seafood production in early Japan (118). As regards skipjack tuna and bonito, further research is needed to understand the organization of the fisheries. An important site in this respect in the Iwama rock shelter, a coastal burial site in Wakayama prefecture (119, 120).

Burials dating from the mid-fifth to late sixth centuries contain a range of grave goods linked with fishing, including both deer antler and iron fish hooks, a bone gorge, and an iron harpoon. The largest antler fish hook is 10 cm long and was likely used for skipjack tuna and bonito on the basis of zooarchaeological evidence. Iwama produced only a small (n=16) sample of faunal remains but six were identified as *K. pelamis* in the Japanese site report (119) but glossed as 'bonito' in the English summary (120). According to the excavators, antler sword fittings from Grave 1 engraved with a *chokkomon* design suggest links with the central Yamato polity. However, the *chokkomon* design is found in a range of different mortuary and other contexts and an exclusive association with Yamato power seems unlikely.

Since Iwama is primarily a burial site, the nature of the *K. pelamis* fishery is unclear. By contrast, other sites in Shizuoka prefecture provide evidence for large-scale processing of skipjack tuna in a particular type of large (15-20 liter capacity) ceramic vessels. A recent lipid analysis supported the use of these vessels in fish processing (106) and the identification of skipjack tuna and/or bonito is supported by documentary evidence (105). The Japanese literature summarized by Murakami et al. (106) argues that archaeological evidence such as inkstands for writing and the presence of large storehouses supports a link between the sites with the fish processing vessels and the Yamato state. However, further research is needed to elucidate the role of local versus central elites in the tuna/bonito fisheries of the Kofun and Nara periods.

A database of depictions of fish and other marine life from prehistoric Japan includes only one possible example of a tuna or billfish. The example is a fish depicted on a Late Kofun tomb wall with previous studies proposing an identification as either bonito or mullet (121).

Two important medieval sites are Yuigahama-minami in Kamakura, the capital of the medieval shoguns (1185-1333), and Tadehara-higashi on the Miura peninsula. Compared to Late

Antiquity, the percentage of skipjack tuna and bonito remains declined and more tuna was found together with Japanese Spanish mackerel and common dolphinfish. While these fish are thought to have been caught with lures, finds of sharks, swordfish and dolphins suggest harpooning (107).

In the late 16th century, salted tuna was traded from Japan to the Spanish colony of Manila (122). Dried 'bonito' (sometimes written as *bonita*) is mentioned in the diary of Richard Cocks (1565-1624), head of the English East India Company in Japan (123), although to what extent such writers distinguished between tuna and bonito at the time is moot. Although the early modern Tokugawa period (1603-1868) saw strict limits on overseas voyaging and the size of sailing vessels (124), tuna gained a new popularity from the late 18th century. While the *Efu fuzokushi* (1744-48) had noted that 'Tuna, sweet potato, pumpkin, and such are exceedingly low class foods, and even commoners are ashamed to eat them openly', the *Asukagawa* (1810) explained that "In the past, hearing someone speaking of eating tuna was shameful, but it is curious that now it is served even on special occasions." In the same year, the *Bukō Nempyō* recorded a catch of 10,000 tuna per day in Izu and Sagami provinces (125). While this number may have been exaggerated or rounded up, it can be compared with a catch of 10,000 tuna *per year* recorded from Cadiz in the 16th century (126). People in Edo (modern Tokyo) began to spend large amounts of money to eat the first catch of skipjack tuna of the year (127). The increased popularity of tuna in the late 18th century is supported by Kaibara Ekken's late 17th century description of Fukuoka domain (north Kyushu) that included detailed accounts of local fisheries but did not mention tuna (128). A late 18th century growth in tuna catches in Fukuoka seems to have been helped by the introduction of tuna trap nets, which are first reported in Japan

in the Nagasaki area in the early 17th century, though those nets only slowly spread to other parts of Japan over the following two centuries (129).

Beginning in the 20th century, long-distance fishing in motor-powered trawlers paralleled the expansion of Japan's Pacific colonial empire. Japan's acquisition of Micronesia in 1914 enabled tuna fishing across the southwest Pacific. Tuna, crab and salmon were caught primarily for canning to export to Europe and North America for foreign exchange (130). From the 1960s, the growing availability of refrigerators fueled the popularity of tuna in *sushi* and *sashimi* (131).

Compared with Japan, there is less research on the prehistory of deep-sea fishing in Korea (132). *Thunnus* sp. and Xiphiidae have been reported from a few Neolithic (Chulmun) sites including Tongsamdong (perhaps as early as ca 8000 BCE), Yŏndaedo (a site with two C14 dates on shell spanning 5459-4544 cal BCE) and K'ungokri (Bronze-Iron Age) (133, 134). However, chronological controls for these finds are not precise. Tuna was rarely eaten in Korea until the 1980s when companies engaged in long-distance fishing for export began to promote canned tuna to the domestic market as a result of increased international competition and the UN's ratification of the 200 nautical miles exclusive economic zone in 1982 (135). Advertising presented canned tuna as a high-class food through which Koreans could keep up with developed countries. One 1983 advert read "Tuna, the nobility of the sea, lives in the crystal-clear waters of the South Pacific, and only the people in wealthy countries could afford it. Now that we are well-off, it's time to bring that luxury to our tables" (135).

Archaeological evidence of tuna or billfish has been identified at only a few sites in China, often with considerable uncertainty. Relatively secure tuna remains were reported at Tianluoshan, Zhejiang province, associated with the Hemudu Culture (5000–3300 BCE), appearing alongside predominantly freshwater taxa (136). At Jiwanggu, Shandong province

(Spring and Autumn period, 770–476 BCE), fish remains from burial contexts were tentatively attributed to *Scomberomorus niphonius*, but with an uncertainty that makes possible the presence of true tuna at the site (137). Similarly, the Dazuozi site in Dalian (Bronze Age, ca. 2000-1000 BCE) produced two lower jawbones from the suborder Scombroidei—which includes tuna—though no definitive identification has been made (138). There is little other evidence of pelagic fishing from prehistoric coastal China (139). The recovery of fish remains in Chinese archaeological contexts is usually limited, partly due to the lack of systematic sieving at many sites. However, the notable absence of tuna in early Chinese historical texts and artistic depictions (140) supports its general unpopularity within early Chinese food culture and society. In contrast to mainland China, offshore in Taiwan abundant archaeological billfish (Istiophoridae) remains have been identified at the Eluanbi II archaeological site dated to ~4000-2500 years ago and earlier, but no tuna have been identified (141, 142).

Despite China's extensive coastline, freshwater fish remained the primary source of aquatic protein in premodern China (136, 139, 143, 144). Tuna was never incorporated into traditional Chinese cuisine, which is largely based on stir-frying, steaming, and boiling—preparation methods poorly suited to tuna. Although trepang, abalone, kelp and other marine products were exported from Japan to early modern China, *katsuo-bushi* bonito shavings were not widely used in Chinese cuisine until recently. Fish consumption has increased in China over the past two decades under the influence of Japanese *sushi* culture (145, 146). However, tuna remains relatively unpopular among the general population. Instead, China has become a major producer in global tuna fisheries, with much of its catch destined for export (147), though there are signs that domestic consumption is increasing at large urban centers.

Kōmoto (148) published lists of reported prehistoric faunal remains from Kumamoto, Miyazaki, Kagoshima and Okinawa prefectures in southern Japan. The information was collated by archaeologists employed by the respective prefectural governments and can be considered comprehensive at time of publication. The total number of sites was as follows: Kumamoto, 59; Miyazaki, 8; Kagoshima, 36; and Okinawa, 60. None of the fish listed in Table S3 were reported from Kumamoto or Miyazaki. Kagoshima had 4 relevant sites, all dating to the Late Jomon: *Thunnus* sp. from Kusano shell midden, *Thunnus orientalis* from the Narikawa I site, *Katsuwonus pelamis* from Ichiki shell midden, and possible *Thunnus* sp. remains from Take shell midden. From Okinawa there were two sites, both with remains of *Katsuwonus pelamis*, from Iha shell midden (Early IV) and from Gushibaru shell midden (date of the fish remains uncertain). Of the 163 sites collated by Kōmoto (148), only around 3% had remains of the species in Table S4.

Kōmoto (133) also collated faunal remains from 52 sites around the East China Sea. Of the species in Table S2, none were reported from the 33 sites in China. From Korea, out of a total of 17 sites, the K'ungokri site (Bronze – early Iron Age) had Xiphiidae and the Youndaedo site (Early – Middle Neolithic) had *Thunnus* sp. and Xiphiidae. In the Primorye province of the Russian Far East there were only two sites, neither of which had the reported species.

Table S3. Species considered from Japan.

**Species**	**English common name**	**Standard Japanese name**
Xiphias gladius	Swordfish	*mekajiki*
Istiophorus platypterus	Indo-Pacific sailfish	*bashōkajiki*
Istiompax indica	Black marlin	*shirokajiki*
Makaira mazara	Indo-Pacific blue marlin	*kurokajiki*
Tetrapturus angustirostris	Shortbill spearfish	*fūraikajiki*
Kajikia audax	Striped marlin	*makajiki*
Thunnus alalunga	Albacore	*binnaga*
Thunnus obesus	Bigeye tuna	*mebachi*
Thunnus orientalis	Pacific bluefin tuna	*kuromaguro*

Thunnus albacares	Yellowfin tuna	*kihada*
Katsuwonus pelamis	Skipjack	*katsuo*

Table S4. Early (ca. 5000-3500 BCE) and Middle (3500-2500 BCE) Jōmon tuna and skipjack remains from the Miyano shell midden (Iwate prefecture). Data from Satō & Yoshino (102).

	Middle Jōmon		Late Jōmon						Totals	
	Layer 4		Layer 5		Layer 6		Layer 7			
Taxon	NISP	MNI	NISP	MNI	NISP	MNI	NISP	MNI	NISP	MNI
Katsuwonus pelamis	903	19	42	2	244	8	113	4	1302	33
Thunnus sp.	63	2	282	6	276	5	159	4	784	17
Thunnus orientalis			2	1			4	3	6	4

SI-1.6. ***North American Pacific Coast*****.** People have fished in the waters of the Pacific Coast of North America for over 10,000 years, generally focusing on nearshore kelp forest, rocky reef, sandy beach, and estuary habitats (149-152). From Alaska to Baja California, Indigenous peoples captured fish using a wide variety of technologies, including hook and line, spears and harpoons, nets, traps, and weirs, all of which were facilitated by advancements in watercraft (e.g., plank canoes) (81, 153). With a few exceptions, people throughout the Holocene generally concentrated on fishes in inshore waters, such as salmon (*Oncorhynchus* spp.), rockfish (*Sebastes* spp.), flatfish (Pleuronectidae), surfperch (Embiotocidae), and California sheephead (*Bodianus pulcher*), with regional variation in harvested species associated with local water temperature and habitat availability (149-152). The importance of fish as a food resource also varies temporally alongside shellfish, marine mammals, and a variety of other species. Tunas, billfishes, and other large oceanic/offshore fishes were captured by Indigenous peoples in the region, but in relatively circumscribed areas and generally later in time (23, 27, 154).

Archaeological data demonstrate that tuna fishing in this region was confined to portions of British Columbia and Washington, the southern California Coast, and Baja California Sur (23,

27, 155). In parts of northern Washington and Vancouver Island a specialized Middle and Late Holocene fishery targeting large bluefin tunas was in place for warm water periods that pulled bluefin north to this area (27). There is no evidence for Indigenous fisheries for tunas from Point Conception to far northern Washington. In southern California, fifty-three archaeological sites contain the remains of albacore, yellowfin, bluefin, and skipjack, dated from 5000 years ago (and possibly earlier) through the 19th century and generally in small numbers (23). Thus, it appears tuna fishing was relatively limited and people may have targeted these fishes when they came inshore in areas like submarine canyons or seasonally just beyond kelp beds. In contrast to this general trend, Santa Catalina Island (SCAI) appears to have been a key place for Indigenous tuna fishing in southern California. In a few SCAI assemblages, tunas make up 13-35 percent of the entire fish NISP, whereas at most other sites tunas are 4% and often less than 1% of the identified fish (23, 153, 154). In Baja California, there is limited archaeological evidence for tuna fishing, though more detailed zooarchaeological studies are needed (23). Tunas are rare to absent for most of Baja California but are present in trans-Holocene deposits at the Covacha Babisuri site near La Paz where they make up some 10% of the fish assemblage and might be 10,000 years old or older (156).

Billfish fishing was even more localized, confined to southern California, especially on the Santa Barbara Channel and Channel Islands, where billfishes were caught in a highly specialized and ritualized fishery beginning at least 1500 years ago. Recent evaluation of these archaeological materials via ZooMS (157) confirm that both swordfish (*Xiphias gladius*) and striped marlin (*Kajikia audax*) were fished by local communities, though swordfish remains were more abundant (158). Subsequent isotopic analysis of the same fish bones demonstrated that these billfish were coming from a range of source populations within both the California Current

System and North Pacific Subtropical Gyre (159). Again, billfish were never a major component of people's diets but appear to have been associated with rituals, feasting, and ceremony (25).

After most Indigenous peoples had been removed to mainland missions following disease induced population declines, tuna and billfish fishing largely ceased in the area for about a century until the end of the 19th century, when fisheries initiated largely by Japanese and Portuguese immigrants started a rapid growth in regional tuna fishing (23, 160). By the early 20th century, tuna fishing had greatly increased, reaching its zenith in the 1940s and 50s. During this time, people fished for albacores, bluefins, yellowfins, and skipjacks in southern California and Baja, with albacore targeted offshore in the Pacific Northwest, and tunas also imported (23). By the 1950s to 70s in tandem with global declines, tuna fishing had greatly decreased in southern California. The vast quantities of tunas taken by 20th century and modern commercial and recreational fisheries, including tens of millions of pounds of tunas annually at its mid-20th century peak, stand in sharp contrast to the millennial scale, but more limited catch of these large offshore fishes by Indigenous people (23).

SI-1.7. *Ecuador to Chile.* The Salango archaeological site is one of the most important coastal sites on the Ecuadorian shoreline. Its chronostratigraphic sequence spans nearly 5,000 years, providing a rich and unique record of pre-Hispanic occupation along the country's central coast (161, 162). It is an ideal location for investigating cultural and technological developments, and in the case of the present study, those related to fishing practices. From the Valdivia period (3800–1500 BCE) to the Manteño period (700–1532 CE), the occupation of Salango is marked by an intensive exploitation of marine resources, particularly fish (20, 163). Analysis of tens of

thousands of bones from all cultural phases reveals clear trends, most notably a shift toward the capture of scombrids (tunas and bonitos).

Salango's archaeological fish assemblage is the richest and most diverse on the Pacific coast of the Americas in terms of both NISP and the number of scombrid species—especially true tunas (Thunnini)—represented at the site. It is the only known Pacific coastal site in the Americas where *Thunnus albacares* (yellowfin tuna), *Katsuwonus pelamis* (skipjack), *Euthynnus lineatus* (black skipjack), and *Auxis* spp. (frigate mackerels) have all been positively identified (20). Pelagic scombrids dominate the assemblage throughout the entire sequence, increasing from 40% during the Valdivia period to 84% during the Manteño period. Among the Thunnini, black skipjack becomes the most abundant species in the later phases, rising dramatically over time (from 1.4% to 90%), while yellowfin tuna declines from 55% to 0.4%.

Comparisons with geographically proximate archaeological sites suggest that this type of fishing strategy was specific to Salango. Fishers there likely took advantage of the site's strategic location at the southern end of a bay that is partially enclosed by an island but separated from the mainland by only a 1 km stretch of water. They probably understood the migratory behavior of scombrids passing close to the coast and exploited this resource intensively. It is plausible that all the scombrids found at Salango could have been captured without venturing into the open ocean.

Today in Manabí, pelagic fisheries are dominated by common dolphinfish. Coastal fishing for Thunnini is relatively limited, as schools have shifted farther offshore, where they are caught using trolling gear or purse seines. The black skipjack is now nearly absent from modern catches (164).

South of Ecuador, the cold waters of the Humboldt ecosystem do not favor the presence of tunas along the coast. No archaeological site on the Peruvian shoreline contains significant

quantities of Thunnini (165). Pre-Hispanic fisheries were instead focused on the extremely abundant small pelagics—such as anchovies, sardines, and jack mackerels—as well as on coastal demersal species, particularly drums (166, 167). In contrast, in northern Chile, along the coastal Atacama Desert (ca. 20°–27°S), several excavations have uncovered remains of large pelagic fishes (168, 169). The Zapatero shell midden, dated to 7400–5900 years ago, is remarkable for its high density of billfish remains—*Kajikia audax* (striped marlin) and *Xiphias gladius* (swordfish)—accompanied by a few rare tunas, *Katsuwonus pelamis* and *Thunnus* sp. (22). Although billfishes account for only 0.69% of the total NISP, their combined weight represents 5.75% of the total assemblage, with a minimum of 42 individuals identified.

This billfish fishery, dated to over 6000 years ago, is one of the oldest currently known worldwide. It raises questions about the reasons for its existence in a sea where other marine resources were plentiful, as well as about the technical methods used to carry it out, and the cultural significance of the fishery [e.g., (170)]. It is particularly noteworthy that coastal harpoon fishing for swordfish is still practiced in Taltal during the austral spring and summer (171). Motorized boats are equipped with a gangway at the front that allows harpooners to approach their prey as closely as possible.

SI-1.8. ***Brazil.*** The Brazilian coast is globally recognized for its ecological significance and long history of human occupation, dating back over 10,000-9000 years ago (172-175). This extensive area and timeline are archaeologically marked by a variety of cultural groups that continuously or recurrently occupied and exploited diverse environments on the Atlantic coast, such as estuaries, bays, and mangroves, throughout the Holocene (172, 176-180). The southern Brazilian shelf (22°S to 34°S; 270,000 km²) comprises four distinct sectors, ranging from the narrow,

current-influenced Cabo Frio–Cabo de São Tomé shelf to the complex, channel-incised São Paulo Bight. Southward, the Florianópolis–Mostardas sector is relatively uniform, while the Rio Grande Cone hosts sand banks and scarps linked to sea-level stillstands since the Last Glacial Maximum (181). Moreover, it is important to note that relative sea-level (RSL) variations have shifted the Brazilian coastline over time (182, 183).

The southeastern and southern coasts have been the focus of archaeological research. This pattern is probably due to the high density of archaeological sites, particularly the monumental sambaquis (176). Zooarchaeological and stable isotope data available from this region have demonstrated the central role of aquatic and coastal resources in people's diet (18, 19, 184-187). A wide range of fishing technologies was employed by the coastal groups, including single-piece baited fish hooks, projectile points, net weights and cordage made from plant fibers with knots used by fishers in contemporary periods (188-191). Cordages are exceptionally well preserved in Cubatão I and Espinheiros II due to anaerobic conditions (192, 193).

Bony and cartilaginous fish were the primary aquatic resources exploited by the cultural groups, representing more than 140 species. The most important species include whitemouth croaker (*Micropogonias furnieri*), black drum (*Pogonias cromis* or *P. courbina*), white sea catfish (*Genidens barbus*), sand tiger shark (*Carcharias taurus*), tiger shark (*Galeocerdo cuvier*), and other species (18, 19, 185, 186). However, it is also worth noting that recent studies have increasingly focused on tetrapod remains (e.g. marine mammals), which provide complementary insights into the broader spectrum of exploited coastal fauna and enhance our understanding of subsistence strategies during the pre-colonial period (194, 195). Recent studies suggest that tuna and billfish have a low frequency in the archaeological record on the Brazilian coast (18, 19,

186). Billfish, especially sailfish (Istiophoridae - *Istiophorus albicans*), and specimens of the mackerel and tuna family (Scombridae - *Katsuwonus pelamis*, *Scomberomorus* cf. *brasiliensis* and *Thunnus* cf. *atlanticus*) were recovered in a few shellmounds located in coastal and rocky island oceanfront areas between latitude 20°S and 25°S on the southeastern coast and dated between 4530-4400 years ago and 1765-1265 years ago (18, 19). Along the southern coast, between latitudes 25°S and 31.5°S, billfish and tuna were not identified in archaeological assemblages (186). This absence may primarily reflect the species' biogeography and environmental factors. First, regional oceanographic differences, including stronger upwelling and higher productivity in the southeast (e.g. Rio de Janeiro), could have concentrated tuna and billfish there, making them more available than in southern waters (196). Second, the wider continental shelf in the south probably limited access to deep-water habitats, reducing opportunities to capture large pelagic fish (181). Third, Middle to Late Holocene sea-level variations favored the formation of paleo-lagoons and estuaries in the south (197-200), ecosystems highly productive for coastal species (201, 202), which may have reduced the need for shelf and offshore exploitation. Another explanation can be based on the methodological approaches and taphonomic biases, such as mesh size used in excavation, degree of remains fragmentation and gaps in reference collections (186). With respect to this latter point, most analyzed ichthyological assemblages indicate that between ≥50% and 99.46% of the remains were exclusively identified as Actinopterygii and Elasmobranchii (186).

During the European contact period, aquatic resources remained essential to subsistence and regional economic development. Between 1650 and 1850 CE, species such as whitemouth croaker, black drum, white sea catfish, barred grunt (*Conodon nobilis*), snook (*Centropomus* spp.), and weakfish (*Cynoscion* sp.) continued to play a key role in the subsistence strategies of

coastal populations (203). After the 1880s, mullet (Mugilidae, *Mugil liza*) and shrimp (Penaeidae) emerged as the main fishing targets (204). Tuna (*Thunnus* sp.) and billfish (*Xiphias gladius*) were occasionally captured between the 1970s and 2000s, and began to gain prominence in national fisheries in the early 21st century (204, 205).

SI-1.9. *Caribbean and Southeastern United States.* Together, the marine waters and shorelines of the southeastern United States and insular Caribbean have long supported some of the most ecologically and biodiverse fisheries in human history, including a variety of vertebrate and invertebrate species from estuarine, seagrass, coral reef, and pelagic habitats. From an archaeological perspective, people have lived in the southeastern U.S.A. since approximately 14,500 years ago and among the Caribbean islands since circa 6000 years ago (206, 207). However, archaeological evidence for the antiquity of fishing within the contiguous maritime regions largely post-dates human arrival by millennia due to a relative dearth of archaeological documentation at early sites compared to those from later time periods (e.g., Late Holocene, post-6000 years ago), sea level rise and site submersion, and/or rampant coastal development since the 20th century (208, 209). For the southeastern U.S.A., from the Gulf of Mexico in Louisiana to the Florida Keys and along the Atlantic Coast from North Carolina to southeast Florida, archaeological evidence indicates people were engaging coastal resources by at least the Middle Holocene (e.g., ~7000 years ago), followed by more sedentary coastal lifeways and persistent harvest by ca. 4000 years ago (210-212). Across the Caribbean islands, including from The Bahamas south to Trinidad and Tobago off the coast of South America, sustained coastal resource harvest dates to at least 3000 years ago on some islands [e.g., Saba (213); Tobago (3)] and as recently as 1300 years ago on others [e.g., The Bahamas (214, 215)].

Spanning temperate to neotropical climate regimes and buffered by relatively wide and shallow continental shelves, the shorelines of the southeastern U.S. are characterized by vast expanses of estuarine ecosystems, including various combinations of tidal creeks, marshes, mangroves, sea grasses, and islands (e.g., Outer Banks, Golden Isles, Florida Keys, Big Bend). By the Middle to Late Holocene, this coastal region was home to diverse Indigenous communities [e.g., (211, 216, 217)] and coastal animal harvest was by and large focused on bivalves, especially oysters (e.g., *Crassostrea virginica*) [e.g., (218-220)]. The bounty of estuarine resources and animal harvest have been implicated in the emergence of some of the earliest established villages, civic ceremonial centers, and socially complex polities in North America as evidenced by extensive shellwork constructions (e.g., middens, mounds, rings, ridges) and hydrological engineering (e.g., canals, fish traps) across both the Gulf and Atlantic coasts (221-224). For at least four millennia, people focused on the harvest of inshore fishes associated with relatively shallow habitats using mass capture techniques (e.g., seins, weirs, nets, and traps) [e.g., (225-228)]. Common species identified from Atlantic and Gulf coast zooarchaeological assemblages include herrings (Clupeidae), sea catfishes (Ariidae), mullet (Mugilidae), drums (Sciaenidae), sheepshead (Sparidae), snooks (Centropomidae), and jacks (Carangidae) [e.g., (216, 229-235)]. Offshore fishing was not a significant focus of Indigenous fisheries, with billfishes and tuna species rarely present in the regional zooarchaeological record [e.g., Clupper site, FL (4); Tibwin Trail site, SC (236)]. Generally, across the southeastern U.S., concerted efforts towards offshore fishing began in limited capacity following the arrival of European colonists and African slaves during the 16th to 17th centuries, but did not develop in earnest until the 19th century [e.g., (237)].

Situated between the Gulf of Mexico, Caribbean Sea, and the Atlantic Ocean, the insular Caribbean is a global biodiversity hotspot composed of thousands of islands, cays, and islets surrounded by 4 million km^2 of sea that supports the highest marine species richness in the Atlantic Ocean and 10% of global coral reefs (238, 239). Across the region, marine diversity is concentrated within interconnected, relatively shallow, nearshore environments (e.g., coral reefs, mangroves, sea grass beds, estuaries, and lagoons) that are positioned along relatively short continental shelves bordered by the Atlantic Ocean to the east and/or deepwater basins in the Caribbean Sea to the west (e.g., Yucatan Basin, Colombia Basin, Venezuelan Basin). The majority of Holocene marine resource harvest has been documented from coastal archaeological sites dating to the Ceramic Age (500 BCE-1500 CE), and especially from the latter half of this time span (i.e., post 500 CE) when Indigenous communities were present across much of the archipelago (240, 241). Fish and the act of fishing were significant elements of Indigenous lifeways, with connections to origin myths (242) and providing the foundation of dietary protein (241, 243, 244). The harvest of nearshore, coral reef-associated fishes was a primary focus of subsistence economies overall and a relatively common suite of fishes are represented in the Caribbean zooarchaeological record, including parrotfishes (Labridae), grunts (Haemulidae), snappers (Lutjanidae), grouper (Epinephelidae), jacks (Carangidae), and surgeon fishes (Acanthuridae) (241, 245-250).

Offshore species are well represented across Late Holocene insular Caribbean sites in terms of geographic breadth, if not necessarily relative abundance, including tuna (i.e., Family: Scombridae) identifications reported from dozens of sites from The Bahamas through the Greater and Lesser Antilles, and south in Tobago. More specific to this synthesis, Thunnini genus or species-level identifications indicate harvest and consumption of tuna at a minimum of 31 sites

across 20 islands. Taxa include: *Thunnus* sp., *Katsuwonus pelamis*, *Euthynnus alletteratus*, *Scomberomorus* sp., and *Auxis* sp. [e.g., (245, 251-254)]. The temporal spectrum of fishing represented by these species is at least 3000 – 300 years ago. While Thunnini rarely account for the majority of fishes present, it is worth noting they are most abundantly reported from sites in the Lesser Antilles and Tobago (e.g., Sabazan Carriacou [The Grenadines, Lesser Antilles; (247, 255)], the Milford 1 and Golden Grove sites on Tobago (3), and St. John on Trinidad (2)). This is in part likely tied to the fact that the southeastern Caribbean is one of the more prominent western Atlantic Ocean spawning grounds and migration pathways for tuna species, where they are accessible throughout the year (256). Indigenous tuna fishing strategies in the insular Caribbean have been attributed to hook and line, or spear, fishing from canoes "offshore" but it is worth noting tuna species are also known to frequent inshore waters for feeding. Much like the southeastern U.S., offshore fishing for pelagic species is a relatively recent development in Caribbean fisheries dating to the mid-19th – 20th centuries [e.g., (257-259)].

SI-1.10. *Gulf of Maine and New England.* Although no tuna bones have been identified, an Indigenous fishery for swordfish (*X. gladius*) began around the Gulf of Maine before 5500 years ago and ended with abrupt local environmental change (cooling) about 3700 years ago. The Gulf of Maine is a semi-enclosed basin, with deep water influence from northern cold currents (260). For the past 3500+ years, it has been macro-tidal, with common tidal amplitudes of 4-5 meters increasing to 8-10 meters or more in the easternmost Bay of Fundy. The tidal amplitude drives cold-water upwelling and creates a cold, inshore ecology with extensive intertidal shellfish flats and wide beach locations suitable for intertidal fish weir construction. Prior to 3700 years ago, a micro-tidal environment with a relatively warm surface water layer during summer likely

attracted swordfish to swim inshore. As post-glacial relative sea level rose, geologists reconstruct a "catastrophic" event that at about 3500 years ago cut through a glacial till ridge in the Bay of Fundy and allowed flooding of the Minas Basin (261). That event likely increased tidal amplitude dramatically and was followed by rapid marine cooling.

Due to land subsidence and sea level rise, the oldest surviving on-shore midden component dates to 5000 years ago [Turner Farm site, (5, 262)]. At least eight sites on the central Maine coast (greater Penobscot Bay) contain swordfish rostrum (sword) and postcrania in components dating between roughly 4200 and 3750 years ago (263). In the best-preserved shell midden deposits, postcrania of large swordfish include whole and fragmentary vertebrae, rib and fin "ray" bones, in addition to large and small pieces of rostrum. Entire large (>100 kg) swordfish were being returned to these sites. Swordfish rostrum was used as a tool raw material, including as foreshafts in toggling harpoons (264). A stabbing weapon technology made of moose shin bone daggers and ground slate points was likely used to dispatch swordfish at sea, likely from dugout canoes, in areas where swordfish came inshore (263). Swordfish fishing was part of broader fishing traditions that included bone hook, line and stone sinker fishing for cod in deep water (264); spring harvest of river herring (alewife) runs at stream and river locations; and multi-seasonal hunting for deer, bear, and fur-bearers on shore (5).

Central Gulf of Maine swordfish fishing has been associated by some archaeologists with the Moorehead phase elaboration of mortuary ceremonialism (264), with clear cultural connections to the Maritime Archaic cultures and mortuary practices of the Gulf of St. Lawrence, Newfoundland, and the Labrador coast to the north. Around the southwestern Gulf of Maine, two island midden sites in Casco Bay record swordfish fishing in components dated to 3800-4000

years ago (263). Further southwest in New Hampshire, a cache of four swordfish rostra was recovered from an intertidal site in Seabrook Marsh, of similar age (265). In eastern Maine, relative sea level rise has inundated all sites older than about 3000 years. However, two near-coastal lacustrine sites have yielded calcined swordfish rostrum fragments and a calcined swordfish rostrum barbed harpoon, tying seasonal transhumance patterns to coastal seasonal swordfish fishing at about 4000 and 5500 years ago (266, 267). Neither the eastern nor westernmost Gulf of Maine swordfish use was associated with the Moorehead phase culture, indicating that swordfish hunting was both temporally and culturally diverse. In other words, swordfish hunting was not a necessary and sufficient correlation with the mortuary elaboration of the Moorehead phase. In any case, the Moorehead phase mortuary elaboration and swordfish fishing in the Gulf of Maine came to an abrupt end around the Gulf of Maine within a 50 year time period around 3700 years ago.

Now-submerged, formerly littoral Indigenous archaeological sites and associated fossil shellfish from as deep as 30 to 50 meters in the Gulf of Maine, dating from 9000 to about 5000 years ago, are known primarily from stone tools recovered by scallop draggers, and from geological coring and other environmental characterization (263, 268, 269). The adaptation included dugout boat-based transport (based on near beach site locations), maritime hunting with ground slate points and large flaked stone bifaces, and likely oyster and clam harvesting. Because the Early and Middle Holocene terrestrial environment was relatively warmer than it is today, and the Gulf of Maine was micro-tidal, we assume that swordfish fishing may have been part of the adaptation before 6000 years ago.

SI-1.11. ***Northeastern Atlantic/Mediterranean.*** In the northeastern Atlantic and Mediterranean, the earliest evidence of possible tuna fishing comes from Atlantic bluefin tuna vertebrae dated to ∼140,000 years ago from Svenborg (Denmark, Pers. Comm. K. Kjaer (6)), and from Neanderthal levels (28,000–24,000 years ago) in Gorham's Cave [Gibraltar, (270)]. Both of these finds are debated, but are generally considered cases of opportunistic scavenging, rather than fishing, and very little is known about the specimens from Denmark at all making them highly questionable (271).

Vertebrae from numerous sites dating to 11,000–7800 years ago in Spain, France, Croatia, and Greece better document the onset of tuna fishing in Europe (6). The earliest of these of any notable number (NISP∼5000) are bluefin tuna vertebrae from Franchthi Cave [Greece, ∼11000-9000 years ago, (272)]. Limited amounts of swordfish (*Xiphias gladius*) have also been reported by 10,000-9000 years ago at Vela Spila, Croatia (7) demonstrating that swordfish were at least occasionally being caught in Europe by the Early Holocene. Little tunny (*Euthunnus alleteratus*) remains appear later in a range of Greek sites by around 6300 years ago, but small amounts appear by ~9000 years ago at Vela Spila (273). A key occurrence are those at Saliagos that were initially misidentified as bluefin tuna [Greece, 6300 years ago, (274)]. Evidence for another relevant species in the region, albacore (*Thunnus alalunga*), is limited; perhaps due to identification challenges, where it is mistaken as juvenile bluefin tuna, but also due to its offshore migrations (6, 275).

Mesolithic and Neolithic European tuna fishing likely occurred inshore (276) with hook and line or harpoon being the predominant fishing methods where fish could have been “herded” or driven inshore (277, 278). Very large individuals (> 200 cm in length) were mostly avoided

(279), indicating technological challenges and possibly an inability to target spawning migrations without nets until antiquity (6).

An analysis of archaeological bluefin tuna vertebrae throughout time (6) indicates that it is not until antiquity that the number of fishes caught significantly increases; and this coincides with the development of facilities vital for fishing (nets), fish processing (salting and fermenting facilities), and trade (long-distance shipping routes). Throughout history, the number of vertebrae was a poor indicator for fishing extent because these were missing from key sites (tuna trap sites). This is inferred because bones of large fishes may not have been transported to settlement, military, and religious locations that have been the predominant focus of European archaeology (6).

Several deep investigations into bluefin tuna ecology using multidisciplinary methods recently revealed novel insights into past fishing pressures and impacts. Morphological analyses of bluefin tuna vertebrae dating back to antiquity reveal fisheries targeted smaller sizes before ca. 200-120 years ago (26) that corresponds with an increase in fishing effort, genetic bottleneck and loss of genetic diversity at around the same time point. The inclusion of Neolithic vertebrae at both the mitochondrial (280) and nuclear (281) levels suggested that fishing effort was insufficient prior to industrial times to alter population dynamics. Isotopic data, however, suggest both earlier–and later—impacts, first a shift in bluefin tuna diet away from inshore prey around 500 years ago that corresponds with fluctuating tuna trap catches over centuries and reports of medieval-era overfishing, before the loss of a Black Sea trophic niche in the industrial era at 50 years ago that is yet to return (282).

SI-2. Estimating upper-bound archaeological tuna biomass from excavated sites

To contextualize archaeological evidence for tuna and billfish exploitation relative to modern fisheries (Fig. 3 in main text), we estimated the upper bound biomass represented by tuna remains recovered from excavated archaeological sites in selected regions. Within these regions, our goal *was not* to quantify the number of individuals harvested (e.g., MNI), but rather to estimate the maximum possible regional tuna biomass represented by the current archaeological record prior to the onset of global commercial fisheries based on the available archaeological data. We calculated this as:

$$\textit{Upper bound tuna biomass from excavated sites} = NISP \times W_{max}$$

where NISP is the number of identified specimens for a given tuna species at a given archaeological site, and $W_{\max}$ is the maximum recorded whole-body mass for the corresponding taxon. Whereas NISP necessarily undercounts true archaeological catch because of taphonomic loss, incomplete excavation and reporting, and taxonomic uncertainty, $W_{\max}$ assumes that every recovered specimen represented the largest known individual of its species. As a result, this calculation provides an upper bound on the biomass represented by the available archaeological record, while still representing a conservative underestimate of total pre-industrial tuna removals. Max species-level body masses in kg were derived from FishBase (196) and associated literature and included, from largest to smallest: *Thunnus thynnus* (684 kg), *T. orientalis* (450 kg), *T. albacares* (200 kg), *Scomberomorus commerson* (70 kg), *T. alalunga* (60 kg), *S. cavalla* (45 kg), *Katsuwonus pelamis* (34.5 kg), *Euthynnus alletteratus* (16.5 kg), *E. affinis* (14.0 kg), *S. regalis* (7.8 kg), and *Auxis thazard* (1.7 kg). Although not part of the Thunnini, *Scomberomorus* spp. were included for the Caribbean and Southwestern Indian Ocean because they are closely related

and similarly highly migratory scombrids. For the Mediterranean, we focus on Atlantic bluefin tuna following Andrews et al. (6).

We made these calculations for four regions with robust archaeological assessments of tuna remains and which also support important modern tuna stocks: (i) the Mediterranean and northeastern Atlantic, (ii) the North American Pacific coast, (iii) the Caribbean, and (iv) the western Indian Ocean. These regions were chosen because they contain well-studied archaeological records of fish remains, which have been synthesized, quantified, and were available for a broad comparative analysis. Other regions either did not have significant quantities of tuna remains available in the archaeological record (e.g., Gulf of Maine, Southeastern United States, and Brazil), were too vast or unavailable for synthesis (Oceania), or had a mix of text evidence and archaeological data (East Asia) that precluded synthesis that was comparable to the other regions.

Only specimens identified at least to genus were included in our analyses. When species-level identifications were unavailable (e.g., NISP of 6 for "*Thunnus* sp."), NISP values were multiplied by the maximum body mass of the largest species within the genus present in the region (e.g., *Thunnus thynnus* for the Atlantic, *Thunnus orientalis* for the Pacific). For sites where tuna remains were reported qualitatively, as "present" or "multiple", we assigned baseline NISP of either 1 (present) or 5 (multiple). To assess the sensitivity of our biomass estimates to this assumption, we re-ran all calculations under alternative NISP values of 1 and 10 for all qualitatively reported records. Of the 221 sites in our dataset, 52 (24%) contained at least one qualitatively reported NISP tuna record, concentrated in the Mediterranean and northeastern Atlantic (43 of 115 sites) and the North American Pacific Coast (9 of 72 sites). Varying the assumed NISP from 1 to 10 for these sites altered regional biomass estimates by no more than

4.1% and global estimates by no more than 3.2%, confirming that our biomass estimates are robust given available archaeological specimen records.

Following the temporal framework of Andrews et al. (6), we then aggregated our estimates of upper bound harvested tuna biomass for each region into four temporal bins: >900 BCE, 900 BCE–700 CE, 700 CE–1800 CE, and a generalized "Holocene" category for sites with poor chronological resolution. In cases where site chronologies did not fit exactly into these bins, such as the Mill Reef site on Antigua Island which has a chronometric age of "500-1000 CE," we assigned sites into the nearest bin. Supplemental Table S5 below presents the synthesis across the four regions with our assigned temporal bins, NISP for different scombrid species by region and age bin, and associated maximum species mass estimates.

Across all regions and time periods, these upper bound estimates of archaeological tuna biomass removals were orders of magnitude lower than modern commercial harvests. In the Mediterranean, where organized tuna fisheries emerged earlier than elsewhere, upper bound biomass represented by specimens from excavated sites was estimated at ~6 million kg (6,000 tons) over approximately 2,000 years (Fig. 3). By comparison, global annual harvest of tunas and related taxa approached 2 million tons in 1960 alone (283). These comparisons underscore the minimal scale of pre-industrial tuna exploitation and highlight the rapid, historically recent intensification of offshore fishing pressure during the industrial era.

Table S.5. Tuna NISP and associated upper bound biomass represented in the archaeological record of four key geographic regions. See supplemental section SI-2 for biomass calculations. Taxonomic names noted parenthetically denote the species used for mass calculations when records were only identified to genus; these assignments were done based on known biogeographic patterns of scombrids. Some records reported scombrid presence without a quantified NISP, such as "present" or "multiple." For biomass estimations, records reported as "present" or with no count were assigned NISP=1, and records reported as "multiple" were assigned NISP = 5. Regions and time bins which include these qualitative records are marked with an asterisk (*). AgeBins do not reflect specific age of individual deposits, instead they reflect aggregations of our data into the four temporal bins used by Andrews (6): >900 BCE, 900 BCE–700 CE, 700 CE–1800 CE, and a generalized "Holocene" category for sites with poor chronological resolution.

Ocean	AgeBin	Species	Sites	NISP	Max_kg
Caribbean	700 CE to 1800 CE	*Auxis* sp. (*thazard*)	4	218	371
Caribbean	700 CE to 1800 CE	*Euthynnus alletteratus*	10	48	792
Caribbean	700 CE to 1800 CE	*Katsuwonus pelamis*	4	847	29222
Caribbean	700 CE to 1800 CE	*Scomberomorus* sp. (*cavalla*)	8	41	1845
Caribbean	700 CE to 1800 CE	*Thunnus* sp. (*thynnus*)	9	346	236664
Caribbean	900 BCE to 700 CE	*Euthynnus alletteratus*	5	695	11468
Caribbean	900 BCE to 700 CE	*Katsuwonus pelamis*	1	74	2553
Caribbean	900 BCE to 700 CE	*Scomberomorus* sp. (*cavalla*)	1	5	225
Caribbean	900 BCE to 700 CE	*Thunnus* sp. (*thynnus*)	3	17	11628
Caribbean	>900 BCE	*Euthynnus alletteratus*	1	42	693
Caribbean	Holocene	*Auxis* sp. (*thazard*)	2	376	639
Caribbean	Holocene	*Euthynnus alletteratus*	1	3156	52074
Caribbean	Holocene	*Scomberomorus regalis*	1	2	16
Caribbean	Holocene	*Scomberomorus* sp. (*cavalla*)	1	8	360
Caribbean	Holocene	*Thunnus* sp. (*thynnus*)	2	68	46512
Mediterranean_Northeastern Atlantic	700 CE to 1800 CE	*Thunnus thynnus*	24	1621*	1108764*
Mediterranean_Northeastern Atlantic	900 BCE to 700 CE	*Thunnus thynnus*	60	3853	2635452*
Mediterranean_Northeastern Atlantic	>900 BCE	*Thunnus* sp. (*thynnus*)	1	2608	1783872
Mediterranean_Northeastern Atlantic	>900 BCE	*Thunnus thynnus*	30	796*	544464*
North_American_Pacific_Coast	700 CE to 1800 CE	*Thunnus alalunga*	16	785	47336
North_American_Pacific_Coast	700 CE to 1800 CE	*Thunnus albacares*	4	67	13400
North_American_Pacific_Coast	700 CE to 1800 CE	*Thunnus orientalis*	10	225*	101250*
North_American_Pacific_Coast	700 CE to 1800 CE	*Thunnus* sp. (*orientalis*)	15	664*	298800*
North_American_Pacific_Coast	900 BCE to 700 CE	*Thunnus alalunga*	4	17	1025
North_American_Pacific_Coast	900 BCE to 700 CE	*Thunnus orientalis*	3	38	17100
North_American_Pacific_Coast	900 BCE to 700 CE	*Thunnus* sp. (*orientalis*)	6	18*	8100*
North_American_Pacific_Coast	>900 BCE	*Thunnus alalunga*	4	490	29547
North_American_Pacific_Coast	>900 BCE	*Thunnus orientalis*	1	1	450
North_American_Pacific_Coast	>900 BCE	*Thunnus* sp. (*orientalis*)	5	68	30600

North_American_Pacific_Coast	Holocene	*Thunnus alalunga*	6	115	6935
North_American_Pacific_Coast	Holocene	*Thunnus albacares*	3	27	5400
North_American_Pacific_Coast	Holocene	*Thunnus orientalis*	8	143*	64350*
North_American_Pacific_Coast	Holocene	*Thunnus* sp. (*orientalis*)	12	173*	77850*
Southwestern_Indian_Ocean	700 CE to 1800 CE	*Euthynnus affinnis*	3	117	1638
Southwestern_Indian_Ocean	700 CE to 1800 CE	*Katsuwonus pelamis*	1	2	69
Southwestern_Indian_Ocean	700 CE to 1800 CE	*Scomberomorus commerson*	1	3	210

Table S6. Sites with archaeological tuna remains across four key geographic regions (n = 221 sites total). Column AgeBin reflects the assigned chronological period from which tuna remains were recovered, and RelativeTimeSpan denotes the likely age range of the deposits as reported by the original researchers (which may be inferred from cultural materials, stratigraphy or direct ^{14}C dating). Species identifications are drawn from original zooarchaeological reports. Where specimens could only be identified to genus (*Thunnus* sp.), the likely species is noted parenthetically in the summary biomass table (Table S5). Mediterranean site numbering follows Andrews et al. (6) ; (*) NISP of *E. alletteratus* and *Scomerbomorus* sp. are lumped from five Caribbean sites following original reporting of these data (245).

Ocean	Site(s)	RelativeTimeSpan	AgeBin	Species
Caribbean	*Barnes Bay; Sandy Ground; Shoal Bay East; Sandy Hill; Rendevous Bay	AD 500-1500	700 CE to 1800 CE	*Euthynnus alletteratus*, *Scomberomorus* sp.
Caribbean	Brighton Site	post AD 500	700 CE to 1800 CE	*Katsuwonus pelamis*
Caribbean	Calivigny Is. 67	AD 1000-1500	700 CE to 1800 CE	*Auxis* sp.
Caribbean	Chancery Lane	post AD 500	700 CE to 1800 CE	*Thunnus* sp.
Caribbean	Coralie Site	AD 700 - 1500	700 CE to 1800 CE	*Scomberomorus* sp.
Caribbean	Cranbrook	980-1100 A.D.	700 CE to 1800 CE	*Katsuwonus pelamis*
Caribbean	Fairfield	1270-1420 A.D.	700 CE to 1800 CE	*Katsuwonus pelamis*, *Thunnus* sp.
Caribbean	Golden Grove	1700-900 BP	900 BCE to 700 CE	*Euthynnus alletteratus*, *Katsuwonus pelamis, Thunnus* sp.
Caribbean	Golden Rock	A.D. 400-600	900 BCE to 700 CE	*Euthynnus alletteratus*
Caribbean	Grand Bay	ca. AD 1000-1250, AD 1100-1300	700 CE to 1800 CE	*Scomberomorus* sp.
Caribbean	La Jacanas (PO-29)	AD 650-European contact, Prehistoric Contexts	700 CE to 1800 CE, Holocene	*Thunnus* sp.
Caribbean	Magens Bay	500 AD-1500 AD	700 CE to 1800 CE	*Euthynnus alletteratus*, *Scomberomorus* sp.
Caribbean	Maisabel Site	~ 100 BC - AD 500; ~ AD 1200	900 BCE to 700 CE	*Euthynnus alletteratus, Scomberomorus* sp., *Thunnus* sp.
Caribbean	Mamora Bay	post AD 500	700 CE to 1800 CE	*Thunnus* sp.
Caribbean	Marie Galante (Folle Anse)	220 B.C., A.D. 500-1000	700 CE to 1800 CE	*Auxis* sp., *Thunnus* sp.
Caribbean	Milford 1	3000-2800 BP	>900 BCE	*Euthynnus alletteratus*
Caribbean	Mill Reef	500-1000 A.D.	700 CE to 1800 CE	*Auxis* sp., *Thunnus* sp.
Caribbean	Point Macabou	Paquemar 500-750 & 900-1200/Macabou 1100-1456	Holocene	*Auxis* sp.
Caribbean	Rio Bueno (Bengal Estate)	post AD 500	700 CE to 1800 CE	*Thunnus* sp.
Caribbean	Robin Bay	500 BC - AD 500	900 BCE to 700 CE	*Euthynnus alletteratus*
Caribbean	Sabazan	ca. AD 850-1000, ca. AD 1000-1250, ca. AD 1250-1450	700 CE to 1800 CE	*Auxis* sp., *Euthynnus alletteratus, Katsuwonus pelamis, Thunnus* sp.
Caribbean	Santa Barbara	cal. AD 1045-1424	700 CE to 1800 CE	*Euthynnus alletteratus*
Caribbean	Sugar Factory Pier	~2500-700; 700 BC - AD 500	Holocene	*Auxis* sp., *Euthynnus alletteratus, Scomberomorus regalis, Scomberomorus* sp., *Thunnus* sp.

Caribbean	Taliseronde	300-600 AD	900 BCE to 700 CE	*Euthynnus alletteratus*, *Thunnus* sp.
Caribbean	Tanki Flip	cal. AD 775-1413	700 CE to 1800 CE	*Euthynnus alletteratus*
Caribbean	Trunk Bay	A.D. 900	700 CE to 1800 CE	*Euthynnus alletteratus*
Caribbean	White Marl	900-1500AD	700 CE to 1800 CE	*Thunnus* sp.
Mediterranean_Northeastern_Atlantic	*Central Mediterranean Tonnare	1911-1941 years CE	700 CE to 1800 CE	*Thunnus thynnus*
Mediterranean_Northeastern_Atlantic	1 Svendborg, Denmark	140,000 BCE	>900 BCE	*Thunnus thynnus*
Mediterranean_Northeastern_Atlantic	10 Grotte du Cap Ragnon, Bouche-du-Rhône, France	6,000-5,800 BCE	>900 BCE	*Thunnus thynnus*
Mediterranean_Northeastern_Atlantic	100 Saltes, Huelva, Spain	12th-14th CE	700 CE to 1800 CE	*Thunnus thynnus*
Mediterranean_Northeastern_Atlantic	101 Søborg, Gribskov, Denmark	13th-14th CE	700 CE to 1800 CE	*Thunnus thynnus*
Mediterranean_Northeastern_Atlantic	102 Monteleone, Sardinia, Italy	14th CE	700 CE to 1800 CE	*Thunnus thynnus*
Mediterranean_Northeastern_Atlantic	103 Ceuta, Spain	14th CE	700 CE to 1800 CE	*Thunnus thynnus*
Mediterranean_Northeastern_Atlantic	104 La Cartuja, Seville, Spain	14th-15th CE	700 CE to 1800 CE	*Thunnus thynnus*
Mediterranean_Northeastern_Atlantic	105 Raversijde, Oostende, Belgium	15th CE	700 CE to 1800 CE	*Thunnus thynnus*
Mediterranean_Northeastern_Atlantic	106 Tårnby Torv, Copenhagen, Denmark	13th-17th CE	700 CE to 1800 CE	*Thunnus thynnus*
Mediterranean_Northeastern_Atlantic	107 Pedras de Fogu, Sardinia, Italy	16th-18th CE	700 CE to 1800 CE	*Thunnus thynnus*
Mediterranean_Northeastern_Atlantic	108 Østergade, Gilleleje, Denmark	1600 CE	700 CE to 1800 CE	*Thunnus thynnus*
Mediterranean_Northeastern_Atlantic	109 Cappuccine, Sassari, Sardinia, Italy	17th CE	700 CE to 1800 CE	*Thunnus thynnus*
Mediterranean_Northeastern_Atlantic	11 Alepotrypa Cave, Greece	6,000-3,200 BCE	>900 BCE	*Thunnus thynnus*
Mediterranean_Northeastern_Atlantic	110 Ågabet, Bagenkop, Denmark	1550-1620 years CE	700 CE to 1800 CE	*Thunnus thynnus*
Mediterranean_Northeastern_Atlantic	111 Sassari, Sardinia, Italy	17th-19th CE	700 CE to 1800 CE	*Thunnus thynnus*
Mediterranean_Northeastern_Atlantic	112 Leca Harbour, Marseille, France	1750-1850 years CE	700 CE to 1800 CE	*Thunnus thynnus*
Mediterranean_Northeastern_Atlantic	113 Conil de la Frontera, Spain	1755 CE	700 CE to 1800 CE	*Thunnus thynnus*
Mediterranean_Northeastern_Atlantic	114 Palazzo Ducale, Sassari, Sardinia, Italy	19th CE	700 CE to 1800 CE	*Thunnus thynnus*
Mediterranean_Northeastern_Atlantic	12 Tågerup, Sweden	5,800 BCE	>900 BCE	*Thunnus thynnus*
Mediterranean_Northeastern_Atlantic	13 El Retamar, Spain	5,000 BCE	>900 BCE	*Thunnus thynnus*
Mediterranean_Northeastern_Atlantic	14 Lerna, Greece	5,300-2,200 BCE	>900 BCE	*Thunnus thynnus*
Mediterranean_Northeastern_Atlantic	15 Stora Förvar, Sweden	5,200-4,000 BCE	>900 BCE	*Thunnus thynnus*
Mediterranean_Northeastern_Atlantic	16 Saliagos Cave, Paros, Greece	4,300 BCE	>900 BCE	*Thunnus* sp.
Mediterranean_Northeastern_Atlantic	17 Pefkakia, Volos, Greece	4,300-2,500 BCE	>900 BCE	*Thunnus thynnus*
Mediterranean_Northeastern_Atlantic	18 Jortveit, Norway	3,500 BCE	>900 BCE	*Thunnus thynnus*
Mediterranean_Northeastern_Atlantic	19 Besik Tepe, Turkey	3,000-2,550 BCE	>900 BCE	*Thunnus thynnus*
Mediterranean_Northeastern_Atlantic	2 Gorham's Cave, Gibraltar	26,000-22,000 BCE	>900 BCE	*Thunnus thynnus*
Mediterranean_Northeastern_Atlantic	20 Perachora, Greece	2,650-2,200 BCE	>900 BCE	*Thunnus thynnus*
Mediterranean_Northeastern_Atlantic	21 Troy, Turkey	2,250-1,700 BCE	>900 BCE	*Thunnus thynnus*
Mediterranean_Northeastern_Atlantic	22 Gleika II, Crimea	2,000 BCE	>900 BCE	*Thunnus thynnus*

Mediterranean_Northeastern_Atlantic	23 Kommos, Greece	1,700-1,600 BCE	>900 BCE	*Thunnus thynnus*
Mediterranean_Northeastern_Atlantic	24 Akrotiri, Greece	1,500 BCE	>900 BCE	*Thunnus thynnus*
Mediterranean_Northeastern_Atlantic	25 Koukounaries, Paros, Greece	1,300-1,150 BCE	>900 BCE	*Thunnus thynnus*
Mediterranean_Northeastern_Atlantic	26 Skipshelleren, Norway	Neolithic	>900 BCE	*Thunnus thynnus*
Mediterranean_Northeastern_Atlantic	27 Ånneröd, Sweden	Neolithic	>900 BCE	*Thunnus thynnus*
Mediterranean_Northeastern_Atlantic	28 Gröninge, Sweden	Neolithic	>900 BCE	*Thunnus thynnus*
Mediterranean_Northeastern_Atlantic	29 Sandhem, Sweden	Neolithic	>900 BCE	*Thunnus thynnus*
Mediterranean_Northeastern_Atlantic	3 Cueva de Nerja, Malaga, Spain	10,000 BCE	>900 BCE	*Thunnus thynnus*
Mediterranean_Northeastern_Atlantic	30 Hakeröd, Sweden	Neolithic	>900 BCE	*Thunnus thynnus*
Mediterranean_Northeastern_Atlantic	31 Cova Fosca, Castellón, Spain	Neolithic	>900 BCE	*Thunnus thynnus*
Mediterranean_Northeastern_Atlantic	32 Utica, Tunisia	Punic	900 BCE to 700 CE	*Thunnus thynnus*
Mediterranean_Northeastern_Atlantic	33 Ceuta, Spain	8th-7th BCE	900 BCE to 700 CE	*Thunnus thynnus*
Mediterranean_Northeastern_Atlantic	34 Toscanos, Málaga, Spain	8th-7th BCE	900 BCE to 700 CE	*Thunnus thynnus*
Mediterranean_Northeastern_Atlantic	35 Carthage, Tunisia	8th-6th BCE	900 BCE to 700 CE	*Thunnus thynnus*
Mediterranean_Northeastern_Atlantic	36 Castillo de Doña Blanca, Spain	750-500 years BCE	900 BCE to 700 CE	*Thunnus thynnus*
Mediterranean_Northeastern_Atlantic	37 Tavira, Portugal	8th BCE-1st CE	900 BCE to 700 CE	*Thunnus thynnus*
Mediterranean_Northeastern_Atlantic	38 Huelva, Spain	8th BCE-1st CE	900 BCE to 700 CE	*Thunnus thynnus*
Mediterranean_Northeastern_Atlantic	39 Castro Marim, Portugal	8th-4th BCE	900 BCE to 700 CE	*Thunnus thynnus*
Mediterranean_Northeastern_Atlantic	4 Franchthi Cave, Greece	9,000 BCE	>900 BCE	*Thunnus thynnus*
Mediterranean_Northeastern_Atlantic	40 Althiburos, Tunisia	700-750 years BCE	900 BCE to 700 CE	*Thunnus thynnus*
Mediterranean_Northeastern_Atlantic	41 Acinipo, Spain	7th BCE	900 BCE to 700 CE	*Thunnus thynnus*
Mediterranean_Northeastern_Atlantic	42 Cerro del Villar Malaga, Spain	600-575 years BCE	900 BCE to 700 CE	*Thunnus thynnus*
Mediterranean_Northeastern_Atlantic	43 Chersonesos, Crimea	6th-5th BCE	900 BCE to 700 CE	*Thunnus thynnus*
Mediterranean_Northeastern_Atlantic	44 Corinth, Greece	460-440 years BCE	900 BCE to 700 CE	*Thunnus thynnus*
Mediterranean_Northeastern_Atlantic	45 Camposoto, Spain	450 years BCE	900 BCE to 700 CE	*Thunnus thynnus*
Mediterranean_Northeastern_Atlantic	46 Plaza de Asdrúbal, Spain	5th BCE	900 BCE to 700 CE	*Thunnus thynnus*
Mediterranean_Northeastern_Atlantic	47 Carthage, Tunisia	5th-4th BCE	900 BCE to 700 CE	*Thunnus thynnus*
Mediterranean_Northeastern_Atlantic	48 Carthage, Tunisia	5th-4th BCE	900 BCE to 700 CE	*Thunnus thynnus*
Mediterranean_Northeastern_Atlantic	49 Pilarou Cave, Thera, Santorini, Greece	5th-1st BCE	900 BCE to 700 CE	*Thunnus thynnus*
Mediterranean_Northeastern_Atlantic	5 Maroulas, Kythnos, Greece	8,800-8,600 BCE	>900 BCE	*Thunnus thynnus*
Mediterranean_Northeastern_Atlantic	50 Portopalo, Sicily, Italy	5th BCE-1st BCE	900 BCE to 700 CE	*Thunnus thynnus*
Mediterranean_Northeastern_Atlantic	51 Palacio de Justicia, Cadiz, Spain	5th BCE-2nd CE	900 BCE to 700 CE	*Thunnus thynnus*
Mediterranean_Northeastern_Atlantic	52 Kassopi, Greece,	4th BCE	900 BCE to 700 CE	*Thunnus thynnus*
Mediterranean_Northeastern_Atlantic	53 Place Jules Verne, Marseille, France	4th-3rd BCE	900 BCE to 700 CE	*Thunnus thynnus*
Mediterranean_Northeastern_Atlantic	54 Besik Tepe, Turkey	4th-1st BCE	900 BCE to 700 CE	*Thunnus thynnus*
Mediterranean_Northeastern_Atlantic	55 Vendicari, Sicily, Italy	4th BCE-1st CE	900 BCE to 700 CE	*Thunnus thynnus*

Mediterranean_Northeastern_Atlantic	56 Messene, Greece	3rd-2nd BCE	900 BCE to 700 CE	*Thunnus thynnus*
Mediterranean_Northeastern_Atlantic	57 Tamuda, Morocco	3rd BCE-1st CE	900 BCE to 700 CE	*Thunnus thynnus*
Mediterranean_Northeastern_Atlantic	58 Costa Rincon, Morocco	3rd BCE-1st CE	900 BCE to 700 CE	*Thunnus thynnus*
Mediterranean_Northeastern_Atlantic	59 Teatro Andalucía, Cádiz, Spain	3rd BCE-3rd CE	900 BCE to 700 CE	*Thunnus thynnus*
Mediterranean_Northeastern_Atlantic	6 Vela Spila, Korčula Island, Croatia	8,000 BCE	>900 BCE	*Thunnus thynnus*
Mediterranean_Northeastern_Atlantic	60 Pantikapaion, Crimea	250 years BCE	900 BCE to 700 CE	*Thunnus thynnus*
Mediterranean_Northeastern_Atlantic	61 Sant'Antioco, Sardinia, Italy	2nd BCE	900 BCE to 700 CE	*Thunnus thynnus*
Mediterranean_Northeastern_Atlantic	62 Kalaureia, Poros, Greece	2nd BCE	900 BCE to 700 CE	*Thunnus thynnus*
Mediterranean_Northeastern_Atlantic	63 Baelo Claudia, Spain	2nd BCE	900 BCE to 700 CE	*Thunnus thynnus*
Mediterranean_Northeastern_Atlantic	64 Punta Camarinal, Baelo Claudia, Spain	2nd BCE	900 BCE to 700 CE	*Thunnus thynnus*
Mediterranean_Northeastern_Atlantic	65 Baelo Claudia, Spain	2nd BCE	900 BCE to 700 CE	*Thunnus thynnus*
Mediterranean_Northeastern_Atlantic	66 Milazzo, Sicily, Italy	2nd BCE-2nd CE	900 BCE to 700 CE	*Thunnus thynnus*
Mediterranean_Northeastern_Atlantic	67 Baelo Claudia, Spain	1st BCE	900 BCE to 700 CE	*Thunnus thynnus*
Mediterranean_Northeastern_Atlantic	68 Titán Wreck, Isle of Levant, France	1st BCE	900 BCE to 700 CE	*Thunnus thynnus*
Mediterranean_Northeastern_Atlantic	69 Carthage, Tunisia	Pre-Roman	900 BCE to 700 CE	*Thunnus thynnus*
Mediterranean_Northeastern_Atlantic	7 Youra, Greece	7,700-6,900 BCE	>900 BCE	*Thunnus thynnus*
Mediterranean_Northeastern_Atlantic	70 Lixus, Morocco	Pre-Roman	900 BCE to 700 CE	*Thunnus thynnus*
Mediterranean_Northeastern_Atlantic	71 Cerro del Mar, Vélez-Málaga, Spain	1st BCE-1st CE	900 BCE to 700 CE	*Thunnus thynnus*
Mediterranean_Northeastern_Atlantic	72 Populonia, Italy	1st BCE-1st CE	900 BCE to 700 CE	*Thunnus thynnus*
Mediterranean_Northeastern_Atlantic	73 Lixus, Morocco	1st CE	900 BCE to 700 CE	*Thunnus thynnus*
Mediterranean_Northeastern_Atlantic	74 Olivillo, Cadiz, Spain	1st CE	900 BCE to 700 CE	*Thunnus thynnus*
Mediterranean_Northeastern_Atlantic	75 Castro Marim, Portugal	1st CE	900 BCE to 700 CE	*Thunnus thynnus*
Mediterranean_Northeastern_Atlantic	76 Chiessi Wreck, Isle of Elba, Italy	1st CE	900 BCE to 700 CE	*Thunnus thynnus*
Mediterranean_Northeastern_Atlantic	77 Carthage, Tunisia	1st-3rd CE	900 BCE to 700 CE	*Thunnus thynnus*
Mediterranean_Northeastern_Atlantic	78 Mytilene, Lesvos, Greece	1st-4th CE	900 BCE to 700 CE	*Thunnus thynnus*
Mediterranean_Northeastern_Atlantic	79 Puerta Califal, Ceuta, Spain	1st-5th CE	900 BCE to 700 CE	*Thunnus thynnus*
Mediterranean_Northeastern_Atlantic	8 Copenhagen, Denmark	6,800-5,400 BCE	>900 BCE	*Thunnus thynnus*
Mediterranean_Northeastern_Atlantic	80 Dion, Greece	2nd-3rd CE	900 BCE to 700 CE	*Thunnus thynnus*
Mediterranean_Northeastern_Atlantic	81 Baelo Claudia, Spain	5th-6th CE	900 BCE to 700 CE	*Thunnus thynnus*
Mediterranean_Northeastern_Atlantic	82 Teatro Romano, Malaga, Spain	5th CE	900 BCE to 700 CE	*Thunnus thynnus*
Mediterranean_Northeastern_Atlantic	83 Utica, Tunisia	Roman	900 BCE to 700 CE	*Thunnus thynnus*
Mediterranean_Northeastern_Atlantic	84 Carthage, Tunisia	5th CE	900 BCE to 700 CE	*Thunnus thynnus*
Mediterranean_Northeastern_Atlantic	85 Carteia, Spain	5th-6th CE	900 BCE to 700 CE	*Thunnus thynnus*
Mediterranean_Northeastern_Atlantic	86 San Nicolás, Algeciras, Spain	5th-6th CE	900 BCE to 700 CE	*Thunnus thynnus*
Mediterranean_Northeastern_Atlantic	87 Santa Filitica, Sardinia, Italy	5th-7th CE	900 BCE to 700 CE	*Thunnus thynnus*

Mediterranean_Northeastern_Atlantic	88 Sant'Imbenia, Sardinia, Italy	5th-7th CE	900 BCE to 700 CE	*Thunnus thynnus*
Mediterranean_Northeastern_Atlantic	89 İznik Roman Theatre, Turkey	5th-7th CE	900 BCE to 700 CE	*Thunnus thynnus*
Mediterranean_Northeastern_Atlantic	9 Cape Andreas Kastros, Cyprus	6,000 BCE	>900 BCE	*Thunnus thynnus*
Mediterranean_Northeastern_Atlantic	90 Gruissan, France	6th CE	900 BCE to 700 CE	*Thunnus thynnus*
Mediterranean_Northeastern_Atlantic	91 Carthage, Tunisia	6th-7th CE	900 BCE to 700 CE	*Thunnus thynnus*
Mediterranean_Northeastern_Atlantic	92 Yenikapi, Istanbul, Turkey	4th-15th CE	700 CE to 1800 CE	*Thunnus thynnus*
Mediterranean_Northeastern_Atlantic	93 Balat, Istanbul, Turkey	4th-15th CE	700 CE to 1800 CE	*Thunnus thynnus*
Mediterranean_Northeastern_Atlantic	94 Bathonea, Istanbul, Turkey	4th-15th CE	700 CE to 1800 CE	*Thunnus thynnus*
Mediterranean_Northeastern_Atlantic	95 Casale San Pietro, Sicily	8th-9th CE	700 CE to 1800 CE	*Thunnus thynnus*
Mediterranean_Northeastern_Atlantic	96 Sant'Antonino, Sicily	9th-10th CE	700 CE to 1800 CE	*Thunnus thynnus*
Mediterranean_Northeastern_Atlantic	97 Corso dei Mille, Sicily	9th-10th CE	700 CE to 1800 CE	*Thunnus thynnus*
Mediterranean_Northeastern_Atlantic	98 Mazara del Vallo, Sicily	10th-11th CE	700 CE to 1800 CE	*Thunnus thynnus*
Mediterranean_Northeastern_Atlantic	99 Mazara del Vallo, Sicily	13th CE	700 CE to 1800 CE	*Thunnus thynnus*
North_American_Pacific_Coast	45CA24	1510 CE	700 CE to 1800 CE	*Thunnus orientalis*
North_American_Pacific_Coast	CA-LAN-114	500 BCE-500 CE	900 BCE to 700 CE	*Thunnus alalunga*
North_American_Pacific_Coast	CA-LAN-211	1542-1830 CE	700 CE to 1800 CE	*Thunnus* sp.
North_American_Pacific_Coast	CA-LAN-227	500-1300 CE	700 CE to 1800 CE	*Thunnus alalunga*
North_American_Pacific_Coast	CA-LAN-229	1100-1830 CE	700 CE to 1800 CE	*Thunnus alalunga*
North_American_Pacific_Coast	CA-LAN-311	1100-1800 CE	700 CE to 1800 CE	*Thunnus alalunga*
North_American_Pacific_Coast	CA-LAN-47	1000-1750 CE	700 CE to 1800 CE	*Thunnus* sp.
North_American_Pacific_Coast	CA-LAN-52	500-1500 CE	700 CE to 1800 CE	*Thunnus alalunga, Thunnus orientalis*
North_American_Pacific_Coast	CA-LAN-54	6550-1050 BCE	>900 BCE	*Thunnus alalunga*
North_American_Pacific_Coast	CA-LAN-59	400-900 CE	Holocene	*Thunnus alalunga, Thunnus albacares, Thunnus* sp.
North_American_Pacific_Coast	CA-LAN-61A/B	1829 BCE-90 CE, 1030 BCE-670 CE	Holocene, 900 BCE to 700 CE	*Thunnus alalunga*, *Thunnus* sp.
North_American_Pacific_Coast	CA-LAN-62	Unknown	Holocene	*Thunnus* sp.
North_American_Pacific_Coast	CA-LAN-63	110-670 CE	900 BCE to 700 CE	*Thunnus alalunga, Thunnus* sp.
North_American_Pacific_Coast	CA-ORA-662	1300-1750 CE	700 CE to 1800 CE	*Thunnus* sp.
North_American_Pacific_Coast	CA-ORA-855	1400-1830 CE	700 CE to 1800 CE	*Thunnus alalunga, Thunnus* sp.
North_American_Pacific_Coast	CA-SBA-1703	4350-750 BCE	>900 BCE	*Thunnus* sp.
North_American_Pacific_Coast	CA-SBA-1731	1150-1550 CE	700 CE to 1800 CE	*Thunnus alalunga, Thunnus albacares, Thunnus* sp.
North_American_Pacific_Coast	CA-SBA-27	1070-1470 CE	700 CE to 1800 CE	*Thunnus alalunga*
North_American_Pacific_Coast	CA-SBA-28	5120 BCE-1820 CE	Holocene	*Thunnus alalunga*
North_American_Pacific_Coast	CA-SBA-46	1100-1650 CE	700 CE to 1800 CE	*Thunnus alalunga*
North_American_Pacific_Coast	CA-SBA-73	500-1500 CE	700 CE to 1800 CE	*Thunnus alalunga*
North_American_Pacific_Coast	CA-SCAI-106	585-1410 CE, 610-145 BCE	700 CE to 1800 CE, 900 BCE to 700 CE	*Thunnus alalunga, Thunnus albacares, Thunnus orientalis, Thunnus* sp.
North_American_Pacific_Coast	CA-SCAI-137	1280-1570 CE	700 CE to 1800 CE	*Thunnus* sp.

North_American_Pacific_Coast	CA-SCAI-17	4040-2850 BCE	>900 BCE	*Thunnus alalunga, Thunnus* sp.
North_American_Pacific_Coast	CA-SCAI-26	1520-1820 CE	700 CE to 1800 CE	*Thunnus alalunga*
North_American_Pacific_Coast	CA-SCAI-27	1050-1485 CE	700 CE to 1800 CE	*Thunnus alalunga, Thunnus albacares, Thunnus* sp.
North_American_Pacific_Coast	CA-SCAI-29	3050 BCE-1820 CE	Holocene	*Thunnus* sp.
North_American_Pacific_Coast	CA-SCAI-39	300-1820 CE	Holocene	*Thunnus* sp.
North_American_Pacific_Coast	CA-SCAI-564	435-1820 CE	Holocene	*Thunnus albacares*
North_American_Pacific_Coast	CA-SCLI-1215	460 CE	900 BCE to 700 CE	*Thunnus alalunga, Thunnus* sp.
North_American_Pacific_Coast	CA-SCLI-43/B/C	7300-2350 BCE, 7300 BCE-1360 CE, 1450 BCE-900 CE	>900 BCE, Holocene	*Thunnus alalunga, Thunnus albacares, Thunnus* sp.
North_American_Pacific_Coast	CA-SCRI-192	1300-1810 CE	700 CE to 1800 CE	*Thunnus* sp.
North_American_Pacific_Coast	CA-SCRI-236	1782-1810 CE	700 CE to 1800 CE	*Thunnus* sp.
North_American_Pacific_Coast	CA-SCRI-240	1700-1820 CE	700 CE to 1800 CE	*Thunnus* sp.
North_American_Pacific_Coast	CA-SCRI-474	550-1000 CE	700 CE to 1800 CE	*Thunnus alalunga*
North_American_Pacific_Coast	CA-SDI-10726	6050-4050 BCE	>900 BCE	*Thunnus* sp.
North_American_Pacific_Coast	CA-SDI-1103	4360-3070 BCE	>900 BCE	*Thunnus alalunga*
North_American_Pacific_Coast	CA-SDI-13325	6050 BCE-950 CE	Holocene	*Thunnus* sp.
North_American_Pacific_Coast	CA-SDI-4411	1000-1650 CE	700 CE to 1800 CE	*Thunnus* sp.
North_American_Pacific_Coast	CA-SDI-4538	1000-1650 CE	700 CE to 1800 CE	*Thunnus* sp.
North_American_Pacific_Coast	CA-SDI-811	6050-4050 BCE	>900 BCE	*Thunnus* sp.
North_American_Pacific_Coast	CA-SMI-525	1290 BCE-1430 CE	Holocene	*Thunnus* sp.
North_American_Pacific_Coast	CA-SNI-11	4850 BCE-1250 CE	Holocene	*Thunnus alalunga*
North_American_Pacific_Coast	CA-SNI-160	895-1860 CE	700 CE to 1800 CE	*Thunnus albacares*
North_American_Pacific_Coast	CA-SRI-97	1640-1815 CE	700 CE to 1800 CE	*Thunnus alalunga*
North_American_Pacific_Coast	CA-VEN-11	1500-1700 CE	700 CE to 1800 CE	*Thunnus* sp.
North_American_Pacific_Coast	CA-VEN-110	750-1650 CE	700 CE to 1800 CE	*Thunnus alalunga, Thunnus* sp.
North_American_Pacific_Coast	CA-VEN-168	630-1120 CE	700 CE to 1800 CE	*Thunnus alalunga*
North_American_Pacific_Coast	CA-VEN-294	6000 BCE-1400 CE	Holocene	*Thunnus* sp.
North_American_Pacific_Coast	CA-VEN-7	180-1400 CE	Holocene	*Thunnus alalunga*
North_American_Pacific_Coast	Covacha Babisuri	10000-6000 BCE, 6000-0 BCE	>900 BCE, Holocene	*Thunnus* sp.
North_American_Pacific_Coast	DJSp-1	2300 BCE-1880 CE	Holocene	*Thunnus orientalis*
North_American_Pacific_Coast	DJSp-3	Holocene	Holocene	*Thunnus orientalis*
North_American_Pacific_Coast	DcRt-10	400 BCE-800 CE	900 BCE to 700 CE	*Thunnus* sp.
North_American_Pacific_Coast	DfRw-1	2000 BCE-1000 CE	Holocene	*Thunnus orientalis*
North_American_Pacific_Coast	DfSg-1	800 BCE-300 CE	900 BCE to 700 CE	*Thunnus orientalis*
North_American_Pacific_Coast	DfSg-2	1700 CE	700 CE to 1800 CE	*Thunnus orientalis*
North_American_Pacific_Coast	DfSh-15	800-0 BCE	900 BCE to 700 CE	*Thunnus orientalis, Thunnus* sp.
North_American_Pacific_Coast	DfSh-7	3000 BCE-1600 CE	Holocene	*Thunnus orientalis*
North_American_Pacific_Coast	DfSi-16	200-1700 CE	Holocene	*Thunnus orientalis*, *Thunnus* sp.
North_American_Pacific_Coast	DfSi-4	2460 BCE-1880 CE	Holocene	*Thunnus orientalis*
North_American_Pacific_Coast	DfSj-100	2870-420 BCE	Holocene	*Thunnus orientalis*

North_American_Pacific_Coast	DfSj-23A/B	1175-1880 CE ; 760-1310 CE	700 CE to 1800 CE	*Thunnus orientalis*
North_American_Pacific_Coast	DgSl-67	800-1000 CE	700 CE to 1800 CE	*Thunnus orientalis*
North_American_Pacific_Coast	DhSe-2	500-820 CE	900 BCE to 700 CE	*Thunnus orientalis*
North_American_Pacific_Coast	DiSo-1	1230-1430 CE	700 CE to 1800 CE	*Thunnus orientalis*
North_American_Pacific_Coast	DjSi-5	1370 CE	700 CE to 1800 CE	*Thunnus orientalis*
North_American_Pacific_Coast	DkSp-1	1260-1460 CE	700 CE to 1800 CE	*Thunnus orientalis*
North_American_Pacific_Coast	DkSp-3	Holocene	Holocene	*Thunnus orientalis*
North_American_Pacific_Coast	EdSv-31	~500 BCE-1800 CE	Holocene	*Thunnus* sp.
North_American_Pacific_Coast	ElSx-1	4050-3050 BCE	>900 BCE	*Thunnus orientalis*
North_American_Pacific_Coast	FaTt-9	800-1800 CE	700 CE to 1800 CE	*Thunnus orientalis*
Southwestern_Indian_Ocean	Kizimkazi Dimbani	12th century	700 CE to 1800 CE	*Euthynnus affinis*
Southwestern_Indian_Ocean	Songo Mnara	14-15th centuries	700 CE to 1800 CE	*Euthynnus affinis, Katsuwonus pelamis, Scomberomorus commerson*
Southwestern_Indian_Ocean	Unguja Ukuu	650-1050	700 CE to 1800 CE	*Euthynnus affinis*

Literature Cited:

1. B. Collette, J. Graves, V. A. Kells, *Tunas and billfishes of the world* (Johns Hopkins University Press, 2019).
2. Z. Ali, Marine subsistence at St. John site in Trinidad: A preliminary study. *History in Action* **3**, 1-11 (2012).
3. D. W. Steadman, S. Jones, Long-term trends in prehistoric fishing and hunting on Tobago, West Indies. *Latin American Antiquity* **17**, 316-334 (2006).
4. C. I. R. Oliveira (2024) Zooarchaeological insights into traditional ecological knowledge and historical ecology of the Florida Keys. Thesis (University of Florida.).
5. A. E. Spiess, R. A. Lewis, *The Turner Farm fauna: 5000 years of hunting and fishing in Penobscot Bay, Maine*, Occasional Publications in Maine archaeology (The Maine State Museum, The Maine Historic Preservation Commission and the Maine Archaeological Society, Augusta, ME, 2001).
6. A. J. Andrews *et al.*, Exploitation history of Atlantic Bluefin Tuna in the eastern Atlantic and Mediterranean—insights from ancient bones. *ICES Journal of Marine Science* **79**, 247-262 (2022).
7. M. Burić, T. Težak Gregl, Navigare necesse est, vivere non est necesse: Reflections on prehistoric navigation in the Mediterranean and Adriatic. *Archaeologia Adriatica* **9**, 39-68 (2015).
8. K. Imamura, *Prehistoric Japan: New perspectives on insular east Asia* (UCL Press, 1996).
9. K. Shibata, K. Ota, M. Matsukawa, Reconstruction of the Jomon ecosystems in the Kanto region based on Yoshii and Kayama shell mounds, Hashidate Rock Shelter and Kasori shell mounds. *Science Report of the Yokosuka City Museum* **58**, 1-15 (2011).
10. A. B. J. Lambrides, M. I. Weisler, Late Holocene Marshall Islands archaeological tuna records provide proxy evidence for ENSO variability in the western and central Pacific ocean. *Journal of Island and Coastal Archaeology* **13**, 531-562 (2018).
11. J. R. Amesbury, R. L. Hunter-Anderson (2008) An analysis of archaeological and historical data on fisheries for pelagic species in Guam and the northern Mariana Islands. in *Pelagic Fisheries Research Program, University of Hawaii at Manoa.* (Micronesian Archaeological Research Services, Mangilao).
12. C. Boulanger, R. Ono, M. Intoh, M. Buckley, Zooms as a tool for understanding prehistoric pelagic fishing: Insights from archaeological shark and scombrid remains on Fais Island, Micronesia, over the last two millennia. *Journal of Archaeological Science* **184**, 106386 (2025).
13. S. O'Connor, R. Ono, C. Clarkson, Pelagic fishing at 42,000 years before the present and the maritime skills of modern humans. *Science* **334**, 1117-1121 (2011).
14. M. Beech (2004) In the land of the Ichthyophagi: Modelling fish exploitation in the Arabian Gulf and Gulf of Oman from the 5th millennium BC to the late islamic period. *British Archaeological Reports International Series* (Oxford).
15. H.-P. Uerpmann, M. Uerpmann, Stone Age sites and their natural environment. The capital area of northern Oman. Part iii. *Paléorient* **31**, 182-184 (2005).
16. G. Carenti, B. Wilkens, "Terrestrial fauna and marine produce in Sumhuram" in *Port in Arabia between Rome and the Indian Ocean, 3rd c. BC-5th c. AD: Khor rori report 2,* A. Avanzini, Ed. ("L'Erma" di Bretschneider, Roma, 2008).

17. M. E. Prendergast, E. M. Quintana Morales, A. Crowther, M. C. Horton, N. L. Boivin, Dietary diversity on the Swahili coast: The fauna from two Zanzibar trading locales. *International Journal of Osteoarchaeology* **27**, 621-637 (2017).
18. M. S. Lopes *et al.*, The path towards endangered species: Prehistoric fisheries in southeastern Brazil. *Plos One* **11**, e0154476 (2016).
19. A. B. Mendes, M. R. Duarte, E. P. Silva, Biodiversity of Holocene marine fish of the southeast coast of Brazil. *Biota Neotropica* **18**, e20170394 (2018).
20. P. Béarez, P. Gay, R. Lunniss, Sea fishing at Salango (Manabí province, Ecuador) during the middle formative Machalilla Phase. *Latin American Antiquity* **23**, 195-214 (2012).
21. P. Béarez, P. Stahl, "Las Vegas fish resources and their exploitation" in *Las Vegas: The early Holocene archaeology of human occupation in coastal ecuador* P. W. Stahl, K. E. Stothert, Eds. (Center for Comparative Archaeology, University of Pittsburgh, Pittsburgh, 2020), pp. 127-140.
22. P. Béarez, F. Fuentes-Mucherl, S. Rebolledo, D. Salazar, L. Olguín, Billfish foraging along the northern coast of Chile during the middle Holocene (7400–5900 cal BP). *Journal of Anthropological Archaeology* **41**, 185-195 (2016).
23. T. C. Rick *et al.*, Into the deep: Origins and evolution of northeastern Pacific Ocean tuna (*Thunnus* spp.) fisheries. *Fish and Fisheries*, 1-16 (2025).
24. J. Bernard, "Status and the swordfish: The origins of large-species fishing among the Chumash" in *Foundations of Chumash complexity* J. Arnold, Ed. (Cotsen Institute of Archaeology, UCLA, 2005), 25-51.
25. D. Davenport, J. R. Johnson, J. Timbrook, The Chumash and the swordfish. *Antiquity* **67**, 257-272 (1993).
26. A. J. Andrews *et al.*, Vertebrae reveal industrial-era increases in Atlantic Bluefin Tuna catch-at-size and juvenile growth. *ICES Journal of Marine Science* **80**, 836-847 (2023).
27. S. J. Crockford, Archeological evidence of large northern Bluefin Tuna, *Thunnus thynnus*, in coastal waters of British Columbia and northern Washington. *Fishery Bulletin* **95**, 11-24 (1997).
28. A. J. Andrews *et al.*, Length estimation of Atlantic Bluefin Tuna (*Thunnus thynnus*) using vertebrae. *International Journal of Osteoarchaeology* **32**, 645-653 (2022).
29. E. M. Quintana Morales, M. E. Prendergast, "Animals and their uses in the Swahili world" in *The Swahili world,* S. Wynne-Jones, A. LaViolette, Eds. (Routledge Taylor & Francis Group, 2017), pp. 335-349.
30. W. Van Neer, "Animal remains from the medieval site of Kizimkazi Dimhani, Zanzibar" in Islam in East Africa: New Sources (Archives, Manuscripts and Written Historical Sources, Oral History, Archaeology), B. S. Amoretti, Ed. (Rome, 2001), chap. Chapter, pp. 385-410.
31. J. B. Fleisher (2003) Viewing stonetowns from the countryside: An archaeological approach to Swahili regional systems, AD 800–1500. Thesis (University of Virginia, United States -- Virginia).
32. C. Shipton *et al.*, Reinvestigation of kuumbi cave, Zanzibar, reveals later Stone Age coastal habitation, early Holocene abandonment and Iron Age reoccupation. *Azania: Archaeological Research in Africa* **51**, 197-233 (2016).
33. A. Crowther *et al.*, Coastal subsistence, maritime trade, and the colonization of small offshore islands in eastern African prehistory. *Journal of Island and Coastal Archaeology* **11**, 211-237 (2016).

34. J. B. Fleisher *et al.*, When did the Swahili become maritime? *American Anthropologist* **117**, 100-115 (2015).
35. K. L. Van Niekerk (2011) Marine fish exploitation during the middle and later Stone Age of South Africa. Thesis (University of Cape Town).
36. K. N. Heidrich, J. J. Meeuwig, D. Zeller, Reconstructing past fisheries catches for large pelagic species in the Indian Ocean. *Frontiers in Marine Science* **10** (2023).
37. O. Longo, A trip among fish-eaters. *Newsletter of Baluchistan Studies* **4**, 11-17 (1987).
38. P. Biagi, R. Nisbet, The prehistoric fisher-gatherers of the western coast of the Arabian Sea: A case of seasonal sedentarization? *World Archaeology* **38**, 220-238 (2006).
39. P. Biagi, R. Nisbet, E. Starnini, "The prehistoric fishers and gatherers of the northern and western coasts of the Arabian Sea" in *The Arabian seas: Biodiversity, environmental challenges and conservation measures,* L. A. Jawad, Ed. (Springer International Publishing, Cham, 2021), pp. 47-78.
40. A. Marrast (2020) Strategies for the exploitation of arid coastal environments: Fishing activities from the Neolithic to the Bronze Age in the south-east of the Arabian Peninsula (Sultanate of Oman). Thesis (Museum National d'Histoire Nsaturelle - MNHN PARIS).
41. S. Cleuziou, M. Tosi, Ra's al-jinz and the prehistoric coastal cultures of the Ja'alan. *Journal of Oman Studies* **11**, 19-73 (2000).
42. M.A.F., *Fishery statistics book.* (Directorate General of Planning and Development, Department of Statistics and Information, Muscat, Sultanate of Oman, 2023).
43. K. Edyvane *et al.*, Conservation values, issues & planning in the Nino Konis Santana marine park, Timor Leste-final report. (2012).
44. S. O'Connor, New evidence from East Timor contributes to our understanding of earliest modern human colonisation east of the Sunda Shelf. *Antiquity* **81**, 523-535 (2007).
45. C. Shipton *et al.*, A new 44,000-year sequence from Asitau Kuru (Jerimalai), Timor-Leste, indicates long-term continuity in human behaviour. *Archaeological and Anthropological Sciences* **11**, 5717-5741 (2019).
46. C. Boulanger, S. Hawkins, S. Samper Carro, R. Ono, S. O'Connor, Continuity and variability in prehistoric fishing practices by *Homo sapiens* in island southeast Asia: New Ichthyofaunal data from Asitau Kuru, Timor-Leste. *World Archaeology* **54**, 288-316 (2022).
47. S. O'Connor, S. Kealy, C. Reepmeyer, S. C. Samper Carro, C. Shipton, Terminal Pleistocene emergence of maritime interaction networks across Wallacea. *World Archaeology* **54**, 244-263 (2022).
48. S. O'Connor *et al.*, Kisar and the archaeology of small islands in the Wallacean Archipelago. *Journal of Island and Coastal Archaeology* **14**, 198-225 (2019).
49. S. Kealy *et al.*, Forty-thousand years of maritime subsistence near a changing shoreline on Alor Island (Indonesia). *Quaternary Science Reviews* **249**, 106599 (2020).
50. S. C. Samper Carro, S. O'Connor, J. Louys, S. Hawkins, M. Mahirta, Human maritime subsistence strategies in the Lesser Sunda Islands during the terminal Pleistocene–early Holocene: New evidence from Alor, Indonesia. *Quaternary International* **416**, 64-79 (2016).
51. S. C. Samper Carro, J. Louys, S. O'Connor, Shape does matter: A geometric morphometric approach to shape variation in Indo-Pacific fish vertebrae for habitat identification. *Journal of Archaeological Science* **99**, 124-134 (2018).

52. S. O'Connor *et al.*, Fishing in life and death: Pleistocene fish-hooks from a burial context on Alor island, Indonesia. *Antiquity* **91**, 1451-1468 (2017).
53. P. Veth *et al.*, Early human occupation of a maritime desert, Barrow Island, north-west Australia. *Quaternary Science Reviews* **168**, 19-29 (2017).
54. M. G. Leavesley (2004) Trees to the sky: Prehistoric hunting in New Ireland, Papua New Guinea. Thesis (Australian National University, Canberra).
55. G. Kerby, A. Ford, G. R. Summerhayes, M. G. Leavesley, J. M. Palin, Fit for purpose: Investigating adaptations in late Pleistocene lithic technology to an island environment at Buang Merabak, New Ireland, Papua New Guinea. *World Archaeology* **54**, 317-337 (2022).
56. T. Manne, P. M. Veth, Late Pleistocene and early Holocene exploitation of estuarine communities in northwestern Australia. *Quaternary International* **385**, 112-123 (2015).
57. P. Veth, I. Ward, T. Manne, Coastal feasts: A Pleistocene antiquity for resource abundance in the maritime deserts of north west Australia? *Journal of Island and Coastal Archaeology* **12**, 8-23 (2017).
58. H. Lourandos, *Continent of hunter-gatherers: New perspectives in Australian prehistory* (Cambridge University Press, Cambridge, 1997).
59. I. J. McNiven, Saltwater people: Spiritscapes, maritime rituals and the archaeology of Australian Indigenous seascapes. *World Archaeology* **35**, 329-349 (2004).
60. S. Ulm, "Coastal foragers on southern shores: Marine resource use in northeast Australia since the late Pleistocene" in *Trekking the shore: Changing coastlines and the antiquity of coastal settlement,* N. F. Bicho, J. A. Haws, L. G. Davis, Eds. (Springer New York, New York, NY, 2011), pp. 441-461.
61. A. B. J. Lambrides, K. Ditchfield, S. Ulm, P. Veth, "A moving feast: Australia's coastal and island archaeological record" in *The Oxford handbook of island and coastal archaeology,* S. M. Fitzpatrick, J. M. Erlandson, Eds. (Oxford University Press, 2024), pp. 0.
62. S. Wickler (2001) The prehistory of Buka: A stepping stone island in the northern Solomons. Thesis (Australian National University, Canberra).
63. B. F. Leach, J. Davidson, "Fishing: A neglected aspect of oceanic economy" in *Australian archaeologist: Collected papers in honour of Jim Allen,* A. J. Anderson, and Murray, T., Ed. (Coombs Academic Publishing., Canberra, 2000), pp. 412-426.
64. A. B. J. Lambrides, M. I. Weisler, Pacific islands ichthyoarchaeology: Implications for the development of prehistoric fishing studies and global sustainability. *Journal of Archaeological Research* **24**, 275-324 (2016).
65. P. V. Kirch, T. S. Dye, Thno-archaeology and the development of Polynesian fishing strategies. *The Journal of the Polynesian Society* **88**, 53-76 (1979).
66. B. F. Leach, Fishing in pre-European New Zealand. *Archaeofauna*, 19-276 (2006).
67. M. S. Allen, "Spatial variability and human eco-dynamics in central east Polynesian fisheries" in *The Oxford handbook of zooarchaeology,* U. Albarella, M. Rizzetto, H. Russ, K. Vickers, S. Viner-Daniels, Eds. (Oxford University Press, 2017).
68. M. J. Rowland, B. Shaw, S. Ulm, "Maritime coastal and island societies of Australia and New Guinea" in *The Oxford handbook of the archaeology of Indigenous Australia and New Guinea,* I. J. McNiven, B. David, Eds. (Oxford University Press, Oxford, 2023), pp. 773–802.

69. I. J. McNiven, A. B. J. Lambrides, "Stone-walled fish traps of Australia and New Guinea as expressions of enhanced sociality" in *The Oxford Handbook of the Archaeology of Indigenous Australia and New Guinea,* I. J. McNiven, B. David, Eds. (Oxford University Press, Oxford, 2023), pp. 413-448.
70. C. M. Giovas, A. B. J. Lambrides, S. M. Fitzpatrick, O. Kataoka, Reconstructing prehistoric fishing zones in Palau, Micronesia using fish remains: A blind test of inter-analyst correspondence. *Archaeology in Oceania* **52**, 45-61 (2017).
71. L. Bouffandeau *et al.*, Fishing at Arapus-Mangaasi, Efate, Vanuatu (2800–2200 BP): New methodological approaches and results. *Journal of Archaeological Science: Reports* **18**, 356-369 (2018).
72. A. B. J. Lambrides, I. J. McNiven, S. Ulm, Meta-analysis of queensland's coastal Indigenous fisheries: Examining the archaeological evidence for geographic and temporal patterning. *Journal of Archaeological Science: Reports* **28**, 102057 (2019).
73. M. Harris, A. B. J. Lambrides, M. I. Weisler, Windward vs. Leeward: Inter-site variation in marine resource exploitation on Ebon Atoll, republic of the Marshall Islands. *Journal of Archaeological Science: Reports* **6**, 221-229 (2016).
74. V. Rurua (2020) Biodiversité et exploitation des ressources marines en Polynésie Française sur la longue durée: Étude comparative des Archipels des Marquises et des Gambier. Thesis (Université de la Polynésie française, Tahiti).
75. M. J. Carter (2004) North of the Cape and south of the fly: The archaeology of settlement and subsistence on the Murray Islands, eastern Torres Strait. Thesis (James Cook University).
76. R. Nims (2022) The archaeological ecodynamics of Māori fisheries: Climate change, harvest effects, and mass capture strategies in northern Aotearoa. Thesis (University of Auckland).
77. B. F. Leach, M. Intoh, I. Smith, Fishing, turtle hunting, and mammal exploitation at Fa'ahia, Huanine, French Polynesia. *Journal de la Société des Océanistes* **40**, 183-197 (1984).
78. B. F. Leach, J. Davidson, M. Horwood, P. Ottino, The fishermen of Anapua rock shelter, Ua Pou, Marquesas Islands. *Asian Perspectives* **36**, 51-66 (1997).
79. K. Fraser (1998) Fishing for tuna in Pacific prehistory. Thesis (University of Otago).
80. B. V. Rolett, *Hanamiai: Prehistoric colonization and cultural change in the Marquesas Islands, East Polynesia* (Yale University Press, New Haven, 1998).
81. J. M. Davidson, K. Fraser, B. F. Leach, Y. H. Sinoto, Prehistoric fishing at Hane, Ua Huka, Marquesas Islands, French Polynesia. *New Zealand Journal of Archaeology* **21**, 5-28 (1999).
82. R. Ono, M. Intoh, Island of pelagic fishermen: Temporal changes in prehistoric fishing on Fais, Micronesia. *Journal of Island and Coastal Archaeology* **6**, 255-286 (2011).
83. R. Ono, D. Addison Historical ecology and 600 years of fish use on Atafu Atoll, Tokelau. in *Prehistoric marine resource use in the Indo-Pacific regions*, R. Ono, A. E. Morrison, D. Addison, Eds. (ANU E Press, Canberra, 2013), pp 59-84.
84. B. F. Leach, Cranial allometry of the Pacific Albacore tuna, *Thunnus alalunga* and comparisons with three other species of Scombridae: An archaeological study. *Tuhinga* **37** (2026).

85. W. B. Masse, J. Liston, J. Carucci, J. S. Athens, Evaluating the effects of climate change on environment, resource depletion, and culture in the Palau islands between AD 1200 and 1600. *Quaternary International* **151**, 106-132 (2006).
86. M. I. Weisler, A. B. J. Lambrides, S. Quintus, J. Clark, T. Worthy, Colonisation and late period faunal assemblages from Ofu Island, American samoa. *Journal of Pacific Archaeology* **7**, 1-19 (2016).
87. A. B. J. Lambrides, M. Weisler, M. Charleux, New taxonomic records and regional trends for the Marquesan prehistoric marine fishery, Eiao Island, Polynesia. *Journal of Pacific Archaeology* **9**, 44-62 (2018).
88. M. Buckley, M. Pinsonneault, C. Brassey, B. Rolett, High-throughput microCT and ZooMS collagen fingerprinting of scombrid bone from the Marquesas Islands. *Journal of Archaeological Science* **136**, 105475 (2021).
89. M. I. Weisler *et al.*, Sacred offerings and secular foods on Reao Atoll, Tuamotu Archipelago, East Polynesia. *Archaeology in Oceania* **59**, 29-67 (2024).
90. P. H. Buck, *Material culture of Kapingamarangi*, Bernice Pauahi Bishop Museum Bulletin (Bishop Museum Press, Honolulu, HI, USA, 1950).
91. E. G. Burrows, M. E. Spiro, *An atoll culture: Ethnography of Ifaluk in the Central Carolines*, Behavior Science Monographs (Human Relations Area Files, New Haven, 1957), pp. 365.
92. A. Ohman, J. G. Kahn, Tracking shifts in society islands marine subsistence through time: Intra-site analysis of faunal remains and fishing gear. *Archaeology in Oceania* **59**, 198-218 (2024).
93. B. F. Leach, J. M. Davidson (2006) Analysis of faunal material from an archaeological site complex at Mangilao, Guam. in *Technical Report* (Museum of New Zealand Te Papa Tongarewa).
94. J. R. Amesbury, "Pelagic fishing in the Mariana Archipelago: From the prehistoric period to the present" in *Prehistoric marine resource use in the Indo-Pacific regions, terra australis,* R. Ono, Morrison, A., Addison, D. , Ed. (ANU Press, Canberra, 2013), pp. 33-57.
95. I. Pugach *et al.*, Ancient DNA from Guam and the peopling of the Pacific. *Proceedings of the National Academy of Sciences* **118**, e2022112118 (2021).
96. A. Caton *et al.*, "The Australian 1989–90 to 1994–95 Southern Bluefin Tuna seasons" in Scientific committee of the commission for the conservation of southern bluefin tuna. (Shimizu, Japan, 1995).
97. K. Barclay, "History of industrial tuna fishing in the Pacific islands" in *Historical Perspectives of Fisheries Exploitation in the Indo-Pacific,* J. Christensen, M. Tull, Eds. (Springer Netherlands, Dordrecht, 2014), pp. 153-171.
98. R. Gillett (2007) A short history of industrial fishing in the Pacific islands. (Asia-Pacific Fishery Commission, Food and Agriculture Organization of the United Nations, Regional Office for Asia and the Pacific, Bankok).
99. DCCEEW (2025) Assessment of the commonwealth eastern tuna and billfish fishery. (Australian Government Department of Climate Change, Energy, the Environment and Water, Canberra).
100. Ōshima-chō Kyōiku Iinkai (1987) Ōshima-chō nomashi iseki, shimotaka-bora iseki d chiku, izumihama c chiten iseki. (Ōshima-chō Board of Education, Ōshima-chō, Japan).

101. T. Akazawa, "Maritime adaptation of prehistoric hunter-gatherers and their transition to agriculture in Japan" in *Affluent Foragers: Pacific Coasts East and West,* S. Koyama, D. Thomas, Eds. (National Museum of Ethnology, Osaka, 1981), pp. 213-258.
102. T. Satō, A. Yoshino, Jōmon jidai ni okeru magurozoku no riyō: Iwate-ken miyano kaizuka shutsudo shiryō no kentō. *Shigaku* **85**, 379-399 (2015).
103. J. Uchiyama, "Why did northern foragers make pottery? Investigating the role of incipient Jomon ceramics within wider hunter-gatherer subsistence strategies in prehistoric Japan" in *Ceramics in circumpolar prehistory: Technology, lifeways and cuisine,* P. Jordan, K. Gibbs, Eds. (Cambridge University Press, 2019), pp. 17-36.
104. T. Nishimoto, The transition of hunting and fishing activities in Hokkaido. *Bulletin of the National Museum of Japanese History* **6**, 53-74 (1985).
105. M. Hudson, "The Ritsuryo State and bonito from Izu" in *The illustrated history of humankind,* G. Burenhult, Ed. (HarperCollins, 1994), vol. 4: New World and Pacific Civilizations, pp. 131.
106. N. Murakami *et al.*, Pottery lipids demonstrating marine product tribute in early historic Japan. *Asian Perspectives* **64**, 61-80 (2025).
107. T. Toizumi, "Gyoro katsudo no henkan" in *Hito to dobutsu no nihonshi,* T. Nishimoto, Ed. (Yoshikawa Kōbunkan, 2008), vol. 1: dōbutsu no kōkogaku, pp. 119-146.
108. F. G. Bock, The enthronement rites: The text of Engishiki, 927. *Monumenta Nipponica* **45**, 307-337 (1990).
109. J. C. Scott, *Against the grain: A deep history of the earliest states* (Yale University Press, 2017).
110. A. Sherratt, "Cash-crops before cash: Organic consumables and trade" in *The prehistory of food,* J. Hather, Ed. (Routledge, 1999), pp. 30-50.
111. C. Wickham, *Framing the early middle ages: Europe and the Mediterranean, 400-800* (Oxford University Press, 2005).
112. J. d'Alpoim Guedes, An archaeobotanical perspective on the relationship between grain crops, non-grain crops and states. *Cambridge Archaeological Journal* **29**, 694-696 (2019).
113. E. M. Brumfiel, T. K. Earle, Eds., *Specialization, exchange and complex societies* (Cambridge University Press, Cambridge, 1987).
114. W. W. Farris, *Sacred texts and buried treasures: Issues in the historical archaeology of ancient Japan* (University of Hawaii press, 1998).
115. H. Tsude (1989) Nihon noko shakai no seiritsu katei. (Iwanami, Tokoyo).
116. A. Seike, Ed., *Isoma iwakage iseki to hi-kinaisei to kōchisei* (Tanabe City Board of Education, 2021), pp 221-234.
117. H. Hara, "Suruga and Tōtōmi in the Kofun age" in *Capital and countryside in Japan, 300-1180: Japanese historians in english,* J. R. Piggott, Ed. (Cornell East Asia Series, 2006), pp. 77-101.
118. A. Marzano, *Harvesting the Sea: The exploitation of marine resources in the Roman Mediterranean* (Oxford University Press, Oxford, 2013).
119. A. Seike, Ed., *Isoma iwakage iseki no kenkyū* (Tanabe City Board of Education, 2021).
120. A. Seike (2024) The Isoma-Iwakage site: The burial site of a maritime community in 5th- and 6th-century Japan. Thesis (Okayama University).
121. C. Zancan *et al.*, A database of pictorial evidence of aquatic animals and fishing practices in prehistoric Japan. *Journal of Open Archaeology Data*, e21 (2025).

122. U. Iaccarino, *Comercio y diplomacia entre japón y filipinas en la era keichō, 1596-1615* (2017).
123. R. Cocks, *Diary of Richard Cocks* (1624).
124. J. Arch, "Sailing within sight of the land: Technology and japanese relationships with the Sea in the Tokugawa period" in *Oceanic Japan: The Archipelago in Pacific and global history,* S. Huebner, N. Heé, I. Miller, W. Tsutsui, Eds. (University of Hawaii Press, 2025), pp. 103-114.
125. J. Sakurai, "Archaeology of early-modern Japan: Food, rituals, and taboos" in *Handbook of east and southeast Asian archaeology*. (Springer, 2017), pp. 677-694.
126. J. V. García Marsilla, *Food consumption in medieval Iberia: A socio-economic analysis, 13th-15th centuries (1st ed.).* (Routledge, London, 2022).
127. A. Shimizu, *Specialty food, market culture, and daily life in early modern Japan* (Lexington Books, 2021).
128. A. Kalland, *Fishing villages in Tokugawa Japan* (University of Hawai'i Press, 1985).
129. H. Miyamoto, *Teichi amigyoron* (Kawade Shobō, 1944).
130. W. M. Tsutsui, "The pelagic empire: Reconsidering Japanese expansion" in *Japan at nature's edge: The environmental context of a global power,* I. J. Miller, J. A. Thomas, B. L. Walker, Eds. (University of Hawai'i Press, 2013), pp. 21-38.
131. E. C. Rath, *Oishii: The history of sushi* (Reaktion Books, 2021).
132. J. Kim, C. Seong, "Maritime prehistory of Korea: An archaeological review" in *Maritime prehistory of Northeast Asia,* J. Cassidy, I. Ponkratova, B. Fitzhugh, Eds. (Springer Nature Singapore, Singapore, 2022), pp. 29-50.
133. M. Kōmoto, Ed., *Kan higashi chūgokukai engan chiiki no senshi bunka [the prehistoric cultures of the circum east China Sea area].* (Kumamoto University, Kumamoto, 1998).
134. L. L. Sample, Tongsamdong: A contribution to Korean Neolithic culture history. *Arctic Anthropology* **11**, 1-125 (1974).
135. T. Kim, New foods on the block: Duck and tuna as new protein sources in South Korea in the 1980s. *The Korean Journal for the History of Science* **46**, 297-321 (2024).
136. T. Nakajima *et al.*, On the pharyngeal tooth remains of crucian and common carp from the Neolithic Tianluoshan site, Zhejiang province, China, with remarks on the relationship between freshwater fishing and rice cultivation in the Neolithic age. *International Journal of Osteoarchaeology* **22**, 294-304 (2012).
137. X. Qin (2021) Yulei yicun de kaoguxue yanjiu fangfa ji shijian — yi jiwanggu m1 suizang yulei yicun yanjiu wei li. Thesis (Wuhan University).
138. Dalian Shi Wenwu Kaogu Yanjiusuo (2000) Dazuizi: Qing tong shi dai yi zhi 1987 nian fa jue bao gao. (Dalian Shi Wenwu Kaogu Yanjiusuo, Dalian).
139. L. Qin, D. Fuller, Eds., *Why rice farmers don't sail: Coastal subsistence traditions and maritime trends in early China* (Springer, 2019), pp 159-191.
140. C. Xu, *Zhongguo yinshi shi* (Huaxia chubanshe, 2010).
141. L. Kuang-Ti, Prehistoric marine fishing adaptation in southern Taiwan. *Journal of East Asian Archaeology* **3**, 47-74 (2001).
142. Z. Chew (2024) An overview of prehistoric fisheries studies in Taiwan. Thesis (UC San Diego).
143. S. Qi, Cong kaogu faxian kan wo guo gudai buyu de qiyuan yu fazhan. *Nongye Kaogu* **2**, 220-225 (1986).

144. P. Edwards, Co-evolution of fish and rice farming by austroasiatic communities in the Neolithic era and early documentation of aquaculture in China. *Frontiers in Aquaculture* **3**, 1437833 (2025).
145. M. Fabinyi, N. Liu, Seafood banquets in Beijing: Consumer perspectives and implications for environmental sustainability. *Conservation and Society* **12**, 218-228 (2014).
146. Y. Hu *et al.*, An online survey study of consumer preferences on aquatic products in China: Current seafood consumption patterns and trends. *Fisheries and Aquaculture Journal* **5**, 1 (2014).
147. F. Asche *et al.*, China's seafood imports—not for domestic consumption? *Science* **375**, 386-388 (2022).
148. M. Kōmoto, Ed., *Kan higashi chūgokukai engan chiiki no senshi bunka, part ii, supplement.* (Kumamoto University., Kumamoto, 1999).
149. J. M. Erlandson, T. C. Rick, T. J. Braje, Fishing up the food web?: 12,000 years of maritime subsistence and adaptive adjustments on California's Channel Islands. *Pacific Science* **63**, 711-724 (2009).
150. T. L. Jones, K. W. Gobalet, B. F. Codding, The archaeology of fish and fishing on the central coast of California: The case for an underexploited resource. *Journal of Anthropological Archaeology* **41**, 88-108 (2016).
151. I. McKechnie, M. L. Moss, Meta-analysis in zooarchaeology expands perspectives on Indigenous fisheries of the northwest coast of North America. *Journal of Archaeological Science: Reports* **8**, 470-485 (2016).
152. J. Turnbull, K. W. Gobalet, J. W. Gaeta, M. R. Des Lauriers, The Native American fishery of Cedros Island, Baja California, and a comparison with the fisheries of the islands of the Southern California Bight. *Journal of California and Great Basin Anthropology*, 69-86 (2015).
153. R. A. Salls, To catch a fish: Some limitations on prehistoric fishing in southern California with special reference to native plant fiber fishing line. *Journal of Ethnobiology* **9**, 173-199 (1989).
154. H. D. Radde (2023) The origins of fishing economies and the evolution of fishing technologies on the California Channel Islands. Thesis (University of California, Santa Barbara, Santa Barbara, CA).
155. A. D. McMillan, Archaeological evidence for aboriginal tuna fishing on western Vancouver Island. *Syesis* **12**, 117-119 (1979).
156. H. Fujita, *Covacha babisuri: Doce mil años de prehistoria en la Isla Espíritu Santo* (Instituto Nacional de Antropología e Historia, 2023), info:doi.
157. M. Buckley, M. Collins, J. Thomas-Oates, A method of isolating the collagen (i) α2 chain carboxytelopeptide for species identification in bone fragments. *Analytical Biochemistry* **374**, 325-334 (2008).
158. T. C. Rick, V. L. Harvey, M. Buckley, Collagen fingerprinting and the Chumash billfish fishery, Santa Barbara Channel, California, USA. *Archaeological and Anthropological Sciences* **11**, 6639-6648 (2019).
159. E. A. Elliott Smith, P. Szpak, T. Braje, B. Newsom, T. Rick, Pre-industrial ecology and foraging behavior of swordfish (*Xiphias gladius*) in the eastern North Pacific. *Marine Ecology Progress Series* (2023).

160. A. F. Smith, *American tuna: The rise and fall of an improbable food* (University of California Press, 2012), vol. 37.
161. R. Lunniss, J. Zeidler, J. J. Ortiz Aguilú, "La transición Arcaico Tardío–Valdivia: Una reevaluación en base a evidencias de los sitios Salango y Valdivia." in *Valdivia, una sociedad neolítica: Nuevos aportes a su conocimiento,* M. B. Jadán Veriñez, Ed. (Ediciones Universidad Técnica de Manabí, Portoviejo, 2021).
162. P. Norton, R. Lunniss, N. Nayling, Excavaciones en Salango, provincia de Manabí. *Miscelánea Antropológica Ecuatoriana* **3**, 9-72 (1983).
163. C. Lacorne, R. Lunniss, P. Béarez, Massive offerings of marine fish at Salango, coastal Ecuador, during the middle Guangala Phase (AD300–600). *Journal of Island and Coastal Archaeology* **20**, 819-843 (2025).
164. J. Martínez-Ortiz, A. M. Aires-da-Silva, C. E. Lennert-Cody, M. N. Maunder, The Ecuadorian artisanal fishery for large pelagics: Species composition and spatio-temporal dynamics. *Plos One* **10**, e0135136 (2015).
165. E. J. Reitz, Fishing in Peru between 10000 and 3750 BP. *International Journal of Osteoarchaeology* **11**, 163-171 (2001).
166. P. Béarez, "Los peces y la pesca" in *Prehistoria de la costa extremo-sur del Perú. Los pescadores arcaicos de la quebrada de los burros (10000 - 7000 A.P),* D. L. M. Julien, Ed. (Pontificia Universidad Católica del Perú (PUCP) & Instituto Francés de Estudios Andinos (IFEA), Lima, 2012), pp. 99-123.
167. P. Béarez, L. Miranda, Análisis arqueo-ictiológico del sector residencial del sitio arqueológico de caral-supe, costa central del Perú. *Arqueología y Sociedad* **13**, 67-78 (2000).
168. L. Olguín, D. Salazar, D. Jackson, Tempranas evidencias de navegación y caza de especies oceánicas en la costa Pacífica de Sudamérica (Taltal, ~ 7.000 años cal. A.P.). *Chungará (Arica)* **46**, 177-192 (2014).
169. S. Rebolledo, P. Béarez, D. Salazar, F. Fuentes, Maritime fishing during the middle Holocene in the hyperarid coast of the Atacama Desert. *Quaternary International* **391**, 3-11 (2016).
170. C. Flores Fernandez, L. Olguín, "Swordfish hunting as prestige signaling?" in *Human behavioral ecology and coastal environments,* H. B. Thakar, C. Flores Fernandez, Eds. (University Press of Florida, 2023), pp. 132-148.
171. J. Escobar Gallegos, Caza tradicional de la albacora. Imaginario y patrimonio intangible de los pescadores de Taltal. *Taltalia* **10**, 69-85 (2017).
172. L. Bueno, A. S. Dias, J. Steele, The late Pleistocene/early Holocene archaeological record in Brazil: A geo-referenced database. *Quaternary International* **301**, 74-93 (2013).
173. A. F. Colombo, C. A. Joly, Brazilian Atlantic forest lato sensu: The most ancient Brazilian forest, and a biodiversity hotspot, is highly threatened by climate change. *Brazilian Journal of Biology* **70**, 697-708 (2010).
174. T. Ferraz *et al.*, Genomic history of coastal societies from eastern South America. *Nature Ecology and Evolution* **7**, 1315-1330 (2023).
175. B. S. Halpern *et al.*, Global priority areas for incorporating land–Sea connections in marine conservation. *Conservation Letters* **2**, 189-196 (2009).
176. M. D. Gaspar, P. DeBlasis, S. K. Fish, P. R. Fish, "Sambaqui (shell mound) societies of coastal Brazil" in *The handbook of South American archaeology,* H. Silverman, W. H. Isbell, Eds. (Springer New York, New York, NY, 2008), pp. 319-335.

177. J. Iriarte *et al.*, Out of Amazonia: Late-Holocene climate change and the Tupi–Guarani trans-continental expansion. *The Holocene* **27**, 967-975 (2017).
178. R. G. Milheira, C. Gianotti Garcia, "The earthen mounds (cerritos) of southern Brazil and Uruguay" in *Encyclopedia of global archaeology*. (Springer International Publishing, Cham, 2018), 10.1007/978-3-319-51726-1_3025-1, pp. 1-9.
179. R. G. Milheira, G. P. Wagner, *Arqueologia Guarani no litoral sul do Brasil*. R. G. Milheira, G. P. Wagner, Eds. (Editora Appris, 2014).
180. G. Wagner, K. Hilbert, D. Bandeira, M. C. Tenório, M. M. Okumura, Sambaquis (shell mounds) of the Brazilian coast. *Quaternary International* **239**, 51-60 (2011).
181. R. H. Nagai, S. H. M. Sousa, M. M. Mahiques, The southern Brazilian shelf. *Geological Society, London, Memoirs* **41**, 47-54 (2014).
182. R. J. Angulo, G. C. Lessa, M. C. d. Souza, A critical review of mid- to late-Holocene Sea-level fluctuations on the eastern Brazilian coastline. *Quaternary Science Reviews* **25**, 486-506 (2006).
183. T. d. F. Toniolo *et al.*, Sea-level fall and coastal water cooling during the late Holocene in southeastern Brazil based on vermetid bioconstructions. *Marine Geology* **428**, 106281 (2020).
184. A. C. Colonese *et al.*, Long-term resilience of late Holocene coastal subsistence system in southeastern South America. *Plos One* **9**, e93854 (2014).
185. T. Fossile, J. Ferreira, A. C. Colonese, Brazilian zooarch database: Banco de dados da fauna arqueológica do Brasil. *Revista de Arqueologia* **36**, 311-331 (2023).
186. T. Fossile *et al.*, Bridging archaeology and marine conservation in the neotropics. *Plos One* **18**, e0285951 (2023).
187. A. Toso *et al.*, Fishing intensification as response to late Holocene socio-ecological instability in southeastern South America. *Scientific Reports* **11**, 23506 (2021).
188. J. Ferreira, D. d. R. Bandeira, M. C. Bartz, T. Fossile, F. Mayorka, Reflexões sobre a pesca pré-colonial na baía da Babitonga, litoral norte de Santa Catarina, Brasil. *Cadernos do Lepaarq (Ufpel)* **16**, 138-155 (2019).
189. T. Fossile, J. C. de Sá, J. Ferreira, A. C. Colonese, "The onset of deep-water fishing in southern Brazil" in *Historical ecology and landscape archaeology in lowland South America,* A. C. Colonese, R. G. Milheira, Eds. (Springer International Publishing, Cham, 2023), pp. 205-218.
190. M. B. Guimarães, Fishing strategies among prehistoric populations at Saquarema Lagoonal Complex, Rio de Janeiro, Brazil. *Anais da Academia Brasileira de Ciências* **85**, 415-429 (2013).
191. J. de Sa, M. Gaspar, K. Macario, J. de Melo Junior, Experimental archaeology with plant fibers collected in the sambaquis of paleobaía (Guaratuba/PR and Babitonga/SC). *Habitus Journal - Journal of the Goiás Institute of Prehistory and Anthropology* **23**, 75-103 (2025).
192. M. C. Afonso, P. A. D. De Blasis, Aspectos da formação de um grande sambaqui: Alguns indicadores em Espinheiros II, Joinville. *Revista do Museu de Arqueologia e Etnologia*, 21-30 (1994).
193. S. P. Peixe, J. C. F. Melo Junior, D. da Rocha, Paleoetnobotânica dos macrorestos vegetais do tipo trançados de fibras encontrados no sambaqui Cubatão I, Joinville–SC. *Revista do Museu de Arqueologia e Etnologia*, 211-222 (2007).

194. K. McGrath *et al.*, Archaeological and molecular evidence for whale hunting in coastal Brazil 5000 years ago. *Nature Communications* **17**, 48 (2026).
195. A. B. Mendes, T. Rodrigues, Tetrapod biodiversity in sambaquis from southern Brazil. *Anais da Academia Brasileira de Ciências* **96**, e20230901 (2024).
196. R. Froese, D. Pauly (2025) Fishbase. (Fisheries Centre, University of British Columbia, World Wide Web electronic publication.).
197. P. G. C. d. Amaral, P. C. Fonseca Giannini, F. Sylvestre, L. C. Ruiz Pessenda, Paleoenvironmental reconstruction of a late Quaternary lagoon system in southern Brazil (Jaguaruna region, Santa Catarina state) based on multi-proxy analysis. *Journal of Quaternary Science* **27**, 181-191 (2012).
198. P. A. D. DeBlasis, A. Kneip, R. Scheel-Ybert, P. C. F. Giannini, M. D. Gaspar, Sambaquis e paisagem: Dinâmica natural e arqueologia regional no litoral do sul do Brasil. *Arqueología Suramericana/ Arqueologia Sul-Americana* **3**, 29-61 (2007).
199. P. C. F. Giannini *et al.*, Interactions between sedimentary evolution and prehistoric human occupation in the south-central coast of Santa Catarina, Brazil. *Boletim do Museu Paraense Emílio Goeldi. Ciências Humanas* **5**, 105-128 (2010).
200. L. A. Kuhn, K. A. Zonneveld, P. A. Souza, R. R. Cancelli, Late Quaternary palaeoenvironmental evolution and sea level oscillation of Santa Catarina island (southern Brazil). *Biogeosciences* **20**, 1843-1861 (2023).
201. L. C. Gerhardinger, D. F. Herbst, S. M. B. da Cunha, M. D. de Paula Costa, Diagnóstico da ictiofauna do ecossistema Babitonga. *Revista Cepsul-Biodiversidade e Conservação Marinha* **9**, eb2020001 (2020).
202. M. Haimovici, L. G. Cardoso, Long-term changes in the fisheries in the Patos Lagoon estuary and adjacent coastal waters in southern Brazil. *Marine Biology Research* **13**, 135-150 (2017).
203. T. Fossile *et al.*, Long-term perspective on fishing and mammal defaunation in the Atlantic forest coast of Brazil using archaeological faunal remains. *Tropical Conservation Science* **16**, 19400829231218419 (2023).
204. D. F. Herbst *et al.*, 180 years of marine animal diversity as perceived by public media in southern Brazil. *Plos One* **18**, e0284024 (2023).
205. S. L. Rodrigues, V. G. O. Carneiro, A. J. R. Freitas, H. A. Andrade, Spatiotemporal variability of target-species in tuna and tuna-like fishing in Brazil. *Boletim do Instituto de Pesca* **46** (2020).
206. J. J. Halligan *et al.*, Pre-clovis occupation 14,550 years ago at the Page-Ladson site, Florida, and the peopling of the Americas. *Science Advances* **2**, e1600375 (2016).
207. M. F. Napolitano *et al.*, Reevaluating human colonization of the Caribbean using chronometric hygiene and Bayesian modeling. *Science Advances* **5**, eaar7806 (2019).
208. T. Ardren *et al.*, Chronology building in the ancestral Florida Keys. *Southeastern Archaeology* **44**, 56-69 (2025).
209. M. F. Smith *et al.*, "Contributions of submerged archaeological research to the late Pleistocene and early Holocene record of the southeast" in *The American southeast at the end of the Ice Age,* D. Shane Miller, A. M. Smallwood, J. W. Tune, Eds. (University of Alabama Press, Tuscaloosa, 2022), pp. 213-229.
210. J. W. Cook Hale, N. L. Hale, E. G. Garrison, What is past is prologue: Excavations at the Econfina Channel Site, Apalachee Bay, Florida, USA. *Southeastern Archaeology* **38**, 1-22 (2019).

211. M. C. Sanger, J. W. Cook Hale, Recent middle and late Holocene transition research along the South Atlantic coastline. *Southeastern Archaeology* **44**, 83-100 (2025).
212. R. Saunders, M. Russo, Coastal shell middens in Florida: A view from the Archaic period. *Quaternary International* **239**, 38-50 (2011).
213. C. L. Hofman, A. J. Bright, M. L. P. Hoogland, Archipelagic resource procurement and mobility in the northern Lesser Antilles: The view from a 3000-year-old tropical forest campsite on Saba. *Journal of Island and Coastal Archaeology* **1**, 145-164 (2006).
214. W. F. Keegan *et al.*, Island colonization and human-environment interactions on the edges of the tropics: Archaeology of the Taíno frontier (northern Bahamas). *Journal of Field Archaeology* **48**, 297-314 (2023).
215. D. W. Steadman *et al.*, Late Holocene historical ecology: The timing of vertebrate extirpation on Crooked Island, commonwealth of the Bahamas. *Journal of Island and Coastal Archaeology* **12**, 572-584 (2017).
216. A. Palmiotto, A zooarchaeological synthesis of South Carolina's prehistoric coastal sites. *Southeastern Archaeology* **30**, 166-175 (2011).
217. C. D. Dillian, "Coastal adaptations in North and South Carolina" in *The archaeology of human-environmental dynamics on the North American Atlantic Coast,* L. Reeder-Myers, J. A. Turck, T. C. Rick, Eds. (University of Florida Press, Gainesville, 2019), pp. 137-163.
218. J. A. Jenkins, Methods for inferring oyster mariculture on Florida's Gulf Coast. *Journal of Archaeological Science* **80**, 74-82 (2017).
219. I. H. Lulewicz, V. D. Thompson, J. Cramb, B. Tucker, Oyster paleoecology and Native American subsistence practices on Ossabaw Island, Georgia, USA. *Journal of Archaeological Science: Reports* **15**, 282-289 (2017).
220. J. A. Rogers, T. J. Pluckhahn, K. Jackson, K. Gilmore, Multidimensional experiences of oystering: Investigating selectivity and management in contemporary and precolonial oyster assemblages from Tampa Bay, Florida, USA. *Journal of Ethnobiology* **45**, 115-128 (2025).
221. R. S. Carr, *Digging Miami* (University Press of Florida, Gianesville, 2023).
222. K. E. Sassaman *et al.*, The promise and peril of coastal infrastructure: Use life of a tidal fish trap on the Northern Gulf coast of Florida, circa AD400–650. *American Antiquity* **91**, 149-167 (2026).
223. V. D. Thompson *et al.*, Shellfishing, Sea levels, and the earliest Native American villages (5000–3800 yrs. BP) of the South Atlantic coast of the u.S. *Scientific Reports* **14**, 22322 (2024).
224. G. A. Waselkov *et al.*, History and hydrology: Engineering canoe canals in the estuaries of the Gulf of Mexico. *Journal of Field Archaeology* **47**, 486-500 (2022).
225. C. E. Colaninno, Examining Ichthyofaunal remains for evidence of fishing technologies employed in Georgia estuaries during the late Archaic period. *Southeastern Archaeology* **30**, 337-350 (2011).
226. E. J. Reitz, Availability and use of fish along coastal Georgia and Florida. *Southeastern Archaeology* **1**, 65-88 (1982).
227. E. J. Reitz, C. DePratter, Indigenous American fishing traditions at the first Spanish capital of la Florida: Santa Elena (1566–1587 ce), South Carolina, USA. *International Journal of Historical Archaeology* **28**, 576-611 (2024).

228. V. D. Thompson *et al.*, Ancient engineering of fish capture and storage in southwest Florida. *Proceedings of the National Academy of Sciences* **117**, 8374-8381 (2020).
229. C. J. S. Hadden (2015) Coastal subsistence and settlement systems on the Northern Gulf. Thesis (University of Georgia).
230. I. Holland-Lulewicz, Calusa fisheries and estuarine socio-ecologies in southwestern Florida: An examination of large-bodied fish. *Journal of Field Archaeology* **49**, 418-432 (2024).
231. K. L. Orr (2007) Coastal Weeden Island subsistence adaptations: Zooarchaeological evidence from Bayou St. John (1ba21), Alabama. Thesis (University of Georgia).
232. E. J. Reitz, C. E. Colaninno, I. R. Quitmyer, N. R. Cannarozzi, A 4,000-year record of multifaceted fisheries in the central Georgia Bight (USA). *Southeastern Archaeology* **41**, 253-270 (2022).
233. E. J. Reitz, C. S. Hadden, G. A. Waselkov, C. F. T. Andrus, Woodland-period fisheries on the north-central coast of the Gulf of Mexico. *Southeastern Archaeology* **40**, 135-155 (2021).
234. H. M. Singleton Hyde, N. J. Wallis, Coastal subsistence and platform mound feasting on Florida's Northern Gulf coast. *Journal of Island and Coastal Archaeology* **17**, 375-401 (2022).
235. K. J. Walker (1992) The zooarchaeology of Charlotte Harbor's prehistoric maritime adaptation: Spatial and temporal perspectives. Thesis (University of Florida).
236. M. Perkins (2020) Evidence of culinary practices in faunal data of site 38CH1531. Thesis (University of Tennessee Knoxville).
237. E. J. Reitz, M. A. Zierden, A zooarchaeological study of households and fishing in Charleston, South Carolina, USA, 1710–1900. *International Journal of Historical Archaeology* **25**, 1087-1112 (2021).
238. Caribbean Natural Resources Institute (CANARI) (2019) The Caribbean islands biodiversity hotspot. (Caribbean Natural Resources Institute (CANARI)).
239. M. E. Allen *et al.*, "Status and trends of coral reefs of the Caribbean region." in *Status of coral reefs of the world,* D. Souter *et al.*, Eds. (Global Coral Reef Monitoring network (GCRMN) and International Coral Reef Initiative (ICRI), 2020), chap. 12.
240. S. Grouard, "Caribbean archaeozoology" in *Current advances in Latin-American archaeozoology,* G. Mengoni Goñalons, J. Arroyo-Cabrales, J. Polaco Ó, F. J. Aguilar, Eds. (Instituto Nacional de Antropología e Historia, Consejo Nacional para la Ciencia y la Tecnología, México, International Council for Archaeozoology, Universidad de Buenos Aires, Mexico, 2010), pp. 133-150.
241. L. A. Newsom, E. S. Wing, *On land and Sea: Native American uses of biological resources in the West Indies.* (The University of Alabama Press, Tuscaloosa, 2004).
242. A. Stevens-Arroyo, "Taino religion" in *The Oxford handbook of Caribbean religions,* M. A. Gonzalez, Ed. (Oxford University Press, New York, 2024), pp. 3-16.
243. S. D. deFrance, "Zooarchaeology in the Caribbean: Current research and future prospects." in *The Oxford handbook of Caribbean archaeology* W. F. Keegan, C. L. Hofman, R. Rodríguez Ramos, Eds. (Oxford University Press, Oxford, 2013), pp. 378-390.
244. W. F. Keegan, L. A. Carlson, *Talking Taíno: Caribbean natural history from a native perspective* (The University of Alabama Press, Tuscaloosa, 2008).

245. N. Carder, J. G. Crock, A pre-columbian fisheries baseline from the Caribbean. *Journal of Archaeological Science* **39**, 3115-3124 (2012).
246. C. M. Connolly (2024) Exploring biases in Caribbean ceramic age legacy zooarchaeological data sets from a meta-analytic perspective. Thesis (University of Florida).
247. C. M. Giovas, Sustainable Indigenous fishing in the pre-contact Caribbean: Evidence and critical considerations from Carriacou, Grenada. *Sustainability* **13**, 9152 (2021).
248. S. Grouard, Faunal remains associated with Late Saladoïd and post-Saladoïd occupations at Anse à la Gourde, Guadeloupe, West Indies: Preliminary results. *Archaeofauna*, 71-98 (2001).
249. M. J. LeFebvre (2015) Animals, food, and social life among the pre-columbian Taíno of En Bas Saline, Hispaniola. Thesis (University of Florida).
250. S. Wing, E. Wing, Prehistoric fisheries in the Caribbean. *Coral Reefs* **20**, 1-8 (2001).
251. S. D. deFrance, "Saladoid and Ostionoid subsistence adaptations: Zooarchaeological data from a coastal occupation on Puerto Rico." in *Early ceramic population lifeways and adaptive strategies in the Caribbean,* P. E. Siegel, Ed. (British Archaeological Reports (BAR) Series, 1989), vol. 506, pp. 57-77.
252. E. S. Wing, E. J. Reitz, Prehistoric fishing economies of the Caribbean. *Journal of New World Archaeology* **5**, 13-32 (1982).
253. C. S. Henry (2018) Late pre-contact era Taíno subsistence economy and diet: Zooarchaeological perspectives from Maima. Thesis (Simon Fraser University).
254. C. Conlan, C. M. Giovas, The first zooarchaeological synthesis of the Leeward Antilles (Caribbean): Trends and implications for archaeology and conservation. *Environmental Archaeology* 10.1080/14614103.2026.2666441, 1-24 (2026).
255. C. M. Giovas, Pre-columbian amerindian lifeways at the Sabazan site, Carriacou, West Indies. *Journal of Island and Coastal Archaeology* **13**, 161-190 (2018).
256. J. R. Rooker *et al.*, Nursery origin of Yellowfin Tuna in the western Atlantic Ocean: Significance of Caribbean Sea and trans-Atlantic migrants. *Scientific Reports* **13**, 16277 (2023).
257. M. J. Hardt, Lessons from the past: The collapse of Jamaican coral reefs. *Fish and Fisheries* **10**, 143-158 (2009).
258. R. Price, Caribbean fishing and fishermen: A historical sketch. *American Anthropologist* **68**, 1363-1383 (1966).
259. M. J. A. Vermeij *et al.*, Historical changes (1905-present) in catch size and composition reflect altering fisheries practices on a small Caribbean island. *Plos One* **14**, e0217589 (2019).
260. S. Apollonio, The Gulf of Maine. *Courier of Maine Books, Rockland* (1979).
261. J. Shaw *et al.*, Catastrophic tidal expansion in the Bay of Fundy, Canada. *Canadian Journal of Earth Sciences* **47**, 1079-1091 (2010).
262. B. J. Bourque, *Diversity and complexity in prehistoric maritime societies: A Gulf of Maine perspective* (Springer, 1995).
263. A. E. Spiess, F. Price, Offshore artifacts: Paleoindian and Archaic use of the Gulf of Maine. *Northeast Anthropology* **93-94**, 43-72 (2023).
264. B. J. Bourque, *The swordfish hunters: The history and ecology of an ancient American Sea people* (Bunker Hill Publishing, Piermont, New Hampshire, 2012).

265. B. S. Robinson, *The Nelson Island and Seabrook Marsh sites: Late Archaic, marine oriented people on the central New England coast*, Occasional publications in northeastern anthropology (Fund for Anthropology, Department of Anthropology, Franklin Pierce College, 1985), vol. 9.
266. S. L. Cox, Site 95.20 and the Vergennes phase in Maine. *Archaeology of Eastern North America*, 135-161 (1991).
267. A. E. Spiess, J. Mosher (2005) Ntolonapemk/Eastern Surplus superfund site (96.2) faunal identification report. in *The Archaeology of N'tolonapemk (96.02 Me), 'Our Ancestor's Place': Phase Iii Data Recovery at the Eastern Surplus Company Superfund Site, Meddybemps, Washington Co, Maine*, eds M. S. Brigham *et al.*
268. J. T. Kelley, D. F. Belknap, S. Claesson, Drowned coastal deposits with associated archaeological remains from a Sea-level "slowstand": Northwestern Gulf of Maine, USA. *Geology* **38**, 695-698 (2010).
269. J. T. Kelley, D. F. Belknap, A. R. Kelley, S. H. Claesson, A model for drowned terrestrial habitats with associated archeological remains in the northwestern Gulf of Maine, USA. *Marine Geology* **338**, 1-16 (2013).
270. K. Brown, D. A. Fa, G. Finlayson, C. Finlayson, "Small game and marine resource exploitation by Neanderthals: The evidence from Gibraltar" in *Trekking the shore: Changing coastlines and the antiquity of coastal settlement,* N. F. Bicho, J. A. Haws, L. G. Davis, Eds. (Springer New York, New York, NY, 2011), pp. 247-272.
271. A. Anderson, S. O'Connor, R. Ono, G. N. Bailey, J. M. Erlandson, Debate: Inshore or offshore? Boating and fishing in the Pleistocene. *Antiquity* **87**, 879-885 (2013).
272. M. Rose, Fishing at Franchthi Cave, Greece: Changing environments and patterns of exploitation. *Old World Archaeology Newsletter* **18**, 21-26 (1995).
273. C. Rainsford, T. O'Connor, P. Miracle, Fishing in the Adriatic at the Mesolithic–Neolithic transition: Evidence from Vela Spila, Croatia. *Environmental Archaeology* **19**, 311-320 (2014).
274. J. D. Evans, C. Renfrew (1968) Excavations at Saliagos near Antiparos. in *The British School in Athens* (Oxford).
275. A. Morales Muñiz, E. Roselló Izquierdo, "Fishing in Mediterranean prehistory: An archaeo-ichthyological overview" in *The inland seas: Towards an ecohistory of the Mediterranean and the Black Sea,* T. Bekker-Nielsen, R. Gertwagen, Eds. (Franz Steiner Verlag, Stuttgart, 2016), vol. 35.
276. C. Pickard, C. Bonsall, Deep-Sea fishing in the European Mesolithic: Fact or fantasy? *European Journal of Archaeology* **7**, 273-290 (2004).
277. D. Mylona, Aquatic animal resources in prehistoric Aegean, Greece. *Journal of Biological Research-Thessaloniki* **21**, 2 (2014).
278. C. Perlès, "The Mesolithic at franchthi: An overview of the data and problems" in *The Greek Mesolithic: Problems and perspectives* N. Galanidou, C. Perlès, Eds. (British School at Athens, Athens, 2008), vol. 10, pp. 75-88.
279. D. Mylona, Catching tuna in the Aegean: Biological background of tuna fisheries and the archaeological implications. *Anthropozoologica* **56**, 23-37, 15 (2021).
280. E. F. Eriksen *et al.*, Five millennia of mitonuclear discordance in Atlantic Bluefin Tuna identified using ancient DNA. *Heredity* **134**, 175-185 (2025).

281. A. J. Andrews *et al.*, Ancient DNA suggests a historical demographic decline and genetic erosion in the Atlantic Bluefin Tuna. *Proceedings of the National Academy of Sciences* **122**, e2409302122 (2025).
282. A. J. Andrews *et al.*, Exploitation shifted trophic ecology and habitat preferences of Mediterranean and Black Sea Bluefin Tuna over centuries. *Fish and Fisheries* **24**, 1067-1083 (2023).
283. M. J. Juan-Jordá, I. Mosqueira, A. B. Cooper, J. Freire, N. K. Dulvy, Global population trajectories of tunas and their relatives. *Proceedings of the National Academy of Sciences* **108**, 20650-20655 (2011).
